# Comparability of Liquid Chromatography Tandem Mass Spectrometry Analysis of Dissolved Organic Matter across Laboratories

**DOI:** 10.1021/acs.est.5c12691

**Published:** 2026-02-06

**Authors:** Jarmo-Charles Kalinski, Bruno Ruiz Brandão da Costa, Tilman Schramm, Lance R. Buckett, Laura T. Carlson, Nicole R. Coffey, Tito Damiani, Elias Dechent, Yasin El Abiead, Steffen Heuckeroth, Elaine K. Jennings, Jan Kaesler, Naomi L. Stock, Alice M. Orme, Ralph R. Torres, Sara Trojahn, Helen L. Whelton, Yingfei Yan, Allegra T. Aron, Rene M. Boiteau, Ian D. Bull, Pieter C. Dorrestein, Duc Huy Dang, Richard P. Evershed, Marta Gledhill, Gerd Gleixner, Andreas F. Haas, Martin Hansen, Tilmann Harder, Ellen C. Hopmans, Anitra E. Ingalls, Uwe Karst, William Kew, Melissa Kido Soule, Boris P. Koch, Elizabeth B. Kujawinski, Oliver J. Lechtenfeld, Krista Longnecker, Tomáš Pluskal, Georg Pohnert, Zachary C. Redman, Albert Rivas-Ubach, Philippe Schmitt-Kopplin, Gabriel Singer, Jan Tebben, Patrick L. Tomco, Nicholas D. Ward, Lihini I. Aluwihare, Carsten Simon, Jeffrey Hawkes, Daniel Petras

**Affiliations:** † Department of Biochemistry, 8790 University of California Riverside, Riverside, California 92521, United States; ‡ Rhodes University, Grahamstown (Makhanda) 6140, South Africa; § University of São Paulo, São Paulo 05508-000, Brazil; ∥ Helmholtz Munich, Analytical BioGeoChemistry, Neuherberg 85764, Germany; ⊥ School of Oceanography, 7284 University of Washington, Seattle, Washington 98195, United States; # Department of Earth and Environmental Sciences, 5635 University of Minnesota, Minneapolis, Minnesota 55455, United States; 7 89220 Institute of Organic Chemistry and Biochemistry, Czech Academy of Sciences, Prague 160 00, Czech Republic; 8 Department of Ecology, University of Innsbruck, Innsbruck 6020, Austria; 9 Skaggs School of Pharmacy, 8784 University of California San Diego, La Jolla, California 92093, United States; 10 Institute of Inorganic and Analytical Chemistry, University of Münster, Münster 48149, Germany; 11 Helmholtz Centre for Environmental Research (UFZ), Leipzig 04318, Germany; 12 Water Quality Centre, 6515 Trent University, Peterborough, Ontario K9L 0G2, Canada; 13 28300 Max Planck Institute for Biogeochemistry, Jena 07745, Germany; 14 Institute of Inorganic and Analytical Chemistry, 9378 Friedrich Schiller University Jena, Jena 07743, Germany; 15 Scripps Institution of Oceanography, University of California San Diego, La Jolla, California 92037, United States; 16 The James Hutton Institute, Aberdeen AB15 8QH, U.K.; 17 School of Chemistry, 1980 University of Bristol, Bristol BS8 1TS, U.K.; 18 Department of Chemistry, 2927 University of Denver, Denver, Colorado 80208, United States; 19 Department of Chemistry, University of Minnesota, Minneapolis, Minnesota 55455, United States; 20 Trent School of the Environment and Chemistry Department, Trent University, Peterborough, Ontario K9L 0G2, Canada; 21 GEOMAR Helmholtz Centre for Ocean Research, Kiel 24148, Germany; 22 NIOZ Royal Netherlands Institute for Sea Research, Den Burg (Texel) 1790 AB, The Netherlands; 23 Department of Environmental and Resource Engineering, 5205 Technical University of Denmark, Kongens Lyngby 2800, Denmark; 24 Alfred Wegener Institute, Bremerhaven 27570, Germany; 25 6865 Pacific Northwest National Laboratory, Richland, Washington 99354, United States; 26 10627 Woods Hole Oceanographic Institution, Woods Hole, Massachusetts 02543, United States; 27 University of Applied Sciences Bremerhaven, Bremerhaven 27568, Germany; 28 Department of Chemistry, 3291 University of Alaska Anchorage, Anchorage, Alaska 99508, United States; 29 54402 Instituto Nacional de Investigación y Tecnología Agraria y Alimentaria (INIA-CSIC), Madrid 28040, Spain; 30 Eawag, Swiss Federal Institute of Aquatic Science and Technology, Dübendorf 8600, Switzerland; 31 Inorganic Environmental Geochemistry, Institute of Biogeochemistry and Pollutant Dynamics, ETH Zurich, Zurich 8092, Switzerland; 32 Department of Chemistry, Uppsala University, Uppsala 751 05, Sweden; 33 CMFI Cluster of Excellence, University of Tübingen, Tübingen 72074, Germany

**Keywords:** dissolved organic matter, DOM, high resolution tandem mass spectrometry, LC–MS/MS, non-targeted analysis, non-targeted metabolomics, structure-resolved chemical analysis, interlaboratory comparison

## Abstract

Non-targeted liquid chromatography tandem high-resolution mass spectrometry (LC–MS/MS) is increasingly applied for the structure-resolved chemical analysis of dissolved organic matter (DOM). With new developments in MS instrumentation and analysis software, the approach has gained substantial momentum over the past decade. However, achieving high-quality analytical data that is reproducible and comparable across laboratories can be a bottleneck in non-targeted metabolomics and organic matter chemical analysis, especially for data reuse in repository-scale analyses. Understanding the capabilities as well as challenges of comparing LC–MS/MS data from different laboratories is necessary for inferring global trends from public data sets. To illuminate instrumentation factors that drive differences and variability, we used a standardized data analysis pipeline, including classical (CMN) and feature-based molecular networking (FBMN), to analyze data from a ring trial by 24 laboratories on identical sample sets of algal and DOM extracts that were mixed in predefined concentrations and spiked with standards. Our results showed that data sets from similar mass spectrometer types with unified instrument parameters were qualitatively comparable, resolving the same general trends and shared mass spectral features. Interlaboratory comparability was best for high-intensity features, while low-intensity features showed greater detection variability. Our analysis also highlights challenges when comparing data from instruments with different acquisition rates or operating with less standardized methods. Lastly, we provide recommendations for data integration, public data sharing, standardization, and best practices for standardized LC–MS/MS data acquisition, which will be critical for long-term time series and intercomparability of DOM chemical analyses.

## Graphical abstract

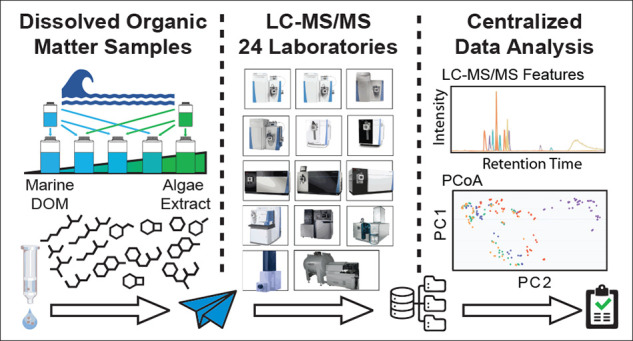

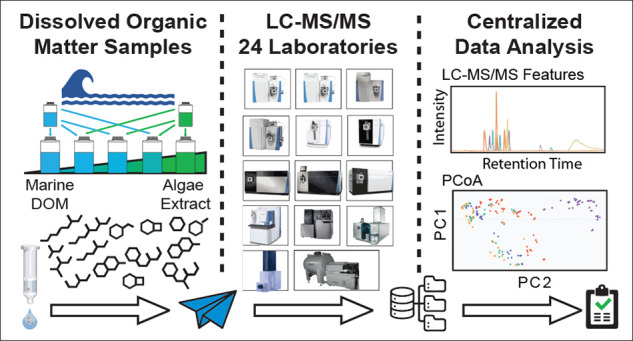

## Introduction

The production, transformation, transport, and accumulation of organic matter (OM) throughout Earth’s biosphere play a central role in regulating the global carbon cycle and shaping ecosystem food webs.^1,2^ Dissolved organic matter (DOM) is particularly important when considering exchanges of OM across land-river-ocean interfaces and transformations that occur as DOM undergoes biotic and abiotic reworking within and across ecosystems.^3,4^ Many fields focused on individual components of the Earth system, including soil science,^5^ limnology,^6^ and coastal science,^7^ use similar mass spectrometry tools to decipher the composition of DOM and its role in biogeochemical cycles.

Recent advances in targeted and non-targeted liquid chromatography high-resolution tandem mass spectrometry (LC–MS/MS)-based methods have improved the study of DOM, providing expanded structural details that aid in uncovering its ecological roles.^8–14^ Yet, substantial variability arises in the analytical strategies and data processing workflows employed during LC–MS/MS analysis. For instance, HR–MS/MS platforms range from quadrupole time-of-flight (QTOF)^15,16^ and Orbitrap^17,18^ to Fourier transform ion cyclotron resonance (FT–ICR)^19^ mass spectrometers, which exhibit different sensitivity, resolution, and mass accuracy. These differences are further amplified by variations in sample introduction and ionization techniques, such as electrospray ionization (ESI) and direct infusion versus LC separation.^20–22^ On top of this, the choice of MS/MS data acquisition approaches, i.e., data-dependent acquisition (DDA) or data-independent acquisition (DIA),^23^ further contributes to methodological discrepancies, and different settings can drastically alter analysis results.^24^ Subsequent data analysis pipelines for LC–MS/MS based non-targeted metabolomics are equally heterogeneous, reflecting differences in software packages, feature-finding algorithms, spectral matching, and annotation criteria.^25^

The variability introduced by the various analytical approaches complicates efforts to interpret and compare the DOM composition at local, regional, and global scales. In practical terms, this means that two laboratories studying DOM in different ocean basins, or monitoring the same site over different time frames, may not be able to tell whether observed differences reflect real environmental change or simply differences in instrument settings and data processing. While different analytical practices broaden research capabilities, standardized methods are particularly important when long-term comparability is required, such as in ocean time-series and other monitoring efforts. In this context, interlaboratory ring trials have emerged as a critical approach to evaluate the comparability of data derived from shared samples.^26^ These trials are essential for addressing current challenges in the field, improving reproducibility, and ensuring reliable cross-laboratory comparisons.

An initial effort in this regard was made by investigating the consistency of DOM analyses across laboratories using direct infusion HR–MS.^27^ However, a gap remains in exploring interlaboratory comparisons for other emerging methods in DOM analysis, particularly LC–MS/MS-based non-targeted metabolomics workflows, which introduce additional analytical variation due to chromatographic separation, tandem MS acquisition settings, and instrument-specific capabilities. This gap is particularly important because LC–MS/MS is increasingly used to generate structure-resolved DOM fingerprints in global surveys and time series and is beginning to be embedded in large coordinated efforts, where data sets from many laboratories must be integrated to assess how marine ecosystems respond to climate and anthropogenic change. Evaluating the current state of LC–MS/MS approaches (limited herein to DDA) in DOM analysis is thus both timely and essential in this stage of tool development.

Here, we report on a ring study of DOM, using standardized ultrahigh-performance liquid chromatography (UHPLC), followed by electrospray ionization (ESI) in positive (ESI+) and negative (ESI-) modes and DDA of MS/MS spectra. The study focused on addressing three key questions: (1) Do laboratories detect the same molecular features from a shared sample set under consistent instrumental conditions? (2) Do their data lead to the same qualitative conclusions? (3) Is it meaningful to align and coanalyze the data sets?

To address these questions, we generated a set of simulated algal bloom extracts in which we combined a serial dilution of algae extracts with DOM extracts from marine surface water collected in Southern California (Scripps Research Pier, La Jolla, CA, USA). These extracts and mixtures of them were shipped to participating laboratories and analyzed with their respective instrumentation. The resulting data were analyzed using a standardized analysis pipeline using classic and feature-based molecular networking (CMN and FBMN) approaches to assess the overlap in the metabolite annotations. CMN aligns features by the similarity of their MS/MS spectra (cosine similarity), and FBMN aligns them based on extracted ion chromatograms defined by exact mass and retention time (RT) and generates consensus MS/MS spectra between all features (given a defined minimum MS/MS similarity).^28^ For both CMN and FBMN, multivariate statistics and feature intensity trends were applied to explore global differences between data sets. We assessed whether non-targeted metabolomics results from DOM are qualitatively (presence-absence) and semiquantitatively (compositional trends) comparable between laboratories using similar instrument platforms and standardized instrument parameters.

Our data shows that results from instruments with similar performance characteristics resolved the same general trends between sample types and identified similar sets of shared metabolite features. For ESI+, interlaboratory data was qualitatively comparable for high-intensity features, while low-intensity features showed greater variability. These trends were less pronounced for ESI-, yet qualitative distinction of sample types was still achieved by most laboratories. Together, these results demonstrate that interlaboratory comparison of LC–MS/MS data can provide converging results when processed together using both MS/MS-based clustering (CMN) as well as mass–retention time (*m*/*z*–RT) alignment (FBMN) for similar instrument types with unified methods. On the flipside, our results also highlight a high degree of variation when comparing data from instruments with different designs and analytical capabilities or less stringent instrument method standardization. Our findings underscore the need for harmonized LC–MS/MS data acquisition methods and the development of dedicated data processing workflows that enable the integration of local measurements across laboratories and experiments.

## Material and Methods

### DOM Extract Preparation

200 L of surface seawater were collected at the end of the Ellen Browning Scripps Memorial Pier, San Diego, California, USA, on February 26, 2021, between 11:00 and 19:00 PDT. The seawater was filtered through an AcroPak 0.8/0.45 μm Supor membrane filter (Pall Corporation) and subsequently acidified to a pH of 2 with a total volume of 260 mL HCl (37%, trace metal grade). For solid phase extraction (SPE), Agilent Bond Elut PPL styrene-divinylbenzene polymer cartridges with 5 g of bed mass and 60 mL of column volume were used. Prior to loading, the cartridges were activated with 30 mL of methanol (MeOH) and then washed with 30 mL of H_2_O (pH 2, LCMS grade) and 30 mL of MeOH (LCMS grade), followed by equilibration with 30 mL of H_2_O (pH 2, LCMS grade). For sample loading, acidified seawater was drawn through a serological glass pipet connected to polypropylene tubing that split into 8 SPE PPL cartridges in parallel. We used a vacuum SPE station (Agilent 20 port SPE station) to maintain a flow rate of approximately 20 mL/min/cartridge and a total loading time of 20 h. A process blank was collected using 4 L of acidified H_2_O (pH2, LCMS grade) and a 5 g cartridge using the same SPE protocol as that above. After sample loading, the cartridges were desalted with 60 mL of H_2_O (pH 2, LCMS grade) and dried under N_2_ gas. After drying, the cartridges were eluted with 20 mL of MeOH per cartridge, resulting in a total of 160 mL of the sample extract. An internal standard (IS) mix was added, containing 5 μg of each of the following compounds in MeOH: domoic acid, kainic acid, isoxaben, irgarol, imazapyr, heroin, methamphetamine, and cocaine. Of the pooled sample, 150 mL were then aliquoted into 100 individual HPLC vials (1.5 mL each). The blank sample was eluted with 10 mL MeOH and aliquoted into 2 mL vials. All vials were dried down in a vacuum centrifuge overnight at room temperature. Six aliquots were weighed after the drying process, resulting in 1.8 mg of total sample dry mass per vial. Two representative aliquots were each redissolved in 2 L of H_2_O (pH 2) for total organic carbon (TOC) measurements, resulting in 30.0 μmol/L and 0.72 mg of C/vial. The overall extraction efficiency was 43.3%.

### Algae Extract Preparation

Lyophilized cells from *Synechococcus* sp. were purchased from Merck (Product number 491764). A 0.83 g portion of cell material was suspended in 50 mL of LCMS-grade MeOH in a MeOH-rinsed Falcon tube. The mixture was then sonicated for 5 min and centrifuged at 5000 RCF for 10 min. The supernatant was decanted to a new Falcon tube, dried under N_2_, and redissolved in Milli-Q water. This was sonicated and centrifuged, and the supernatant was transferred to a 250 mL bottle, diluted to 150 mL, and acidified with 150 μL of HCl. 8 mL of the solution was extracted on a preconditioned PPL cartridge (1 g; Agilent), and retained metabolites were eluted with MeOH and dried under N_2_. This extract was redissolved in 2 mL of MeOH, and 0.1 mL was taken for DOC analysis. This sample was dried under N_2_ and redissolved in 36.5 mL of Milli-Q water. The DOC concentration was 5.79 μg/mL after subtraction of the blank, indicating 2.1 mg/mL C in 2 mL of the stock solution. From the stock solution, three new stock solutions with 1800, 600, and 200 μg/mL were prepared in MeOH.

### Ring-Study Samples

The final sample preparation scheme for the ring study is shown in Figure 1. Samples were prepared from the dried DOM and algae extracts, as described in the following: the 60 dried DOM extracts from San Diego were redissolved in 100 μL of MeOH (Optima LCMS grade, Thermo Fisher) each, from which 50 μL were transferred into new 2 mL glass vials (Thermo Fisher, Level 3), resulting in a total of 120 DOM samples. To 28 of these vials, 25 μL of 1800 μg/mL algae extract was added (A45 M sample). To the next 28 vials, 25 μL of 600 μg/mL algae extract was added (A15 M sample). To the last 28 vials, 25 μL of a 200 μg/mL algae extract was added (A5M sample). Finally, 25 μL of 1800 μg/mL algae extract was added to 28 new vials and mixed with 50 μL of MeOH (A sample). For the blank samples, 4 blank aliquots were each redissolved in 100 μL MeOH, pooled, and 150 μL of the standard mix was added. 18 μL of the resulting solution was aliquoted into 28 new 2 mL vials. Finally, all vials were dried down in a vacuum centrifuge at 35 °C, capped, and shipped to the participating laboratories.

**Figure 1:**
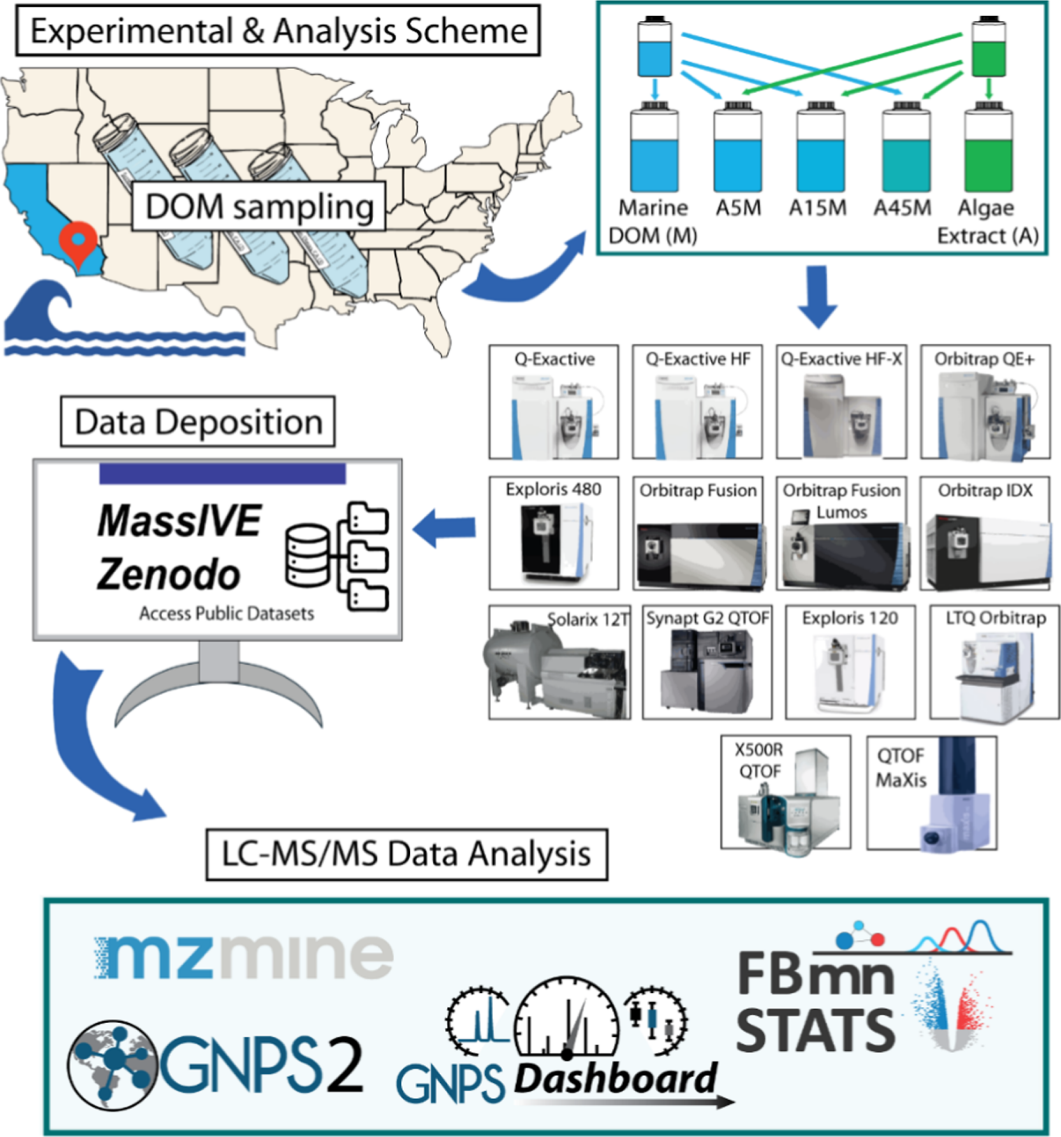
Experimental and analysis scheme. Dissolved organic matter (DOM) and algae extracts were prepared and mixed locally and sent out to the participating laboratories for data acquisition using standardized methodology. Acquired data was then deposited to the MassIVE repository and retrieved for data analysis using an open-source analysis pipeline. (Mzmine logo credit: mzio GmbH; GNPS2, GNPS dashboard, and FBmn STATS logo credit: Mingxun Wang & Daniel Petras).

### LC–MS/MS Analysis

For LC–MS/MS analyses, the samples were redissolved in 200 μL of 50% aq. MeOH (Optima LCMS grade, Thermo Fisher), and process blanks were redissolved in 100 μL of 50% aq. MeOH (Optima LCMS grade, Thermo Fisher). Final concentrations are displayed in Table S1, and authentic standard compounds are listed in Table S2. For each LC–MS analysis, 5–10 μL of the sample was injected.

Reliable comparisons across instruments require that chromatographic and MS acquisition settings be standardized as much as possible.^29^ Thus, for LC–MS/MS data acquisition, the following standardized parameters were prescribed for all participating laboratories. For LC separation, a default C18 UHPLC column with particle sizes <2 μm and column dimensions of 2 mm diameter and 10 or 15 cm length was recommended. A detailed overview of columns and LC methods is provided in Table S3. Mobile phases consisted of H_2_O (Optima LCMS grade, Thermo Fisher) (A) and acetonitrile (Optima LCMS grade, Thermo Fisher) (B), both containing 0.1% formic acid (FA, Optima LCMS grade, Thermo Fisher). Default flow rates of 0.4 mL/min and a linear two-step gradient starting at 5% B, followed by a linear gradient beginning at 0.5 min from 5 to 50% B until 7 min, followed by a second linear gradient to 99% B, until 10 min, were applied by most laboratories. MS/MS data was acquired in both positive and negative ionization modes using DDA. Detailed MS/MS settings from each laboratory are provided in Table S4.

### Data Analysis

The LC–MS/MS raw files were converted to. mzML format using msConvert (ProteoWizard)^30^ and uploaded to the MassIVE repository (https://massive.ucsd.edu/). The data was analyzed as individual data sets through CMN^31^ and FBMN^28^ in GNPS­(https://gnps.ucsd.edu) and as a unified global data set including all samples through both CMN and FBMN workflows on the GNPS2 platform (https://gnps2.org). Data preprocessing for FBMN, which takes into account MS1 extracted ion chromatograms for feature abundance measurements, was carried out with mzmine (ver. 4.2.0).^32^ CMN uses raw data directly, basing feature abundances on maximum precursor intensity, which does not necessarily correspond to peak apexes and thus is quantitatively less accurate than FBMN. In both cases, MS/MS spectra within the data set are compared to each other to create a molecular network in addition to spectral matching to public library spectra, providing structural annotations. Detailed settings and URLs of the processed molecular networking jobs are provided in the Supporting Information. Following FBMN and CMN data processing in GNPS2, downstream data cleanup included the removal of features that had occurred in the process blanks. We used a threshold whereby features that showed more than 30% intensity in blanks compared to samples were removed. This cutoff is typically user-defined and can be lowered or increased to adjust to the needs of a specific study (e.g., high or low levels of background contamination or desired robustness).^33^ Following blank removal, features with zero intensity were imputed, and normalization to total ion current (TIC) before Principal Coordinate Analysis (PCoA) and Permutational Multivariate Analysis of Variance (PERMANOVA) using the Bray–Curtis dissimilarity metric was performed with the FBMN Stats App (https://fbmn-statsguide.gnps2.org/).^33^ UpSet plots were generated with Intervene (https://asntech.shinyapps.io/intervene/).^34^

## Results and Discussion

### Assessment of LC–MS/MS Data and Methodological Aspects that Drive Data Set Suitability for Unified Analysis

Twenty-four laboratories completed the data acquisition and submitted their data to the public MassIVE data repository (https://massive.ucsd.edu/ProteoSAFe/), an overview of all data sets is provided in the supplemental csv table. During inspection of the data via the GNPS dashboard (https://dashboard.gnps2.org/),^35^ we visualized LC–MS/MS data as heatmaps with MS/MS events indicated as blue checkmarks (Figures 2A and S13).

**Figure 2:**
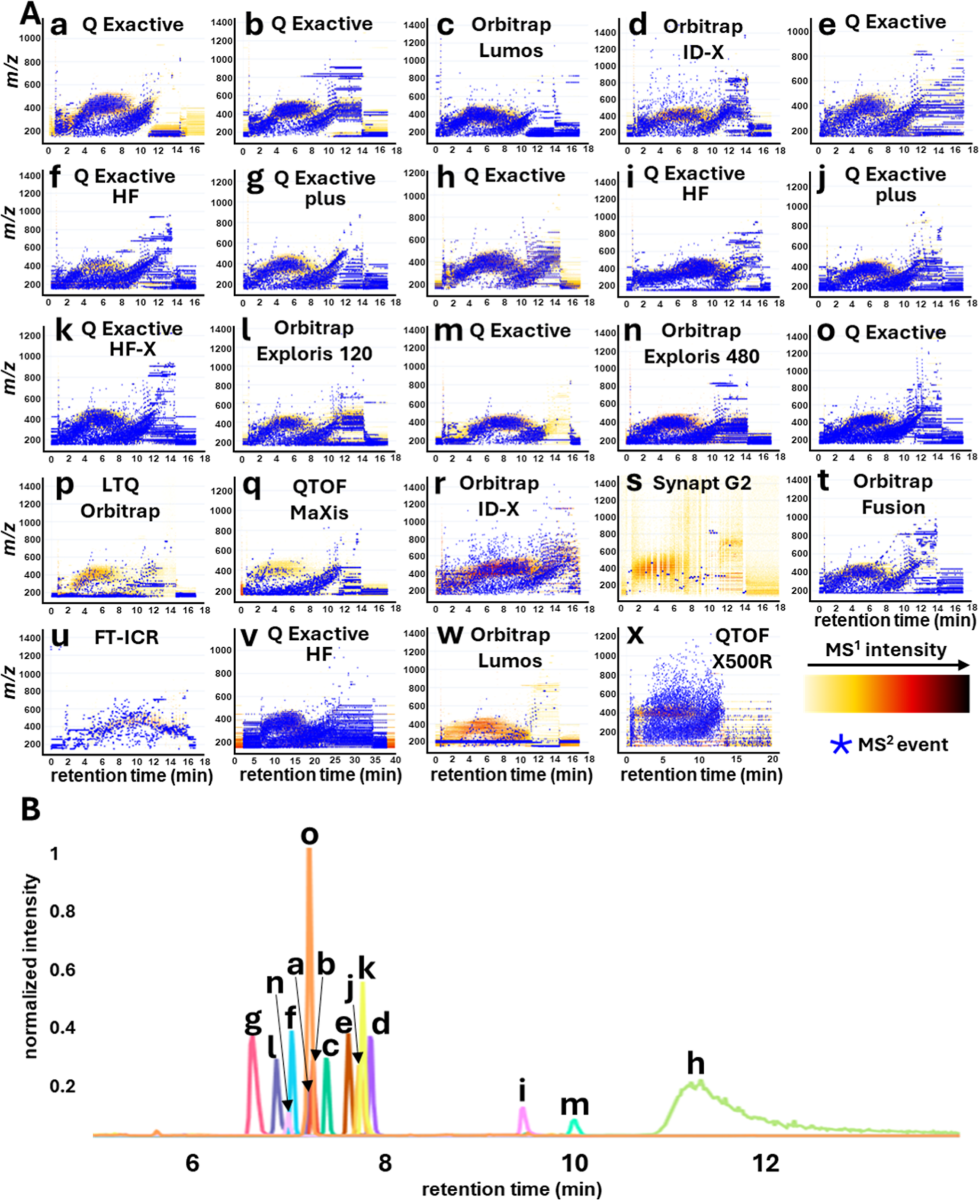
Overview of LC–MS/MS data. (A) LC–MS/MS (ESI+) heatmaps and MS/MS placement for a representative sample (A45M) for each analyzing laboratory. Data sets from laboratories (a–o) were selected for subsequent unified analysis. (B) Extracted ion chromatogram (XIC) of the internal standard irgarol for the selected Orbitrap data sets (ESI+) normalized to the highest peak.

Our criterion for inclusion in the unified data set for further in-depth analysis was that at least six out of eight internal standards had to be correctly matched to the respective GNPS library entries in positive ESI mode. As a first assessment of data amenability for unified analysis through FBMN, library ID matching of added IS was evaluated individually for each laboratory using CMN. Five laboratories matched all eight standards, seven laboratories matched seven standards, four laboratories matched six standards, and another eight laboratories matched less than six standards. Reasons for not matching standards may include poor ionization, insufficient mass accuracy, low scan rates, and/or inadequate DDA parameter selection, such as omission of dynamic exclusion or inconsistent setting of collision energies. In DDA experiments, low scan rates and long duty cycles reduce the number of MS/MS events acquired, which lowers the chance that internal standards are selected. Dynamic exclusion is designed to temporarily prevent redundant reacquisition of already fragmented precursor ions. If dynamic exclusion is not used, the instrument repeatedly targets the same intense background or matrix ions and “wastes” MS/MS events instead of sampling a broader set of precursors, potentially excluding spiked standards. Likewise, suboptimal collision energies can produce either poorly- or overfragmented spectra with low similarity to reference spectra, further decreasing the rate of successful standard identification. It is important to point out that CMN is an MS/MS-based data analysis strategy, mainly used for qualitative analysis. CMN uses spectral counts or precursor intensities for semiquantitative estimations, which are less sensitive and less accurate than MS1-based quantification approaches, such as FBMN.

The total number of library IDs matched within each data set is shown in Figure 3 and full tables, including those that were not selected for subsequent co-analyses, are shown in Table S5. For the ESI+ CMN data, these numbers range from 71 to 158 for the data sets selected for further coanalysis and from 0 to 107 for those that were not selected. The total number of clustered features (clusters) ranged from 1375 to 5061 for selected data sets, showing that the proportion of obtained spectra that were assigned to library IDs was low, averaging 3.8% across the selected data sets, which is comparable to other DOM LC–MS/MS data.^12,36,37^ Due to its sheer complexity, only a small fraction of features in DOM are selected for fragmentation during DDA. Taking into account this subsampling bias during DDA, the true annotation rate must be substantially lower than the stated average of 3.8%. This implies that only a small portion of the diversity that constitutes DOM can currently be linked to specific molecular structures. Notably, the number of library IDs did not scale with the number of MS/MS features across data sets (linear regression, *R* ^2^ = 0.04). This occurs because the more intense peaks in each data set were the ones matching library spectra. Acquiring MS/MS data from lower intensity features in a complex sample, such as DOM, will increase the number of spectra for previously unexplored compounds without spectral library entries. In comparison to the ESI+ data, the ESI- data generally yielded lower numbers of features and library matches, the latter being mainly driven by the limited amount of negative mode CID spectra in the spectral libraries used.

**Figure 3:**
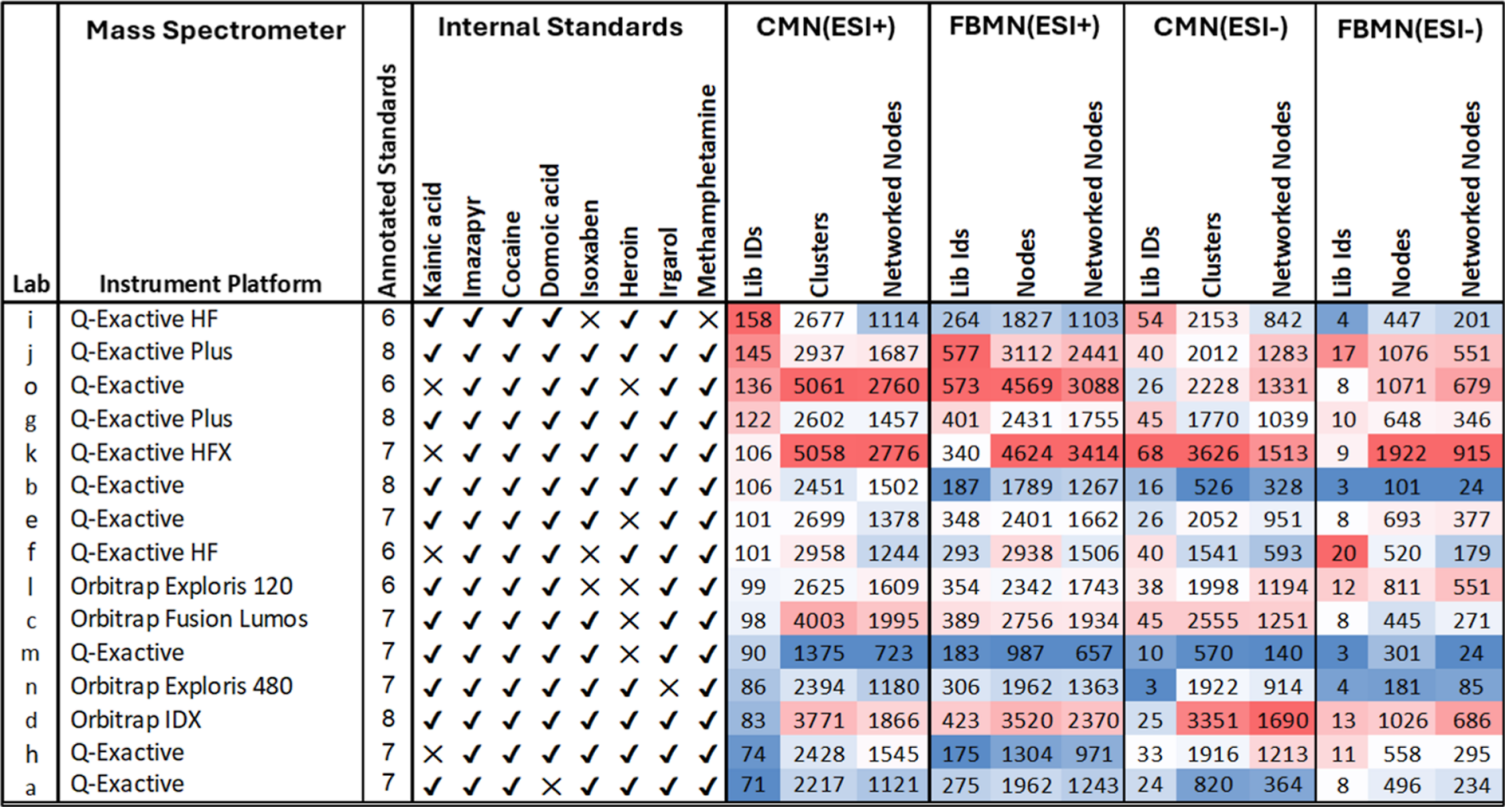
Summary of molecular networking metrics from selected data sets.

Other factors that can impact discrepancies between observed and “true” annotation rates are the presence of chimeric MS/MS spectra derived from coeluting isobaric precursors, as well as ion adducts and in-source fragments. In the first case, chimeric MS/MS spectra contain mixed fragment ions from more than one precursor, lowering the spectral purity and making them difficult to match to any single library spectrum. In the second case, multiple ion species (e.g., adducts and in-source fragments) are counted as separate features, even though they arise from the same underlying molecule, artificially inflating feature counts without increasing the number of distinct metabolites. We assessed the rate of chimeric spectra between the different laboratories using the mzmine precursor purity checker. As the precursor isolation width was constant at 1 *m*/*z* among the 15 aligned data sets, we observed a moderate differences in chimeric spectra rates, which average around 55.9%, ranging from 35.3%–77.0% (Figure S14A). Investigating the relationship between chromatographic peak height and precursor purity (Figure S14A), we observed a strong trend of increasing precursor purity for higher intensity features, which we attribute to highly abundant labile metabolites that stay on top of the refractory DOM background. Interestingly, those data sets that acquired fewer MS/MS spectra had higher average purity values (e.g., lab *m* and *n*), while laboratories with faster acquisition speed and deeper coverage contained more chimeric spectra. The only outlier was lab h, which showed the highest number of chimeric spectra, which we attribute to the lower chromatographic performance (see Figure 2B), likely due to the use of an HPLC column with 5 μm silica particles, in contrast to the sub-2 μm particle sizes employed by the other laboratories.

In this study, harmonized chromatography and DDA MS/MS settings were provided to all participating laboratories to achieve a high comparability. Nevertheless, six data sets (h, p, s, u, v, and w) were collected under diverging conditions due to limitations of certain instruments (e.g., low scan speed), user preferences (e.g., choice of chromatography system), and/or user errors (e.g., no dynamic exclusion). Consequently, these data sets contained low coverage of MS/MS events despite state-of-the-art instrumentation. In applications such as long-term environmental monitoring, regional surveys, or targeted ecosystem experiments, such incomplete MS/MS coverage could narrow the set of DOM components that can be reliably detected and interpreted and in turn limit the level of detail at which DOM composition and its variability can be resolved.

Metrics include the presence or absence (√/×) of internal standard annotation during ESI+ classical molecular networking (CMN), as well as the number of library ID matches, clustered features (clusters), networked nodes, singletons, and annotation rates obtained from both CMN and feature-based molecular networking (FBMN) of ESI+ and ESI- data. A full table including the results from all data sets is provided in the Supporting Information, Table S5. The color bar shows values ranging from low to high, which are calculated separately for each column of values.

Data sets that met the criterion of annotating 6 out of 8 standards (ESI+) showed a consistent pattern of tightly clustered MS/MS acquisition events from retention times of 3 to 10 min. Some of the excluded data sets (r, t, v, x) showed similar coverage but exhibited problems with either data conversion, inconsistent collision energy settings, insufficient mass accuracy, substantial retention time shifts compared to the other laboratories, or poor peak quality. For laboratory q, it appears that the DDA settings were not adequate to capture MS/MS spectra of the characteristic *m*/*z* 400–600 ions, with MS/MS acquisition events focused on lower mass ions, which produced greater MS1 ion intensities for this instrument and dominated DDA precursor selection.

Laboratory w, which used a more recent instrument (Orbitrap Fusion Lumos), obtained excellent coverage of DOM material at the MS1 level but poor MS/MS coverage. In their case, dynamic exclusion was not activated, which caused repeated MS/MS acquisition around *m*/*z* 200. Laboratories p and s exhibited very poor MS/MS coverage, attributable to inadequate DDA settings. The data from laboratory u were found to contain only a few MS/MS spectra covering the acquisition time range only sparsely because of the lower scan speed of the instrument.

When comparing retention times for the selected data sets through the spiked standards (Figures 2B and S1–S8), moderate retention time differences (<1 min from center) were observed for the majority of laboratories, while for laboratories h, i, and m, larger retention time differences (3–4 min from center) were observed, which were attributable to deviations in the chromatographic gradient used. Laboratory h additionally showed broader chromatographic peaks, likely due to column choice (5 μm particle size compared to sub-2 μm for the other laboratories). The focus for acquiring comparable LC–MS/MS data should thus lie on standardized DDA (or DIA) MS/MS settings, as well as comparable chromatography columns and gradients.

### Feature Abundance Distribution across Laboratories

Following the initial data quality assessment, laboratories with at least six out of eight matched internal standards were selected for further analysis. To assess interlaboratory consistency and comparability, both CMN and FBMN methods were applied across the selected data sets. This approach also aimed at assessing whether MS1 and RT (FBMN) or MS/MS (CMN) alignment would better support data set comparability.^38^ FBMN provides a more accurate assessment of signal abundance trends but with a trade-off of risk of false alignment since no MS/MS matching is performed between separate samples, whereas CMN relies on matching of MS/MS signals, which can be negatively impacted by chimeric spectra and DDA subsampling bias. Furthermore, CMN provides less accurate quantitative information, as precursor intensities vary when MS/MS acquisition is triggered at different points in a chromatographic (MS1) peak. Therefore, when using CMN for data alignment, interlaboratory variability is confounded with variability introduced due to DDA subsampling bias and the imprecise precursor intensity during MS/MS triggering.

For FBMN analysis, an RT tolerance of 1.7 min was applied based on an inspection of RT deviations for the spiked standards, which were generally within this window except for laboratories h, i, and m (Figure 2B).

To evaluate the reproducibility of feature detection, we assessed the extent of feature overlap across the laboratories. When all features were considered, the presence/absence analysis showed that features detected in all laboratories were in the vast minority (1.0% of the features are shared between all laboratories in positive mode FBMN and 2.1% in CMN; 1.8% in negative mode FBMN and 0.2% in CMN (Figures 4A,S15A–S17A). In the ‘top 1000’ highest intensity features, 4.7% are shared across all laboratories for FBMN and 3.0% for CMN (Figures 4B and S16B, for negative mode, 4.3% in FBMN and 1% in CMN Figures S16B and S17B). Laboratories share only a small core set of strong, high-intensity signals, whereas the majority of weaker features are detected inconsistently and behave more like lab-specific noise when data sets are compared.

**Figure 4:**
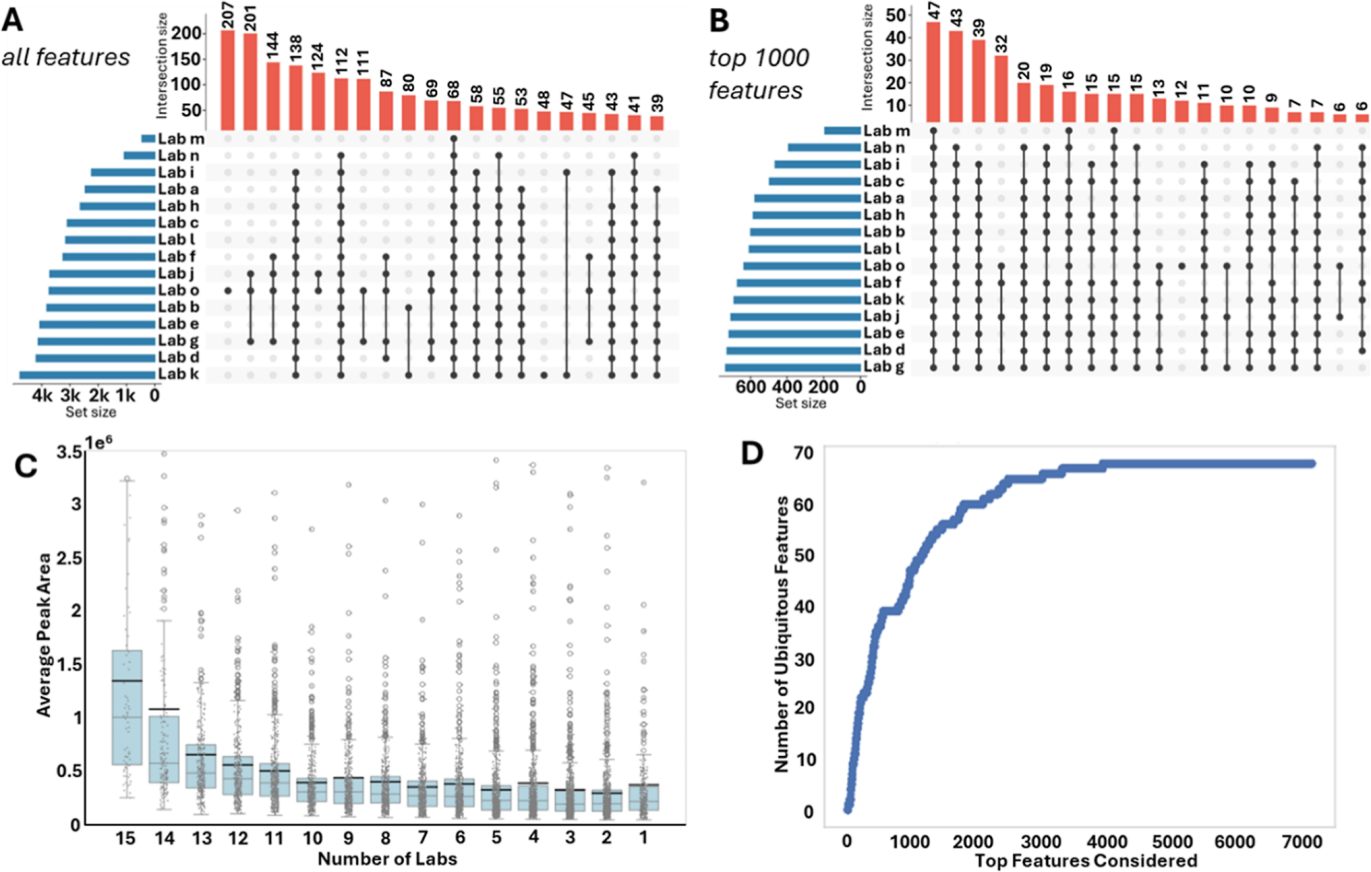
Overview of shared and ubiquitous LC–MS/MS features across laboratories. LC–MS/MS (ESI+) data was analyzed with Feature-Based Molecular Networking (FBMN). (A) UpSet plot illustrating the distribution of shared features across laboratories when considering all detected features. (B) UpSet plot showing shared feature distributions across laboratories, restricted to the top 1000 most intense features (mean across samples). (C) Boxplot showing mean peak area as a function of the number of laboratories in which a feature is observed. (D) Cumulative count of features detected in all laboratories, plotted against the feature mean intensity rank of features considered. Both plots (C,D) indicated that most features shared between all data sets are also among the highest intensity features.

Features that were shared between all laboratories were of higher average intensity for FBMN, with a trend toward lower average intensity for ‘rarer’ features (Figures 4C and S16C). For CMN, this trend was not readily observed, and the higher average intensities were observed from unique features, especially for the positive mode data (Figures S15C and S17C).

To further examine how feature intensity relates to how widely a feature is observed across laboratories, we carried out a rank-based ubiquity evaluation. In this analysis, we first ordered all features by their mean peak area across all samples (from the highest to lowest intensity). We then progressively included features along this ranked list and at each step counted how many of the included features were present in all laboratories. The resulting cumulative curve shows that, for FBMN, the number of ubiquitous features rises steeply and reaches a plateau after relatively few high-intensity features have been considered, indicating that most shared features are among the most abundant ones (Figure 4D). For CMN, this increase follows a more linear pattern (Figure S15D), suggesting that ubiquitous features are more evenly distributed across the intensity range, and similar observations were made for FBMN and CMN of the negative mode data (Figures S16D and S17D). For CMN, feature finding is solely based on the presence of MS/MS spectra and not whether these originate from actual chromatographic peaks as considered in FBMN. Thus, background signals, e.g., originating from the sample matrix, are much more likely to result in CMN features, as opposed to FBMN. Moreover, CMN does not consider RTs, leading to the risk of merging isobaric features with highly similar MS/MS spectra but different structures. In addition, identical structures detected across laboratories can be clustered into separate features based on slight to moderate differences in fragmentation spectra elicited by spectral chimerism or slight differences in instrument performance. FBMN, on the other hand, is agnostic to differences in MS/MS spectra since feature alignment is based on MS1 *m*/*z* and RT of features. This makes it more robust but also introduces the risk of false alignment of features. Therefore, limiting the analysis to the highest-intensity features results in a higher percentage of feature overlap across laboratories, likely because higher-intensity features are more likely to be sampled during DDA. In addition to subsetting the analysis to high-intensity features, increasing overall DDA MS/MS events, for example, through repetitive analysis, iterative DDA (e.g., AcquireX), or 2D-LC–MS/MS approaches, might mitigate these effects and increase the depth of features that can effectively be aligned.

### Multivariate Statistical Analysis Distinguishes Sample Types between All Data Sets

After inspecting the overall distribution of feature intensities across laboratory data sets, we assessed whether the different laboratories reached similar qualitative conclusions in distinguishing the different sample types. First, PCoAs (based on Bray–Curtis dissimilarity) were performed separately for each laboratory using both FBMN and CMN, revealing clear clustering by sample type and consistent trends within each laboratory for the positive (Figures S18 and S20) and negative mode data (Figures S19 and S21).

Since individual laboratory analyses successfully distinguished the sample types and demonstrated comparable PCoA patterns, the next step was to assess whether this differentiation was observed when all selected data sets were analyzed collectively. Figure 5 shows the PCoA results for the FBMN analysis, indicating a trend toward distinguishing sample types, particularly for samples A and A45M, which are the endmembers of the sample mixtures. However, aside from this tendency for separation, the laboratory in which data was acquired was the primary driver of clustering according to the PERMANOVAs (Figure 5A; *R* ^2^ = 0.25 for the sample type and *R* ^2^ = 0.51 for laboratory).

**Figure 5:**
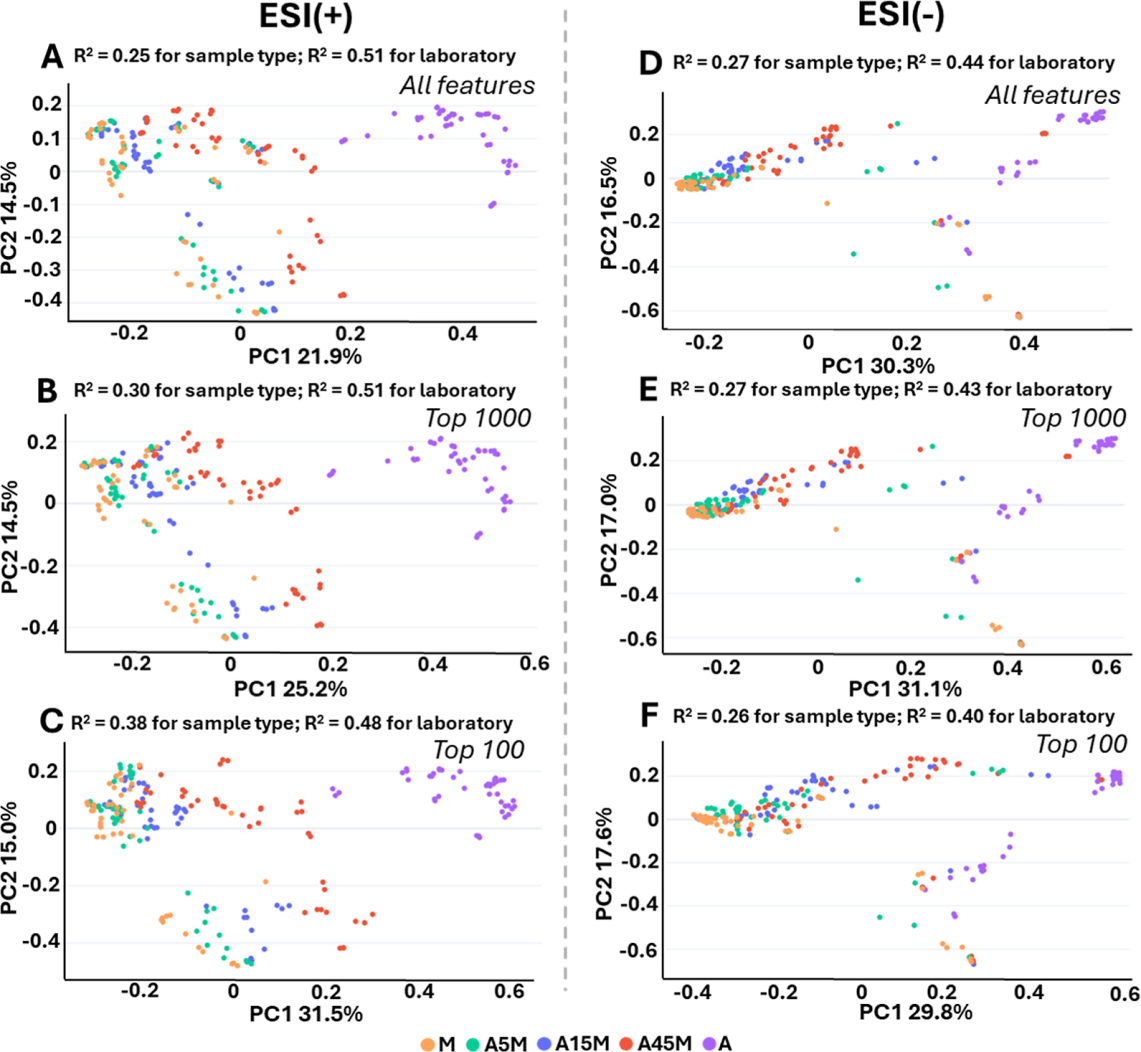
Distinction of sample types in PCoA increases when restricting the data frame to high-intensity features. (A) PCoA and PERMANOVA using Bray–Curtis dissimilarity for the Feature-Based Molecular Networking analysis (FBMN, ESI+); (B) for the FBMN (ESI+) analysis considering only the top 1000 most intense features (by average feature sum); (C) for the FBMN (ESI+) analysis considering the top 100 features. (D) PCoA and PERMANOVA using Bray–Curtis dissimilarity for the Feature-Based Molecular Networking analysis (FBMN, ESI-); (E) for the FBMN (ESI-) analysis considering only the top 1000 most intense features (by average feature sum); (F) for the FBMN (ESI-) analysis considering the top 100 features. The colors indicate the different extracts.

A key question in interpreting these findings is whether the tendency observed in PCoA across all laboratories is influenced by variations in the detection of low-abundance features, considering that the overlap between laboratories was related to “top” features (see previous section). Thus, a subsequent PCoA focusing on the top 1000 features (by mean peak area across samples with only nonzero entries considered) was conducted, revealing a clearer separation by sample type (Figure 5B; *R* ^2^ = 0.30 for sample type and *R* ^2^ = 0.51 for laboratory). A stronger trend was observed when narrowing the focus to the top 100 features (Figure 5C; *R* ^2^ = 0.38 for sample type and *R* ^2^ = 0.48 for laboratory). This further indicates that high-intensity features contribute most toward distinguishing sample types, regardless of laboratory. Next, we carried out the same analysis for the CMN results (Figure S22), yielding equivalent results, although this effect is more pronounced for FBMN, likely due to the higher sensitivity of MS1-based alignment to correctly assemble relative feature intensities.

Interestingly, these trends are not readily observed for the negative mode data (Figure 5D–F). This is likely due to the widespread detection of recalcitrant DOM components that are present at very low concentrations in the DOM material, thus yielding many low-intensity features characteristic of the sample types. In this interlaboratory context, these patterns imply that multivariate comparisons aimed at separating sample groups tend to be more robust when they are driven by the shared, high-intensity subset of DOM features.

The relatively modest *R* ^2^ values reflect how DOM complexity, instrument-specific DDA behavior, and current limitations in cross-laboratory alignment redistribute biological variation into laboratory-specific axes of variance, rather than an absence of underlying ecological signal. DOM is an extremely complex mixture, and DDA captures only a small, intensity-biased subset of ions, with differences in scan speed, duty cycle, ion suppression, and in-source fragmentation across instruments leading to heterogeneous MS/MS coverage across laboratories, especially for low-intensity features. These inconsistencies, combined with imperfect cross-lab feature alignment in FBMN and CMN due to retention-time drift, peak-shape differences, and chimeric or instrument-specific MS/MS spectra, introduce laboratory-specific variance into the merged feature table. Consequently, part of the underlying biological trend is dispersed across technical variation, reducing the apparent explanatory power of sample type in the PERMANOVA, even though the major qualitative trends remain conserved. Similar patterns of strong laboratory or platform effects have been reported in previous interlaboratory comparisons of DOM HR–MS and non-targeted MS data sets.^39,40^

Focusing more closely on the sample gradient, sample type A, which represents the least complex sample (algal extract only, compared to mixtures of algal extract and/or complex DOM extract), clearly showed a much greater distance from the other samples than the distance observed between A45M, A15M, A5M, and M, which is not surprising when considering the sample mixing gradient (Table S1). Accordingly, when sample A is excluded, the PCoAs (Figure S23, ESI+) indicate that laboratory differences, rather than sample type, are much more predominant in driving separation for both FBMN and CMN (FBMN: *R* ^2^ = 0.062 for the sample type and *R* ^2^ = 0.76 for laboratory; CMN: *R* ^2^ = 0.073 for sample type and *R* ^2^ = 0.72 for laboratory). Although the sample effect is more pronounced when high-intensity peaks are evaluated, as previously noted, the laboratory effect remains overall more significant in both approaches. However, analysis of the individual PCoAs (Figures S24 and S25) reveals that each laboratory can still differentiate between the sample types.

Taken together, even with our simplistic alignment approaches, we achieved a clear differentiation of overall sample composition between the different data sets. At the same time, the shared trends in feature overlap and PCoA profiles highlight an important aspect of DDA applications in non-targeted LC–MS/MS analysis: while individual laboratories may not detect exactly the same ensemble of features, especially for less abundant compounds, they can still achieve similar qualitative conclusions in terms of group differentiation,^40^ which is often one of the goals in non-targeted metabolomics studies. Interestingly, the qualitative interpretation of metabolomics data may also remain consistent within large ranges of the signal threshold selected for including features (detected ions) in the data set.^41^ This is especially encouraging for environmental research questions where samples from different conditions, such as before versus after a disturbance, along a pollution gradient, or across contrasting ecosystems, are compared.

### Sample-to-Sample Similarity is Driven by the Same Metabolites across Data Sets

Since all selected data sets were adequate for distinguishing the different sample types, we next investigated whether the molecular features driving these distinctions were conserved across laboratories. For this, we filtered the complete feature table to only retain features that returned level 2 library IDs,^42^ representing putatively annotated compounds based on GNPS2 spectral library matches.

To mitigate shortcomings in data set alignment, we summed the peak areas of features that shared identical library IDs. This approach addresses cases where the 1.7 min RT window used in FBMN was too narrow for data sets with larger RT variation and cases where MS/MS spectra were too dissimilar to be clustered in CMN but still produced identical library matches. We applied this procedure to both the FBMN and CMN analyses, which greatly enhanced the overlap of important features for sample classification between data sets (compare Figures 6A and S26). It is important to point out that while merging features by metabolite ID improves cross-data set comparability, the merging might also collapse true structurally resolved molecules such as regioisomers or stereoisomers. The change in accuracy of the random forest models due to the merging can be expected to be low, as the merged features make up only a small portion of all the features considered.

**Figure 6:**
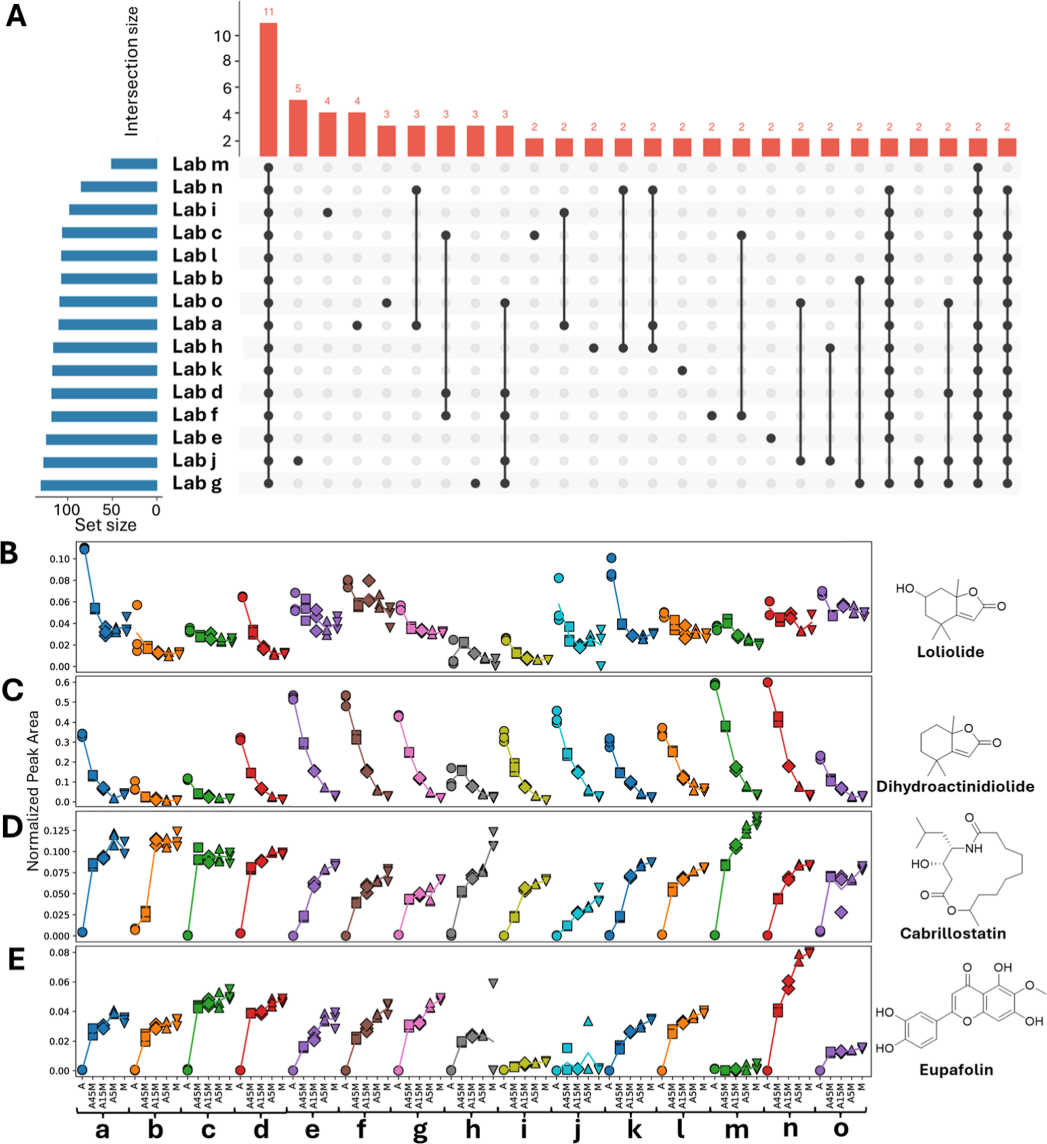
Random forest (RF) analysis of features driving sample type clustering. (A) UpSet plot showing the increased overlap (presence/absence) of significant features identified by RF analysis across laboratories using feature-based molecular networking of the positive mode ESI data (FBMN) and merged features based on identical spectral library annotation. (B–E) Relative abundances (sample-centric normalization) of selected top 10 features in each laboratory contributing to sample-type classification based on RF analysis using FBMN. The features marked as predictors for sample type include features annotated as loliolide and dihydroactinidiolide, for which relative abundances decrease with increasing algal compared to DOM concentration, and features such as cabrillostatin and eupafolin, for which relative abundances increase under the same conditions.

Next, the resulting merged feature table was split according to laboratory, and TIC-normalized subsets of data were individually subjected to Random Forest (RF) analysis through the FBMN Stats App.^33^ RF is a supervised classification algorithm that combines decision trees trained on a subset of preclassified samples to predict sample classes and to quantify the importance of each input variable.^43^ Here, we used RF to evaluate how accurately sample types could be predicted from metabolite profiles and to rank metabolites by their importance for correct classification, identifying compounds that most strongly drive differences in the DOM composition between sample classes. This analysis yielded sample type prediction accuracies between 80% and 100%. We then examined the features that were important for classification within each laboratory and compared the overlap of these features across laboratories (Figure 6A). In the FBMN results, the top 5% of driving features prioritized across data sets by the RF analysis formed the largest intersection in the UpSet plot. For CMN, this corresponded to only 1% of driving features (*n* = 3, Figure S27), suggesting that, between the two workflows, FBMN is more robust at identifying ecologically relevant molecules due to the higher fidelity of captured signal intensities.

For the FBMN analysis, we next asked which annotated molecules were consistent drivers for distinguishing sample types across laboratories. To do this, we took the RF importance scores for each feature from all 15 laboratory-specific models, averaged them, and then ranked features by this mean importance. Using this rank, the seven molecules with the highest mean importance appeared among the important predictors in every laboratory, and the remaining three molecules in the top 10 were important in 13 or 14 laboratories. We then visualized normalized abundances for selected top-importance features with high-quality spectral matches (Figure 6B–E; the top 60 predictor features are listed in Table S6).

Features annotated as loliolide and dihydroactinidiolide decreased with the reduced proportion of algal extract in the samples, whereas features annotated as cabrillostatin and eupafolin showed the opposite trend with higher intensities at higher marine DOM proportions.

These results are in agreement with the nature of the samples. Loliolide is a natural product known to be present in various algae^44^ and dihydroactinidiolide is a known metabolite of *Synechococcus*.^45^ Cabrillostatin is a metabolite that was recently discovered in seagrass meadows from the Southern California coast.^46^ Similarly, eupafolin has been described to be produced by Pacific Seagrass,^47^ providing a rationale for the presence of these metabolites in our (Pacific) DOM extract. The consistent detection of these compounds across laboratories indicates that the molecules driving sample separation can be interpreted as biologically meaningful markers of algal production and seagrass-meadow-derived coastal DOM.

### Recommendations for Method Standardization, Quality Control, and Data Sharing to Achieve Interlaboratory Data Comparability

Our interlaboratory comparison highlights the critical importance of method standardization for robust and reproducible non-targeted LC–MS/MS analysis. To ensure meaningful comparisons across laboratories, harmonization of both chromatographic separation and mass spectrometric acquisition parameters will be essential. While we recognize that not all laboratories have access to identical instrumentation, we strongly recommend the use of similar instrument classes with similar scan speed, sensitivity, resolution, and MS/MS settings for time series and larger surveys. Furthermore, the adoption of community-wide quality control standards, such as minimum requirements for the detection of known metabolites from internal standards and/or common reference materials,^48^ will be important for evaluating whether data sets are comparable.

Without such standardization, long-term or multibasin data sets may confound true biological changes in DOM composition with variation introduced by instrumentation or methodological differences. In addition to method standardization, the development of machine learning-based retention time alignment, improved feature matching, and spectral deconvolution workflows tailored for the integration of multilaboratory data sets would greatly enhance the ability to reduce nonbiological variability and make results more consistent.

Our analysis demonstrates that when core criteria are met, DOM samples yield broadly comparable molecular profiles across laboratories. In this way, this study also served as a test case to evaluate the user’s influence in each laboratory to interpret and implement the provided instructions accurately, pointing to the importance of clear communication and highlighting the practical challenges of achieving consistent method implementation across laboratories.

A fundamentally important aspect, in addition to harmonized data acquisition, is open data sharing of raw and processed data, standardization of data preprocessing (e.g., mass calibration and peak picking) and data formats (e.g., .mzML), as well as the contextualization of the data with metadata.^49–51^ Adhering to common data formats and providing rich environmental metadata (e.g., location, depth, physicochemical parameters, and sampling context) enables the reuse of LC–MS/MS DOM data sets in cross-study meta-analyses and is consistent with emerging best practices in metabolomics repositories and metadata standards.^52^ Together, these practices will be crucial for building comparably acquired, well-documented DOM data sets that can underpin future basin-scale surveys and long-term environmental monitoring programs.

### Implications

Analyzing DOM using LC–MS/MS methods remains challenging, and current methodologies are only beginning to address the complexity of these highly heterogeneous sample types. Standardizing the analytical workflow, including extraction, sample analysis, instrument parameters, and ideally the use of shared quality control samples, is essential for reliable interlaboratory comparison. Multivariate analysis provides a viable framework for comparing DOM data across laboratories, particularly when focusing on the most abundant features or highly distinctive sample characteristics.

Across laboratories, we found that different sample types could be distinguished; however, the specific features responsible for this separation were not conserved when data sets were aligned using current methods. The overlap of shared features improved substantially when the aligned data set was restricted to high-intensity features. Furthermore, when features were merged according to their chemical identity, interlaboratory overlap in the key features driving sample distinction increased markedly.

Despite these improvements, the generally low overlap in features, especially before merging by chemical identity, indicates that optimized workflows for the meta-analysis of diverse sample sets are still needed, even when similar instrumentation and standardized protocols are used. In addition to establishing standardized protocols, efficient alignment, normalization, and emerging retention time and batch correction tools could further improve the comparability of LC–MS/MS data between different experiments, laboratories, and instrument platforms. This especially becomes crucial when global surveys or time series should be compared, with data originating from multiple laboratories and consortia (e.g., TREC, GEOTRACES, and BioGeoSCAPES^53,54^ ).

## Supplementary Material



## Data Availability

All raw and processed MS data are publicly available through the Massive (massive.ucsd.edu) with the following ID: MSV000090156 as well as the GNPS2 (gnps2.org) with the following urls. FBMN (ESI+): https://gnps2.org/status?task=61e48c4af96944aeba73f59f0dbd51c2CMN (ESI+): https://gnps2.org/status?task=cc2c071be92f428ca85188ae3654ea32 FBMN (ESI-): https://gnps2.org/status?task=88f8fdb9665b42688b53cf560f4e23fd CMN (ESI-): https://gnps2.org/status?task=b8ad0450cf134dca9932394f856125f1 A detailed overview of individual data sets, accession numbers, and URLs for the processed data is available in the supplemental. csv table. Processed and source files, as well as python scripts, can be accessed at Zenodo 10.5281/zenodo.16897529.

## Notes

Additional information regarding methods and results of all participating laboratories, including molecular networking results, extracted ion chromatograms, multivariate statistical results, detailed listing of parameters used for classical and FBMN, and mzmine processing (PDF)

## References

[1] Hotchkiss, E. R. ; DelSontro, T. Organic Carbon Cycling and Ecosystem Metabolism. In Wetzel’s Limnology; Elsevier, 2024; pp 939–997.

[2] Demars B. O. L., Kemp J. L., Marteau B., Friberg N., Thornton B. (2021). Stream Macroinvertebrates
and Carbon Cycling in Tangled Food Webs. Ecosystems.

[3] Leyva D., Jaffé R., Courson J., Kominoski J. S., Tariq M. U., Saeed F., Fernandez-Lima F. (2022). Molecular
Level Characterization of DOM along a Freshwater-to-Estuarine Coastal
Gradient in the Florida Everglades. Aquat. Sci..

[4] Ward, N. D. ; Bianchi, T. S. ; Medeiros, P. M. ; Seidel, M. ; Richey, J. E. ; Keil, R. G. ; Sawakuchi, H. O. Where Carbon Goes When Water Flows: Carbon Cycling across the Aquatic Continuum. Front. Mar. Sci. 2017, 4. 10.3389/fmars.2017.00007.

[5] Sengupta A., Stegen J. C., Bond-Lamberty B., Rivas-Ubach A., Zheng J., Handakumbura P. P., Norris C., Peterson M. J., Yabusaki S. B., Bailey V. L., Ward N. D. (2021). Antecedent Conditions
Determine the Biogeochemical Response of Coastal Soils to Seawater
Exposure. Soil Biol. Biochem..

[6] Simon C., Roth V.-N., Dittmar T., Gleixner G. (2018). Molecular
Signals of
Heterogeneous Terrestrial Environments Identified in Dissolved Organic
Matter: A Comparative Analysis of Orbitrap and Ion Cyclotron Resonance
Mass Spectrometers. Front. Earth Sci..

[7] Morrison E. S., Liu Y., Rivas-Ubach A., Amaral J. H. F., Shields M., Osborne T. Z., Chu R., Ward N., Bianchi T. S. (2023). Mangrove Peat and Algae Leachates
Elicit Rapid and Contrasting Molecular and Microbial Responses in
Coastal Waters. Commun. Earth Environ.

[8] Heal K. R., Qin W., Ribalet F., Bertagnolli A. D., Coyote-Maestas W., Hmelo L. R., Moffett J. W., Devol A. H., Armbrust E. V., Stahl D. A., Ingalls A. E. (2017). Two Distinct
Pools of B12 Analogs
Reveal Community Interdependencies in the Ocean. Proc. Natl. Acad. Sci. U. S. A..

[9] Petras D., Koester I., Da Silva R., Stephens B. M., Haas A. F., Nelson C. E., Kelly L. W., Aluwihare L. I., Dorrestein P. C. (2017). High-Resolution Liquid Chromatography Tandem Mass Spectrometry
Enables Large Scale Molecular Characterization of Dissolved Organic
Matter. Front. Mar. Sci..

[10] Hawkes J. A., Patriarca C., Sjöberg P. J., Tranvik L. J., Bergquist J. (2018). Extreme Isomeric
Complexity of Dissolved Organic Matter Found across Aquatic Environments. Limnol. Oceanogr. Lett..

[11] Kalinski J.-C. J., Noundou X. S., Petras D., Matcher G. F., Polyzois A., Aron A. T., Gentry E. C., Bornman T. G., Adams J. B., Dorrington R. A. (2024). Urban and Agricultural Influences on the Coastal Dissolved
Organic Matter Pool in the Algoa Bay Estuaries. Chemosphere.

[12] Papadopoulos
Lambidis S., Schramm T., Steuer-Lodd K., Farrell S., Stincone P., Schmid R., Koester I., Torres R., Dittmar T., Aluwihare L. (2024). Two-Dimensional Liquid Chromatography Tandem Mass Spectrometry Untangles
the Deep Metabolome of Marine Dissolved Organic Matter. Environ. Sci. Technol..

[13] Longnecker K., Kido Soule M. C., Swarr G. J., Parsons R. J., Liu S., Johnson W. M., Widner B., Curry R., Carlson C. A., Kujawinski E. B. (2024). Seasonal and Daily Patterns in Known Dissolved Metabolites
in the Northwestern Sargasso Sea. Limnol. Oceanogr..

[14] Schramm T., Kalinski J.-C. J., Arini G. S., Da Silva R. R., Petras D. (2025). Uncovering
the Structural Space of Marine Dissolved Organic Matter. Ann. Rev. Mar. Sci.

[15] Aguilar-Alarcón P., Gonzalez S. V., Mikkelsen Ø., Asimakopoulos A. G. (2024). Molecular
Formula Assignment of Dissolved Organic Matter by Ultra-Performance
Liquid Chromatography Quadrupole Time-of-Flight Mass Spectrometry
Using Two Non-Targeted Data Processing Approaches: A Case Study from
Recirculating Aquaculture Systems. Anal. Chim.
Acta.

[16] Chu J., Liao Z. (2024). Optical and Molecular
Characteristics of Urban Wastewater Dissolved
Organic Matter: Insights into Their Correlations. Environ. Sci.:Water Res. Technol..

[17] Simon C., Dührkop K., Petras D., Roth V.-N., Böcker S., Dorrestein P. C., Gleixner G. (2022). Mass Difference Matching Unfolds
Hidden Molecular Structures of Dissolved Organic Matter. Environ. Sci. Technol..

[18] Hawkes J. A. (2024). Electrospray
Ionisation Suppression in Aquatic Dissolved Organic Matter Studies–Investigation
via Liquid Chromatography–Mass Spectrometry. Org. Geochem..

[19] Ruan M., Wu F., Sun F., Song F., Li T., He C., Jiang J. (2023). Molecular-Level
Exploration of Properties of Dissolved Organic Matter
in Natural and Engineered Water Systems: A Critical Review of FTICR-MS
Application. Crit. Rev. Environ. Sci. Technol..

[20] Patriarca C., Bergquist J., Sjoberg P. J., Tranvik L., Hawkes J. A. (2018). Online
HPLC-ESI-HRMS Method for the Analysis and Comparison of Different
Dissolved Organic Matter Samples. Environ. Sci.
Technol..

[21] Boiteau R. M., Corilo Y. E., Kew W. R., Dewey C., Alvarez
Rodriguez M. C., Carlson C. A., Conway T. M. (2024). Relating Molecular
Properties to the Persistence of Marine Dissolved Organic Matter with
Liquid Chromatography–Ultrahigh-Resolution Mass Spectrometry. Environ. Sci. Technol..

[22] Rodrigues
Matos R., Jennings E. K., Kaesler J., Reemtsma T., Koch B. P., Lechtenfeld O. J. (2024). Post Column Infusion of an Internal
Standard into LC-FT-ICR MS Enables Semi-Quantitative Comparison of
Dissolved Organic Matter in Original Samples. Analyst.

[23] Patrone J., Vila-Costa M., Dachs J., Papazian S., Gago-Ferrero P., Gil-Solsona R. (2024). Enhancing Molecular Characterization of Dissolved Organic
Matter by Integrative Direct Infusion and Liquid Chromatography Nontargeted
Workflows. Environ. Sci. Technol..

[24] Stincone P., Pakkir Shah A. K., Schmid R., Graves L. G., Lambidis S. P., Torres R. R., Xia S.-N., Minda V., Aron A. T., Wang M., Hughes C. C., Petras D. (2023). Evaluation of Data-Dependent
MS/MS Acquisition Parameters for Non-Targeted Metabolomics and Molecular
Networking of Environmental Samples: Focus on the Q Exactive Platform. Anal. Chem..

[25] Aigensberger M., Bueschl C., Castillo-Lopez E., Ricci S., Rivera-Chacon R., Zebeli Q., Berthiller F., Schwartz-Zimmermann H. E. (2025). Modular
Comparison of Untargeted Metabolomics Processing Steps. Anal. Chim. Acta.

[26] Martin J.-C., Maillot M., Mazerolles G., Verdu A., Lyan B., Migné C., Defoort C., Canlet C., Junot C., Guillou C. (2015). Can We Trust Untargeted Metabolomics? Results of the
Metabo-Ring Initiative, a Large-Scale, Multi-Instrument Inter-Laboratory
Study. Metabolomics.

[27] Hawkes J. A., d’Andrilli J., Agar J. N., Barrow M. P., Berg S. M., Catalán N., Chen H., Chu R. K., Cole R. B., Dittmar T. (2020). An International Laboratory Comparison of Dissolved
Organic Matter Composition by High Resolution Mass Spectrometry: Are
We Getting the Same Answer?. Limnol. Oceanogr.:Methods.

[28] Nothias L.-F., Petras D., Schmid R., Dührkop K., Rainer J., Sarvepalli A., Protsyuk I., Ernst M., Tsugawa H., Fleischauer M., Aicheler F., Aksenov A. A., Alka O., Allard P.-M., Barsch A., Cachet X., Caraballo-Rodriguez A. M., Da Silva R. R., Dang T., Garg N., Gauglitz J. M., Gurevich A., Isaac G., Jarmusch A. K., Kameník Z., Kang K. B., Kessler N., Koester I., Korf A., Le Gouellec A., Ludwig M., Martin H. C., McCall L.-I., McSayles J., Meyer S. W., Mohimani H., Morsy M., Moyne O., Neumann S., Neuweger H., Nguyen N. H., Nothias-Esposito M., Paolini J., Phelan V. V., Pluskal T., Quinn R. A., Rogers S., Shrestha B., Tripathi A., van der
Hooft J. J. J., Vargas F., Weldon K. C., Witting M., Yang H., Zhang Z., Zubeil F., Kohlbacher O., Böcker S., Alexandrov T., Bandeira N., Wang M., Dorrestein P. C. (2020). Feature-Based Molecular Networking in the GNPS Analysis
Environment. Nat. Methods.

[29] Lin Y., Caldwell G. W., Li Y., Lang W., Masucci J. (2020). Inter-Laboratory
Reproducibility of an Untargeted Metabolomics GC–MS Assay for
Analysis of Human Plasma. Sci. Rep.

[30] Adusumilli, R. ; Mallick, P. Data Conversion with ProteoWizard msConvert. In Proteomics: methods and protocols; Comai, L. , Katz, J. E. , Mallick, P. , Eds.; Springer New York: New York, NY, 2017; Vol. 1550, pp 339–368. 10.1007/978-1-4939-6747-6_23 28188540

[31] Aron A. T., Gentry E. C., McPhail K. L., Nothias L.-F., Nothias-Esposito M., Bouslimani A., Petras D., Gauglitz J. M., Sikora N., Vargas F., van der Hooft J. J. J., Ernst M., Kang K. B., Aceves C. M., Caraballo-Rodríguez A. M., Koester I., Weldon K. C., Bertrand S., Roullier C., Sun K., Tehan R. M., Boya P. C. A., Christian M. H., Gutiérrez M., Ulloa A. M., Tejeda Mora J. A., Mojica-Flores R., Lakey-Beitia J., Vásquez-Chaves V., Zhang Y., Calderón A. I., Tayler N., Keyzers R. A., Tugizimana F., Ndlovu N., Aksenov A. A., Jarmusch A. K., Schmid R., Truman A. W., Bandeira N., Wang M., Dorrestein P. C. (2020). Reproducible Molecular Networking of Untargeted Mass
Spectrometry Data Using GNPS. Nat. Protoc..

[32] Schmid R., Heuckeroth S., Korf A., Smirnov A., Myers O., Dyrlund T. S., Bushuiev R., Murray K. J., Hoffmann N., Lu M., Sarvepalli A., Zhang Z., Fleischauer M., Dührkop K., Wesner M., Hoogstra S. J., Rudt E., Mokshyna O., Brungs C., Ponomarov K., Mutabdžija L., Damiani T., Pudney C. J., Earll M., Helmer P. O., Fallon T. R., Schulze T., Rivas-Ubach A., Bilbao A., Richter H., Nothias L.-F., Wang M., Orešič M., Weng J.-K., Böcker S., Jeibmann A., Hayen H., Karst U., Dorrestein P. C., Petras D., Du X., Pluskal T. (2023). Integrative Analysis
of Multimodal Mass Spectrometry Data in MZmine 3. Nat. Biotechnol..

[33] Pakkir
Shah A. K., Walter A., Ottosson F., Russo F., Navarro-Diaz M., Boldt J., Kalinski J.-C. J., Kontou E. E., Elofson J., Polyzois A. (2025). Statistical Analysis
of Feature-Based Molecular Networking Results from Non-Targeted Metabolomics
Data. Nat. Protoc..

[34] Khan A., Mathelier A. (2017). Intervene:
A Tool for Intersection and Visualization
of Multiple Gene or Genomic Region Sets. BMC
Bioinf..

[35] Petras D., Phelan V. V., Acharya D., Allen A. E., Aron A. T., Bandeira N., Bowen B. P., Belle-Oudry D., Boecker S., Cummings D. A., Deutsch J. M., Fahy E., Garg N., Gregor R., Handelsman J., Navarro-Hoyos M., Jarmusch A. K., Jarmusch S. A., Louie K., Maloney K. N., Marty M. T., Meijler M. M., Mizrahi I., Neve R. L., Northen T. R., Molina-Santiago C., Panitchpakdi M., Pullman B., Puri A. W., Schmid R., Subramaniam S., Thukral M., Vasquez-Castro F., Dorrestein P. C., Wang M. (2022). GNPS Dashboard: Collaborative Exploration
of Mass Spectrometry Data in the Web Browser. Nat. Methods.

[36] Petras D., Minich J. J., Cancelada L. B., Torres R. R., Kunselman E., Wang M., White M. E., Allen E. E., Prather K. A., Aluwihare L. I., Dorrestein P. C. (2021). Non-Targeted Tandem Mass Spectrometry
Enables the Visualization of Organic Matter Chemotype Shifts in Coastal
Seawater. Chemosphere.

[37] Stephens B. M., Stincone P., Petras D., English C. J., Opalk K., Giovannoni S., Carlson C. A. (2025). Oxidation State of Bioavailable Dissolved
Organic Matter Influences Bacterioplankton Respiration and Growth
Efficiency. Commun. Biol..

[38] Habra H., Kachman M., Padmanabhan V., Burant C., Karnovsky A., Meijer J. (2022). Alignment and Analysis
of a Disparately Acquired Multibatch
Metabolomics Study of Maternal Pregnancy Samples. J. Proteome Res..

[39] Hawkes J. A., D’Andrilli J., Agar J. N., Barrow M. P., Berg S. M., Catalán N., Chen H., Chu R. K., Cole R. B., Dittmar T., Gavard R., Gleixner G., Hatcher P. G., He C., Hess N. J., Hutchins R. H. S., Ijaz A., Jones H. E., Kew W., Khaksari M., Palacio Lozano D. C., Lv J., Mazzoleni L. R., Noriega-Ortega B. E., Osterholz H., Radoman N., Remucal C. K., Schmitt N. D., Schum S. K., Shi Q., Simon C., Singer G., Sleighter R. L., Stubbins A., Thomas M. J., Tolic N., Zhang S., Zito P., Podgorski D. C. (2020). An International
Laboratory Comparison of Dissolved Organic Matter Composition by High
Resolution Mass Spectrometry: Are We Getting the Same Answer?. Limnol. Oceanogr.:Methods.

[40] Clark T. N., Houriet J., Vidar W. S., Kellogg J. J., Todd D. A., Cech N. B., Linington R. G. (2021). Interlaboratory Comparison of Untargeted
Mass Spectrometry Data Uncovers Underlying Causes for Variability. J. Nat. Prod..

[41] Houriet J., Vidar W. S., Manwill P. K., Todd D. A., Cech N. B. (2022). How Low
Can You Go? Selecting Intensity Thresholds for Untargeted Metabolomics
Data Preprocessing. Anal. Chem..

[42] Sumner L. W., Amberg A., Barrett D., Beale M. H., Beger R., Daykin C. A., Fan T. W.-M., Fiehn O., Goodacre R., Griffin J. L., Hankemeier T., Hardy N., Harnly J., Higashi R., Kopka J., Lane A. N., Lindon J. C., Marriott P., Nicholls A. W., Reily M. D., Thaden J. J., Viant M. R. (2007). Proposed Minimum
Reporting Standards for Chemical Analysis:
Chemical Analysis Working Group (CAWG) Metabolomics Standards Initiative
(MSI). Metabolomics.

[43] Boulesteix A., Janitza S., Kruppa J., König I. R. (2012). Overview
of Random Forest Methodology and Practical Guidance with Emphasis
on Computational Biology and Bioinformatics. WIREs Data Min & Knowl.

[44] Percot A., Yalcin A., Aysel V., Erduğan H., Dural B., Güven K. C. (2009). Loliolide in Marine Algae. Nat. Prod. Res..

[45] Henatsch J. J., Jüttner F. (1983). Volatile Odorous Excretion Products
of Different Strains
of Synechococcus (Cyanobacteria). Water Sci.
Technol..

[46] Bogdanov A., Salib M. N., Chase A. B., Hammerlindl H., Muskat M. N., Luedtke S., da Silva E. B., O’Donoghue A. J., Wu L. F., Altschuler S. J., Molinski T. F., Jensen P. R. (2024). Small Molecule
in Situ Resin Capture Provides a Compound First Approach to Natural
Product Discovery. Nat. Commun..

[47] Grignon-Dubois M., Rezzonico B., Blanchet H. (2022). Phenolic Fingerprints
of the Pacific
Seagrass *Phyllospadix Torreyi* - Structural Characterization
and Quantification of Undescribed Flavonoid Sulfates. Phytochemistry.

[48] Felgate S. L., Craig A. J., Moodie L. W. K., Hawkes J. (2023). Characterization of
a Newly Available Coastal Marine Dissolved Organic Matter Reference
Material (TRM-0522). Anal. Chem..

[49] Haug K., Salek R. M., Steinbeck C. (2017). Global Open
Data Management in Metabolomics. Curr. Opin.
Chem. Biol..

[50] Ferreira J. D., Inácio B., Salek R. M., Couto F. M. (2017). Assessing Public
Metabolomics Metadata, Towards Improving Quality. J. integr. bioinform..

[51] Spicer R. A., Steinbeck C. (2018). A Lost Opportunity for Science: Journals Promote Data
Sharing in Metabolomics but Do Not Enforce It. Metabolomics.

[52] Charron-Lamoureux V., Mannochio-Russo H., Lamichhane S., Xing S., Patan A., Portal Gomes P. W., Rajkumar P., Deleray V., Caraballo-Rodríguez A. M., Chua K. V., Lee L. S., Liu Z., Ching J., Wang M., Dorrestein P. C. (2025). A Guide to Reverse Metabolomics—a
Framework for Big Data Discovery Strategy. Nat.
Protoc..

[53] Anderson R. F. (2020). GEOTRACES:
Accelerating Research on the Marine Biogeochemical Cycles of Trace
Elements and Their Isotopes. Ann. Rev. Mar.
Sci.

[54] Saito M. A., Alexander H., Benway H. M., Boyd P. W., Gledhill M., Kujawinski E. B., Levine N. M., Maheigan M., Marchetti A., Obernosterer I. (2024). The Dawn of the BioGeoSCAPES Program. Oceanogr..

