# Supplementary material for: Comparability of Liquid Chromatography Tandem Mass Spectrometry Analysis of Dissolved Organic Matter across Laboratories
Source: Environ Sci Technol. 2026 Feb 6;60(6):4814–29. doi: 10.1021/acs.est.5c12691 (PMC12918520; doi:10.1021/acs.est.5c12691)
Supplement: Supplementary file 1 [file es5c12691_si_001.pdf]

## Supplemental Information

# Comparability of Liquid Chromatography Tandem Mass Spectrometry Analysis of Dissolved Organic Matter Across Laboratories

Jarmo-Charles Kalinski[1,2,#], Bruno Ruiz Brandão da Costa[1,3,#], Tilman Schramm[1], Lance R. Buckett[4], Laura T. Carlson[5], Nicole R. Coffey[6], Tito Damiani[7], Elias Dechent[8], Yasin El Abiead[9], Steffen Heuckeroth[10], Elaine K. Jennings[11], Jan Kaesler[11], Naomi L. Stock[12], Alice M. Orme[13,14], Ralph R. Torres[15], Sara Trojahn[16,8], Helen L. Whelton[17], Yingfei Yan[4], Allegra T. Aron[18], Rene M. Boiteau[19], Ian D. Bull[17], Pieter C. Dorrestein[9], Duc Huy Dang[20,12], Richard P. Evershed[17], Marta Gledhill[21], Gerd Gleixner[13], Andreas F. Haas[22], Martin Hansen[23], Tilmann Harder[24], Ellen C. Hopmans[22], Anitra E. Ingalls[5], Uwe Karst[10], William Kew[25], Melissa Kido Soule[26], Boris P. Koch[24,27], Elizabeth B. Kujawinski[26], Oliver J. Lechtenfeld[11], Krista Longnecker[26], Tomáš Pluskal[7], Georg Pohnert[14], Zachary C. Redman[28], Albert Rivas-Ubach[29], Philippe Schmitt-Kopplin[4], Gabriel Singer[8], Jan Tebben[24], Patrick L. Tomco[28], Nicholas D. Ward[25], Lihini I. Aluwihare[15], Carsten Simon[11,30,31], Jeffrey Hawkes[32,*], Daniel Petras[1,33,*]

### Affiliations
1. Department of Biochemistry, University of California Riverside, Riverside, CA, 92521, USA
2. Rhodes University, Grahamstown (Makhanda), 6140, South Africa
3. University of São Paulo, São Paulo, 05508-000, Brazil
4. Helmholtz Munich, Analytical BioGeoChemistry, Neuherberg, 85764, Germany
5. School of Oceanography, University of Washington, Seattle, WA, 98195, USA
6. Department of Earth and Environmental Sciences, University of Minnesota, Minneapolis, MN, 55455, USA
7. Institute of Organic Chemistry and Biochemistry, Czech Academy of Sciences, Prague, 160 00, Czech Republic
8. Department of Ecology, University of Innsbruck, Innsbruck, 6020, Austria
9. Skaggs School of Pharmacy, University of California San Diego, La Jolla, CA, 92093, USA
10. Institute of Inorganic and Analytical Chemistry, University of Münster, Münster, 48149, Germany
11. Helmholtz Centre for Environmental Research (UFZ), Leipzig, 04318, Germany
12. Water Quality Centre, Trent University, Peterborough, Ontario, K9L 0G2, Canada
13. Max Planck Institute for Biogeochemistry, Jena, 07745, Germany
14. Institute of Inorganic and Analytical Chemistry, Friedrich Schiller University Jena, Jena, 07743, Germany
15. Scripps Institution of Oceanography, University of California San Diego, La Jolla, CA, 92037, USA
16. The James Hutton Institute, Aberdeen, AB15 8QH, United Kingdom
17. School of Chemistry, University of Bristol, Bristol, BS8 1TS, United Kingdom
18. Department of Chemistry, University of Denver, Denver, CO, 80208, USA
19. Department of Chemistry, University of Minnesota, Minneapolis, MN, 55455, USA
20. Trent School of the Environment and Chemistry Department, Trent University, Peterborough, Ontario, K9L 0G2, Canada
21. GEOMAR Helmholtz Centre for Ocean Research, Kiel, 24148, Germany
22. NIOZ Royal Netherlands Institute for Sea Research, Den Burg (Texel), 1790 AB, The Netherlands
23. Department of Environmental and Resource Engineering, Technical University of Denmark, Kongens Lyngby, 2800, Denmark
24. Alfred Wegener Institute, Bremerhaven, 27570, Germany
25. Pacific Northwest National Laboratory, Richland, WA, 99354, USA

26. Woods Hole Oceanographic Institution, Woods Hole, MA, 02543, USA
27. University of Applied Sciences Bremerhaven, Bremerhaven, 27568, Germany
28. Department of Chemistry, University of Alaska Anchorage, Anchorage, AK, 99508, USA
29. Instituto Nacional de Investigación y Tecnología Agraria y Alimentaria (INIA-CSIC), Madrid, 28040, Spain
30. Eawag, Swiss Federal Institute of Aquatic Science and Technology, Dübendorf, 8600, Switzerland
31. Inorganic Environmental Geochemistry, Institute of Biogeochemistry and Pollutant Dynamics, ETH Zurich, Zurich, 8092, Switzerland
32. Department of Chemistry, Uppsala University, Uppsala, 751 05, Sweden
33. CMFI Cluster of Excellence, University of Tübingen, Tübingen, 72074, Germany

[#]Contributed Equally
*Correspondence: jeffrey.hawkes@kemi.uu.se and dpetras@ucr.edu

## Table of Content

| | |
|---|---|
| **Suppl. Methods** | Global Data Analysis Procedure |
| **Table S1** | Final concentrations of study samples |
| **Table S2** | Exact masses of internal standards |
| **Table S3** | Overview of LC methods |
| **Table S4** | Overview of MS methods |
| **Table S5** | Summary of molecular networking metrics from all datasets |
| **Table S6** | Important Metabolites for Sample Classification from Random Forest Analyses |
| **Figure S1** | XICs of Cocaine for the 15 selected datasets |
| **Figure S2** | XICs of Domoic acid for the 15 selected datasets |
| **Figure S3** | XICs of Heroin for the 15 selected datasets |
| **Figure S4** | XICs of Imazapyr for the 15 selected datasets |
| **Figure S5** | XICs of Irgarol for the 15 selected datasets |
| **Figure S6** | XICs of Isoxaben for the 15 selected datasets |
| **Figure S7** | XICs of Kainic acid for the 15 selected datasets |
| **Figure S8** | XICs of Methamphetamine for the 15 selected datasets |
| **Figure S9** | XICs of Cocaine and Domoic acid for the excluded datasets |
| **Figure S10** | XICs of Heroin and Imazapyr for the excluded datasets |
| **Figure S11** | XICs of Irgarol and Isoxaben for the excluded datasets |
| **Figure S12** | XICs of Kainic acid and Methamphetamine for the excluded datasets |
| **Figure S13** | LC-MS/MS (ESI-) heatmaps |
| **Figure S14** | Distribution of Chimeric Spectra |
| **Figure S15** | Overview of shared (ESI+) LC-MS/MS CMN features and ubiquity across laboratories |
| **Figure S16** | Overview of shared (ESI-) LC-MS/MS FBMN features and ubiquity across laboratories |
| **Figure S17** | Overview of shared (ESI-) LC-MS/MS FBMN features and ubiquity across laboratories |
| **Figure S18** | FBMN–PCoAs (ESI+) of individual laboratories, all sample types. |
| **Figure S19** | FBMN–PCoAs (ESI-) of individual laboratories, all sample types. |
| **Figure S20** | CMN–PCoAs (ESI+) of individual laboratories, all sample types. |
| **Figure S21** | CMN–PCoAs (ESI-) of individual laboratories, all sample types. |
| **Figure S22** | PCoA and PERMANOVA using Bray-Curtis dissimilarity for CMN (ESI+) |
| **Figure S23** | PCoA and PERMANOVA for the FBMN and CMN without samples A (ESI+) |
| **Figure S24** | FBMN–PCoAs of individual laboratories, without samples A (ESI+) |
| **Figure S25** | CMN–PCoAs of individual laboratories, without samples A (ESI+) |
| **Figure S26** | Random Forest analysis results of features driving sample type clustering depicted as an UpSet plot |
| **Figure S27** | Random Forest analysis results of features driving sample type clustering depicted as an UpSet plot for CMN with merging of features based on identical spectral library annotation |

## Supplemental Methods: Global Data Analysis Procedure

Raw data was converted to .mzML format using msConvert. Feature finding for Feature-Based Molecular Networking (FBMN) was accomplished with mzmine (ver. 4.2.0). Mass detection thresholds of 1E5 for MS1 and 1E4 for MS2 were employed. The Chromatogram builder module was used to assemble chromatograms with a minimum of 3 consecutive scans, minimum intensity of 5E5 for consecutive scans, a minimum height of 1E6 and an *m/z* tolerance of 5 ppm. This was followed by the Local minimum feature resolver with a chromatographic threshold of 10%, a minimum search range of 0.1 min, a minimum absolute height of 1E6, a minimum peak top/edge ratio of 1.4, a peak duration range of 0.05 to 3 min and a minimum of 5 scans, while MS/MS scans were paired using the feature edges, *m/z* tolerances of 0.005 Da or 10 ppm and a minimum relative height of 25%. The 13C isotope filter was used with an *m/z* tolerance of 5 ppm and a retention time tolerance of 0.1 min and a maximum charge of 2. Individual feature lists were aligned with the Join aligner using an *m/z* tolerance of 5 ppm and a retention time tolerance of 1.7 min (weights = 3/1). The aligned feature list was filtered to contain only features that were contained in at least 10 samples and had an assigned MS/MS spectrum. Duplicate features were merged using the Duplicate peak filter module with the NEW AVERAGE mode, an *m/z* tolerance of 5 ppm and a retention time tolerance of 1.7 min. The final feature list and associated MS/MS spectra file were then exported and uploaded to GNPS2. The files were analyzed through the FBMN workflow using precursor and fragment ion tolerances of 0.02. Window and precursor window filters were activated, molecular networking was carried out with a minimum cosine of 0.7 and a minimum of 6 matched fragments. Network topology parameters were set to 10 for Top K and 100 for the maximum component size. Library search parameters were set to a minimum cosine of 0.7 and a minimum of 4 matched fragments. The same parameters were used for both ionization modes. The ESI+ FBMN job can be accessed at: https://gnps2.org/status?task=61e48c4af96944aeba73f59f0dbd51c2. The ESI- FBMN can be accessed at: https://gnps2.org/status?task=88f8fdb9665b42688b53cf560f4e23fd.

Classical Molecular Networking (CMN) was carried out using precursor and fragment ion tolerances of 0.02 Da. Window and precursor window filters were activated, molecular networking was carried out with a minimum cosine of 0.7, a minimum of 6 matched fragments and a minimum cluster size of 10. Network topology parameters were set to 10 for Top K and 100 for the maximum component size. Library search parameters were set to a minimum cosine of 0.7 and a minimum of 4 matched fragments. The ESI+ CMN job can be accessed at https://gnps2.org/status?task=cc2c071be92f428ca85188ae3654ea32 and the ESI- CMN job at https://gnps2.org/status?task=b8ad0450cf134dca9932394f856125f1.

Upset plots were generated with Intervene (https://asntech.shinyapps.io/intervene/) (Khan & Mathelier, 2017) and blank removal, PCoA plots and Random Forest Analysis (fixed random seed and 100 trees) were carried out using the FBMN-STATS APP (https://fbmn-statsguide.gnps2.org/; Kelminal et al., 2024) where data was generally imputed and normalized (TIC normalization). LC-MS/MS heatmaps and extracted ion chromatograms (XICs) were plotted using the GNPS dashboard (https://dashboard.gnps2.org/). All other plots and merging of feature tables based on identical matched library IDs were carried out using dedicated python scripts. Processed and

source files as well as python scripts can be accessed at ZENODO https://doi.org/10.5281/zenodo.16897529.

## Supplemental Tables

**Table S1: Final concentrations of study samples**

| Sample | A | A45M | A15M | A5M | M |
|---|---|---|---|---|---|
| Marine DOM [µg/mL] | 0 | 4500 | 4500 | 4500 | 4500 |
| Algae Extract [µg/mL] | 225 | 225 | 75 | 25 | 0.0 |
| Internal Standards [µg/mL] | 0 | 0.12 | 0.12 | 0.12 | 0.12 |

**Table S2: Exact masses of internal standards**

| Compound | Molecular Formula | Mass $[M+H]^+$ | Mass $[M+Na]^+$ |
|---|---|---|---|
| Domoic acid | $C_{15}H_{21}NO_6$ | 312.1442 | 334.1261 |
| Kainic acid | $C_{10}H_{15}NO_4$ | 214.1074 | 236.0893 |
| Isoxaben | $C_{18}H_{24}N_2O_4$ | 333.1809 | 355.1628 |
| Irgarol | $C_{11}H_{19}N_5S$ | 254.1434 | 276.1253 |
| Imazapyr | $C_{13}H_{15}N_3O_3$ | 262.1186 | 284.1006 |
| Heroin | $C_{21}H_{23}NO_5$ | 370.1649 | 392.1468 |
| Methamphetamine | $C_{10}H_{15}N$ | 150.1277 | 172.1097 |
| Cocaine | $C_{17}H_{21}NO_4$ | 304.1543 | 326.1363 |

## Table S3: Overview of LC methods

| Lab | LC Type | Column | Column Dimesions [mm] | Particle Size [µm] | Flow rate [mL/min] | Gradient Length [min] | Total Method length [min] | Gradient | Injection Volume [uL] |
|---|---|---|---|---|---|---|---|---|---|
| a | UHPLC | Phenomenex Kinetex C18 | 2.0 x 150 | 1.7 | 0.4 | 10 | 17 | 5% to 50% B (7 min), 50% to 99% B (10 min), 99% to 99% B (13min), 5% to 5% B (17Min) | 10 |
| b | UHPLC | Phenomenex Kinetex C18 | 2.0 x 150 | 1.7 | 0.4 | 10 | 17 | 5% to 50% B (7 min), 50% to 99% B (10 min), 99% to 99% B (13min), 5% to 5% B (17Min) | 5 |
| p | UHPLC | Phenomenex Kinetex C18 | 2.0 x 150 | 1.7 | 0.4 | 10 | 17 | 5% to 50% B (7 min), 50% to 99% B (10 min), 99% to 99% B (13min), 5% to 5% B (17Min) | 5 |
| c | UHPLC | Waters Acquity HSS T3 | 2.1 x 100 | 1.8 | 0.4 | 10 | 17 | 5% to 50% B (7 min), 50% to 99% B (10 min), 99% to 99% B (13min), 5% to 5% B (17Min) | 5 |
| d | UHPLC | Waters Acquity BEH C18 | 2.1 x 150 | 1.7 | 0.4 | 10 | 17 | 5% to 50% B (7 min), 50% to 99% B (10 min), 99% to 99% B (13min), 5% to 5% B (17Min) | 5 |
| e | UHPLC | Phenomenex Kinetex C18 | 2.1 x 150 | 1.7 | 0.5 | 10 | 18 | 5% to 50% B (7 min), 50% to 99% B (10 min), 99% to 99% B (17min), 5% (18Min) | 5 |
| f | UHPLC | Waters Acquity UPLC CSH C18 | 2.1 x 150 | 1.7 | 0.4 | 10 | 17 | 5% to 50% B (7 min), 50% to 99% B (10 min), 99% to 99% B (13min), 5% to 5% B (17Min) | 5 |
| g | UHPLC | Waters Acquity UPLC BEH C18 | 2.1 x 100 | 1.7 | 0.4 | 10 | 17 | 5% to 50% B (7 min), 50% to 99% B (10 min), 99% to 99% B (13min), 5% to 5% B (17Min) | 5 |
| q | UHPLC | Waters Cortecs C18 | 2.1 x 150 | 1.6 | 0.4 | 10 | 17 | 5% to 50% B (7 min), 50% to 99% B (10 min), 99% to 99% B (13min), 5% to 5% B (17Min) | 5 |
| h | HPLC | Hamilton PRP-C18 | 2.0 x 150 | 5 | 0.4 | 10 | 17 | 5% to 50% B (7 min), 50% to 99% B (10 min), 99% to 99% B (13min), 5% to 5% B (17Min) | 5 |
| r | UHPLC | Thermo Raptor C18 | 2.1 x 150 | 1.8 | 0.4 | 10 | 17 | 5% to 50% B (7 min), 50% to 99% B (10 min), 99% to 99% B (13min), 5% to 5% B (17Min) | 5 |
| i | UHPLC | Phenomenex Kinetex C18 | 2.1 x 150 | 1.7 | 0.4 | 10 | 17 | 5% to 50% B (7 min), 50% to 99% B (10 min), 99% to 99% B (13min), 5% to 5% B (17Min) | 5 |
| j | UHPLC | Phenomenex Kinetex C18 | 2.1 x 100 | 1.7 | 0.4 | 10 | 17 | 5% to 50% B (7 min), 50% to 99% B (10 min), 99% to 99% B (13min), 5% to 5% B (17Min) | 5 |
| s | UHPLC | Thermo Hypersil Gold aq | 2.1 x 100 | 1.9 | 0.4 | 10 | 17 | 5% to 50% B (7 min), 50% to 99% B (10 min), 99% to 99% B (13min), 5% to 5% B (17Min) | 5 |
| k | UHPLC | Thermo Hypersil Gold C18 | 2.1 x 150 | 1.9 | 0.4 | 10 | 17 | 5% to 50% B (7 min), 50% to 99% B (10 min), 99% to 99% B (13min), 5% to 5% B (17Min) | 5 |
| l | UHPLC | Phenomenex Kinetex C18 | 2.1 x 100 | 1.7 | 0.4 | 10 | 17 | 5% to 50% B (7 min), 50% to 99% B (10 min), 99% to 99% B (13min), 5% to 5% B (17Min) | 5 |
| t | UHPLC | Waters Acquity BEH C18 | 2.1 x 100 | 1.7 | 0.4 | 10 | 17 | 5% to 50% B (7 min), 50% to 99% B (10 min), 99% to 99% B (13min), 5% to 5% B (17Min) | 5 |
| m | HPLC | Grace Genesis C18 | 2.1 x 150 | 3 | 0.4 | 10 | 17 | 5% to 50% B (7 min), 50% to 99% B (10 min), 99% to 99% B (13min), 5% to 5% B (17Min) | 10 |
| u | UHPLC | Waters Acquity HSS T3 | 3 x 150 | 1.8 | 0.4 | 14 | 17 | 5% to 50% B (8 min), 50% to 99% B (11 min), 99% to 99% B (14min), 5% to 5% B (17Min) | 5 |
| v | Nano-LC | Thermo PepMap C18 | 0.05 x 150 | 2 | 0 | 15 | 40 | 10% to 95% (15 min) | 1 |
| n | UHPLC | Thermo Hypersil Gold | 2.1 x 100 | 1.9 | 0.4 | 10 | 17 | 5% to 50% B (7 min), 50% to 99% B (10 min), 99% to 99% B (13min), 5% to 5% B (17Min) | 5 |
| o | UHPLC | Thermo Hypersil Gold C18 | 2.1 x 100 | 1.9 | 0.4 | 10 | 17 | 5% to 50% B (7 min), 50% to 99% B (10 min), 99% to 99% B (13min), 5% to 5% B (17Min) | 5 |
| w | UHPLC | Phenomenex Kinetex C18 | 2.1 x 150 | 1.7 | 0.4 | 14 | 18 | 5% to 50% B (8 min), 50% to 99% B (11 min), 99% to 99% B (14min), 5% to 5% B (18Min) | 10 |
| x | UHPLC | Waters Cortecs C18 | 2.1 x 150 | 1.6 | 0.4 | 12 | 16 | 5% to 50% B (9 min), 50% to 95% B (11 min), 95% to 99% B (11.5 min), 95% to 5% B (14.5 min), 5% to 5% B (15.5 min) | 5 |

## Table S4: Overview of MS methods

| Lab | Mass Spectrometer | Mass Range | MS1 Resolution | MS2 Resolution | Micro Scans | Max Fill Time MS1 | Max Fill Time MS2 | AGC MS1 | AGC MS2 | Isolation Width [m/z] | Collision Energy | Apex Trigger Range | Minimum AGC MS2 | MS/MS Threshold Relative (optional) | MS/MS Threshold Absolute | TopN DDA | Dynamic Exclusion [sec] | DDA Duty Cycle Time [sec] |
|---|---|---|---|---|---|---|---|---|---|---|---|---|---|---|---|---|---|---|
| a | Q-Exactive | 150-1500 | 70000 | 17500 | 1 | 100 | 150 | 1.0E+06 | 1.0E+06 | 1 | 20,30,40 | 2-15 | 3.0E+04 | 3% | 2.0E+05 | 5 | 5 | <1 |
| b | Q-Exactive | 150-1500 | 70000 | 17500 | 1 | 100 | 150 | 1.0E+06 | 1.0E+06 | 1 | 20,30,40 | 2-15 | 3.0E+04 | N/A | N/A | 5 | 5 | <1 |
| p | LTQ-Orbitrap | 150-1500 | 120000 | 15000 | 1 | 100 | 100 | 1.0E+06 | 5.0E+04 | N/A | 35 | N/A | 2.5E+03 | N/A | N/A | 2 | 5 | ~1 |
| c | Orbitrap Fusion Lumos | 150-1500 | 120000 | 30000 | 1 | 100 | 100 | default | default | N/A | 20,30,40 | N/A | N/A | N/A | 2.5E+04 | 5 | 5 | ~1 |
| d | Orbitrap IDX | 150-1500 | 120000 | 30000 | 1 | 256 | 100 | 2.0E+05 | 5.0E+04 | N/A | 20,30,40 | 3 | N/A | N/A | 1.0E+05 | 5 | 5 | <1 |
| e | Q-Exactive | 150-1500 | 140000 | 17500 | 1 | 100 | 150 | 1.0E+06 | 1.0E+05 | N/A | 20,30,40 | 2-15 | 8.0E+03 | N/A | 5.3E+04 | 5 | 5 | <1 |
| f | Q-Exactive HF | 150-1500 | 60000 | 30000 | 1 | 100 | 150 | 1.0E+06 | 1.0E+05 | N/A | 20,30,40 | 2-15 | 8.0E+03 | N/A | 5.3E+04 | 5 | 5 | <1 |
| g | Q-Exactive+ | 150-1500 | 140000 | 35000 | 1 | 50 | 100 | 1.0E+06 | 1.0E+05 | 1 | 20,30,40 | 2-15 | 8.0E+03 | N/A | 1.6E+05 | 5 | 5 | <1 |
| q | QTOF, maXis | 150-1500 | 40000 | 40000 | | 100 | 200 | N/A | N/A | 4 | 35 | N/A | N/A | N/A | 2.0E+03 | 5 | 5 | ~1 |
| h | Q-Exactive | 150-1500 | 70000 | 17500 | 1 | 100 | 150 | 1.0E+06 | 1.0E+05 | N/A | 20,30,40 | 2-15 | 3.0E+04 | N/A | 2.0E+05 | 5 | 10 | ~1 |
| r | Orbitrap ID-X | 150-1500 | 120000 | 30000 | 1 | 100 | 100 | default | default | N/A | 20,30,40 | | | N/A | 2.5E+04 | 5 | 5 | ~1 |
| i | Q-Exactive HF | 150-1500 | 60000 | 30000 | 1 | 100 | 200 | 3.0E+06 | 1.0E+05 | 1 | 20,30,40 | 2-15 | 8.0E+03 | N/A | 4.0E+04 | 5 | 5 | ~1 |
| j | Q-Exactive | 150-1500 | 70000 | 17500 | 1 | 100 | 150 | 3.0E+06 | 1.0E+05 | N/A | 20,30,40 | 2-15 | 5.0E+03 | N/A | 1.6E+05 | 5 | 5 | <1 |
| s | QTOF, Synapt g2 | 50-1500 | 40000 | 20000 | N/A | N/A | N/A | N/A | N/A | N/A | N/A | N/A | N/A | N/A | N/A | N/A | N/A | N/A |
| k | Q-Exactive HF-X | 150-1500 | 120000 | 30000 | 1 | 100 | 200 | 3.0E+06 | 1.0E+05 | N/A | 20,30,40 | 2-15 | 8.0E+03 | N/A | 4.0E+04 | 5 | 5 | ~1 |
| l | Orbitrap Exploris 120 | 150-1500 | 120000 | 60000 | 1 | 100 | 150 | 1.0E+06 | 1.0E+05 | N/A | 20,30,40 | 30% | N/A | N/A | 2.0E+05 | 3 | 5 | ~1 |
| t | Orbitrap Fusion | 150-1500 | 120000 | 30000 | 1 | 100 | 200 | 5.0E+05 | 2.5E+04 | N/A | 20,30,40 | | N/A | N/A | 2.5E+04 | 5 | 5 | ~1 |
| m | Q-Exactive | 150-1500 | 140000 | 35000 | 1 | 100 | 150 | 3.0E+06 | 1.0E+05 | N/A | 20,30,40 | 2-15 | 8.0E+03 | N/A | 5.3E+04 | 5 | 5 | <1 |
| u | SolariX xR, 12T | 147.5-150 | 512000 | 512000 | N/A | 100 | 400 | N/A | N/A | 5 | 10 V | N/A | N/A | 5% | N/A | N/A | 5 | N/A |
| v | Q-Exactive HF | 100-1500 | 240000 | 30000 | 1 | 100 | 100 | 1000000 | 100000 | 1 | 20,30,40 | 6-12 | 1.0E+03 | 0.1 | 1.0E+04 | N/A | 20 | ~1 |
| n | Orbitrap Exploris 480 | 150-1500 | 120000 | 30000 | 1 | 100r | 100r | 1.0E+06 | 1.0E+05 | 1 | 20,30,40 | 30% | N/A | N/A | 5.0E+03 | 5 | 5 | ~1 |
| o | Q-Exactive | 150-1500 | 70000 | 17500 | 1 | 100 | 150 | 1.0E+06 | 1.0E+05 | | 20,30,40 | 2-15 | 1.0E+04 | N/A | 6.7E+04 | 5 | 5 | ~1 |
| w | Orbitrap Fusion Lumos | 150-1500 | 240000 | 30000 | 1 | 100 | 100 | 2.00E+05 | 5.00E+04 | 0.8 | 20,30,40 | N/A | N/A | 20 | 2.5E+04 | dynamic | exclusion list | 1 |
| x | QTOF, X500R | 50-1600 | 42000 | 27000 | N/A | N/A | N/A | N/A | N/A | 1 | 30 | N/A | N/A | N/A | 500 | 10 | 6 | 0.6 |

**Table S5: Summary of molecular networking metrics from all datasets.** Metrics include the presence or absence (✓/✗) of internal standard annotation during classical molecular networking (CMN), as well as the number of library ID matches, clustered features (clusters), networked nodes, singletons, and annotation rates obtained from both CMN and feature-based molecular networking (FBMN).

| | | | | | | | | | | | CMN+ | | | FBMN+ | | | CMN- | | | FBMN- | | |
|---|---|---|---|---|---|---|---|---|---|---|---|---|---|---|---|---|---|---|---|---|---|---|
| Lab | Instrument Platform | Annotated Standards | Kainic acid | Imazapyr | Cocaine | Domoic acid | Isoxaben | Heroin | Irgarol | Methamphetamine | Lib IDs | Clusters | Networked Nodes | Lib Ids | Nodes | Networked Nodes | Lib IDs | Clusters | Networked Nodes | Lib Ids | Nodes | Networked Nodes |
| i | Q-Exactive HF | 6 | ✓ | ✓ | ✓ | ✓ | ✗ | ✓ | ✓ | ✗ | 158 | 2677 | 1114 | 264 | 1827 | 1103 | 54 | 2153 | 842 | 4 | 447 | 201 |
| j | Q-Exactive Plus | 8 | ✓ | ✓ | ✓ | ✓ | ✓ | ✓ | ✓ | ✓ | 145 | 2937 | 1687 | 577 | 3112 | 2441 | 40 | 2012 | 1283 | 17 | 1076 | 551 |
| o | Q-Exactive | 6 | ✗ | ✓ | ✓ | ✓ | ✓ | ✗ | ✓ | ✓ | 136 | 5061 | 2760 | 573 | 4569 | 3088 | 26 | 2228 | 1331 | 8 | 1071 | 679 |
| g | Q-Exactive Plus | 8 | ✓ | ✓ | ✓ | ✓ | ✓ | ✓ | ✓ | ✓ | 122 | 2602 | 1457 | 401 | 2431 | 1755 | 45 | 1770 | 1039 | 10 | 648 | 346 |
| k | Q-Exactive HFX | 7 | ✗ | ✓ | ✓ | ✓ | ✓ | ✓ | ✓ | ✓ | 106 | 5058 | 2776 | 340 | 4624 | 3414 | 68 | 3626 | 1513 | 9 | 1922 | 915 |
| b | Q-Exactive | 8 | ✓ | ✓ | ✓ | ✓ | ✓ | ✓ | ✓ | ✓ | 106 | 2451 | 1502 | 187 | 1789 | 1267 | 16 | 526 | 328 | 3 | 101 | 24 |
| e | Q-Exactive | 7 | ✓ | ✓ | ✓ | ✓ | ✓ | ✗ | ✓ | ✓ | 101 | 2699 | 1378 | 348 | 2401 | 1662 | 26 | 2052 | 951 | 8 | 693 | 377 |
| f | Q-Exactive HF | 6 | ✗ | ✓ | ✓ | ✓ | ✗ | ✓ | ✓ | ✓ | 101 | 2958 | 1244 | 293 | 2938 | 1506 | 40 | 1541 | 593 | 20 | 520 | 179 |
| l | Orbitrap Exploris 120 | 6 | ✓ | ✓ | ✓ | ✓ | ✗ | ✗ | ✓ | ✓ | 99 | 2625 | 1609 | 354 | 2342 | 1743 | 38 | 1998 | 1194 | 12 | 811 | 551 |
| c | Orbitrap Fusion Lumos | 7 | ✓ | ✓ | ✓ | ✓ | ✓ | ✗ | ✓ | ✓ | 98 | 4003 | 1995 | 389 | 2756 | 1934 | 45 | 2555 | 1251 | 8 | 445 | 271 |
| m | Q-Exactive | 7 | ✓ | ✓ | ✓ | ✓ | ✓ | ✗ | ✓ | ✓ | 90 | 1375 | 723 | 183 | 987 | 657 | 10 | 570 | 140 | 3 | 301 | 24 |
| n | Orbitrap Exploris 480 | 7 | ✓ | ✓ | ✓ | ✓ | ✓ | ✓ | ✗ | ✓ | 86 | 2394 | 1180 | 306 | 1962 | 1363 | 3 | 1922 | 914 | 4 | 181 | 85 |
| d | Orbitrap IDX | 8 | ✓ | ✓ | ✓ | ✓ | ✓ | ✓ | ✓ | ✓ | 83 | 3771 | 1866 | 423 | 3520 | 2370 | 25 | 3351 | 1690 | 13 | 1026 | 686 |
| h | Q-Exactive | 7 | ✗ | ✓ | ✓ | ✓ | ✓ | ✓ | ✓ | ✓ | 74 | 2428 | 1545 | 175 | 1304 | 971 | 33 | 1916 | 1213 | 11 | 558 | 295 |
| a | Q-Exactive | 7 | ✓ | ✓ | ✓ | ✗ | ✓ | ✓ | ✓ | ✓ | 71 | 2217 | 1121 | 275 | 1962 | 1243 | 24 | 820 | 364 | 8 | 496 | 234 |
| s | Synapt G2 | 0 | ✗ | ✗ | ✗ | ✗ | ✗ | ✗ | ✗ | ✗ | 3 | 136 | 72 | 0 | 0 | 0 | 12 | 117 | 87 | **NA** | **NA** | **NA** |
| p | LTQ-Orbitrap Elite | 4 | ✗ | ✓ | ✓ | ✓ | ✗ | ✗ | ✗ | ✓ | 7 | 210 | 36 | 2 | 777 | 26 | 30 | 1082 | 206 | 0 | 174 | 0 |
| q | QTOF maXis | 5 | ✗ | ✓ | ✓ | ✗ | ✓ | ✓ | ✓ | ✗ | 18 | 604 | 310 | 6 | 948 | 46 | 0 | 45 | 0 | **NA** | **NA** | **NA** |
| t | Orbitrap Fusion | 5 | ✓ | ✓ | ✓ | ✗ | ✓ | ✓ | ✗ | ✗ | 91 | 2632 | 1325 | 202 | 1996 | 1394 | 48 | 1956 | 933 | 3 | 148 | 89 |
| u | FT-ICR | 0 | ✗ | ✗ | ✗ | ✗ | ✗ | ✗ | ✗ | ✗ | 0 | 3741 | 294 | 0 | 57 | 2 | 2 | 484 | 421 | **NA** | **NA** | **NA** |
| v | Q-Exactive HF | 5 | ✓ | ✓ | ✓ | ✓ | ✗ | ✗ | ✗ | ✓ | 107 | 4262 | 1969 | 464 | 3999 | 2185 | 65 | 4235 | 2100 | 8 | 952 | 443 |
| w | Orbitrap Fusion Lumos | 5 | ✗ | ✓ | ✓ | ✓ | ✓ | ✓ | ✗ | ✗ | 69 | 501 | 299 | 79 | 288 | 213 | 36 | 712 | 445 | 14 | 149 | 113 |
| x | QTOF X500R | 8 | ✓ | ✓ | ✓ | ✓ | ✓ | ✓ | ✓ | ✓ | 61 | 3460 | 789 | 5 | 757 | 5 | 35 | 1983 | 381 | 2 | 146 | 23 |
| r | Orbitrap ID-X | 0 | ✗ | ✗ | ✗ | ✗ | ✗ | ✗ | ✗ | ✗ | **NA** | **NA** | **NA** | **NA** | **NA** | **NA** | 22 | 1835 | 819 | 8 | 176 | 102 |

## Table S6: Important Metabolites for Sample Classification according to sample-set wise Random Forest Analyses.

| Annotation | ID | count | rank | | a | b | c | d | e | f | g | h | i | j | k | l | m | n | o |
|---|---|---|---|---|---|---|---|---|---|---|---|---|---|---|---|---|---|---|---|
| dihydroactinidiolide | 26 | 15 | 1 | | x | x | x | x | x | x | x | x | x | x | x | x | x | x | x |
| cocamidopropyl betaine | 228 | 15 | 2 | | x | x | x | x | x | x | x | x | x | x | x | x | x | x | x |
| Cabrillostatin | 85 | 15 | 3 | | x | x | x | x | x | x | x | x | x | x | x | x | x | x | x |
| 6-Methoxyluteolin | 54 | 15 | 4 | | x | x | x | x | x | x | x | x | x | x | x | x | x | x | x |
| METHYL STEARATE | 138 | 15 | 5 | | x | x | x | x | x | x | x | x | x | x | x | x | x | x | x |
| Imazapic | 116 | 15 | 6 | | x | x | x | x | x | x | x | x | x | x | x | x | x | x | x |
| Melibiose | 144 | 15 | 7 | | x | x | x | x | x | x | x | x | x | x | x | x | x | x | x |
| tris(2-butoxyethyl) phosphate | 274 | 14 | 8 | | x | x | x | | x | x | x | x | x | x | x | x | x | x | x |
| 1-Myristoyl-sn-glycero-3-phosphocholine | 16 | 13 | 9 | | x | x | x | x | x | | x | x | x | x | x | x | | x | x |
| Loliolide | 132 | 14 | 10 | | x | x | x | x | | x | x | x | x | x | x | x | x | x | x |
| triphenyl phosphate | 273 | 12 | 11 | | x | x | | x | x | x | x | x | x | x | x | | | x | x |
| Methyl hexadecanoate | 146 | 13 | 12 | | | x | x | x | x | x | x | x | x | x | x | x | | x | x |
| Morphine | 152 | 14 | 13 | | x | x | x | x | x | x | x | x | x | x | x | x | | x | x |
| Dimethyl sebacate | 99 | 12 | 14 | | x | | | x | x | x | x | x | | x | x | x | x | x | x |
| 1-Hexadecanoyl-sn-glycerol | 15 | 15 | 15 | | x | x | x | x | x | x | x | x | x | x | x | x | x | x | x |
| Monoolein | 150 | 11 | 16 | | | x | | x | x | x | x | x | | x | x | x | | x | x |
| Glu Phe | 104 | 14 | 17 | | x | x | x | x | x | x | x | x | x | x | x | x | x | x | |
| Benzyldodecyldimethylammonium | 81 | 15 | 18 | | x | x | x | x | x | x | x | x | x | x | x | x | x | x | x |
| 1,3-Diphenylguanidine | 12 | 13 | 19 | | x | x | x | x | x | x | | x | x | x | x | x | | x | x |
| DEET | 96 | 14 | 20 | | x | x | x | x | x | x | x | x | x | x | x | x | x | x | |
| Monopalmitolein (9c) | 151 | 15 | 21 | | x | x | x | x | x | x | x | x | x | x | x | x | x | x | x |
| 7512-17-6 | 57 | 13 | 22 | | x | x | x | x | x | x | x | x | | x | x | | x | x | x |
| 2,6-Dimethoxy-4-methylphenol | 30 | 13 | 23 | | x | x | x | x | x | x | x | x | | x | x | x | x | x | |
| delorazepam | 231 | 10 | 24 | | x | | | | x | x | | x | x | x | x | x | | x | x |
| GLYCERO-3-PHOSPHOCHOLINE | 103 | 13 | 25 | | x | x | x | x | | x | x | x | x | x | x | x | x | | x |
| myristamidpropyl betaine | 252 | 12 | 26 | | x | x | x | x | x | x | x | x | x | x | | | x | x | |
| L-Tryptophan | 122 | 13 | 27 | | x | x | x | x | x | x | x | x | x | x | | x | | x | x |
| Shinorine | 196 | 14 | 28 | | x | x | x | x | x | x | x | | x | x | x | x | x | x | x |
| Phenylalanine methyl ester | 179 | 15 | 29 | | x | x | x | x | x | x | x | x | x | x | x | x | x | x | x |
| diethyl phthalate | 234 | 13 | 30 | | | | x | x | x | x | x | x | x | x | x | x | x | x | x |
| dihydrocapsaicin | 237 | 8 | 31 | | x | | | | x | | x | | x | | x | x | x | x | |
| 1-Palmitoylglycerophosphocholine | 18 | 10 | 32 | | x | x | x | x | | | x | x | x | x | x | | | | x |
| Phe-ala | 178 | 13 | 33 | | x | x | x | x | x | x | x | x | | x | x | x | | x | x |
| Hexaethylene glycol | 111 | 13 | 34 | | x | x | x | x | x | x | x | x | x | | | x | x | x | x |
| triethyl citrate | 272 | 10 | 35 | | x | x | x | | x | x | x | x | | | | x | | x | x |
| 3.10S-Hydroxypheophorbide a | 39 | 10 | 36 | | x | x | | x | x | x | x | | | x | x | x | | | x |
| 4-Methylphthalic anhydride | 44 | 12 | 37 | | x | | x | x | x | x | | x | x | x | x | x | x | x | |
| ibuprofen | 243 | 12 | 38 | | x | x | x | x | x | | | x | x | x | | x | x | x | x |
| laurolactam | 246 | 12 | 39 | | x | x | x | x | x | x | x | x | x | x | x | | | x | |
| sultiame | 265 | 11 | 40 | | x | | | x | x | x | | x | x | x | x | x | | x | x |
| Sebacic acid monomethyl ester | 193 | 10 | 41 | | | | | x | x | | x | x | | x | x | x | x | x | x |
| Ile-Glu | 115 | 12 | 42 | | x | x | x | x | x | x | | x | x | | x | x | x | x | |
| benzyltetradecyldimethyl ammonium | 218 | 11 | 43 | | x | x | x | x | x | x | x | x | | | x | | x | x | |
| 4-Hydroxy-1-(2-hydroxyethyl)-2,2,6,6-tetramethylpiperidine | 43 | 13 | 44 | | x | x | x | x | x | x | x | x | x | | x | x | | x | x |
| 9,12-Octadecadiynoic Acid | 61 | 13 | 45 | | x | x | x | x | x | x | x | | x | x | x | x | | x | x |
| Pentapropylene glycol | 175 | 13 | 46 | | x | x | x | | x | x | x | x | x | | x | x | x | x | x |
| PHENYLALANINE | 168 | 12 | 47 | | x | x | x | x | x | x | x | x | | | x | x | x | | x |
| Val-Leu | 210 | 11 | 48 | | | | x | x | x | x | x | x | | x | x | x | | x | x |
| Palythine | 171 | 12 | 49 | | x | x | | x | x | | x | x | x | x | x | x | x | x | |
| Dioctyl phthalate | 100 | 11 | 50 | | x | | x | x | x | x | | | x | x | x | x | | x | x |
| N-[3-(dimethylamino)propyl]dodecanamide | 157 | 12 | 51 | | | x | x | x | x | x | x | x | | x | x | x | | x | x |
| cis-9-Hexadecenoic acid | 226 | 12 | 52 | | x | x | x | | x | x | | x | x | x | x | x | | x | x |
| Chicoric acid | 86 | 13 | 53 | | x | | | x | x | x | x | x | x | x | x | x | x | x | x |
| domoic acid | 240 | 13 | 54 | | x | x | x | x | x | x | x | x | x | | x | x | | x | x |
| Methyl-Domoic Acid | 147 | 12 | 55 | | x | x | x | x | x | x | x | x | x | | | x | x | x | |
| B08A23 | 77 | 11 | 56 | | x | x | x | x | | x | | x | | x | x | x | | x | x |
| diphenyl phosphate | 239 | 9 | 57 | | x | x | | x | x | x | x | | | x | | x | | x | |
| SQDG(16:0/16:1) | 189 | 10 | 58 | | x | | | x | | x | | x | x | x | | x | x | x | x |
| Tri(propylene glycol) butyl ether | 204 | 13 | 59 | | x | x | x | x | x | x | x | x | | x | x | x | | x | x |
| Vanillin | 211 | 9 | 60 | | x | | x | x | x | | | x | | x | x | x | | | x |

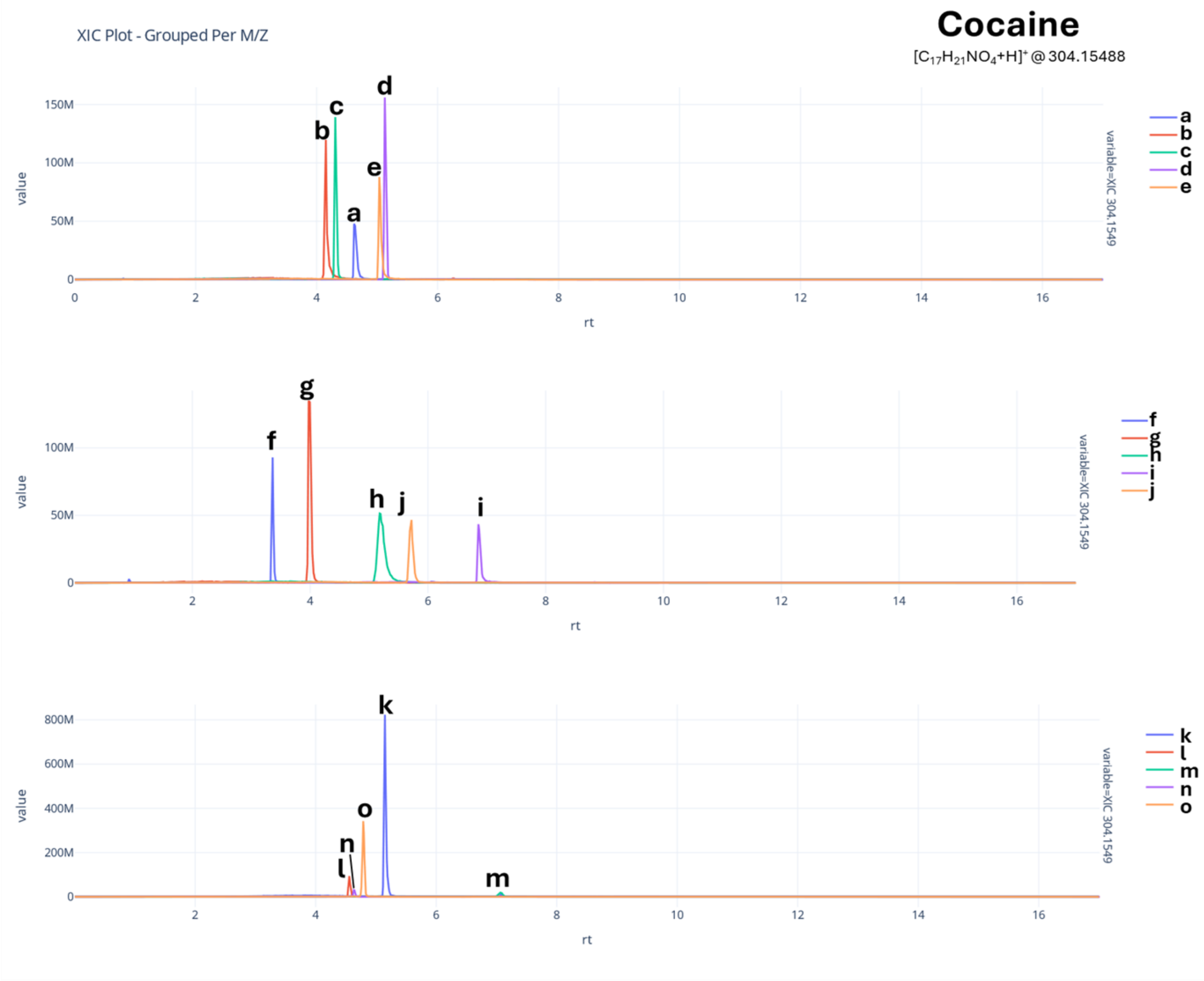


**Figure S1:** XICs of Cocaine for the 15 selected datasets.

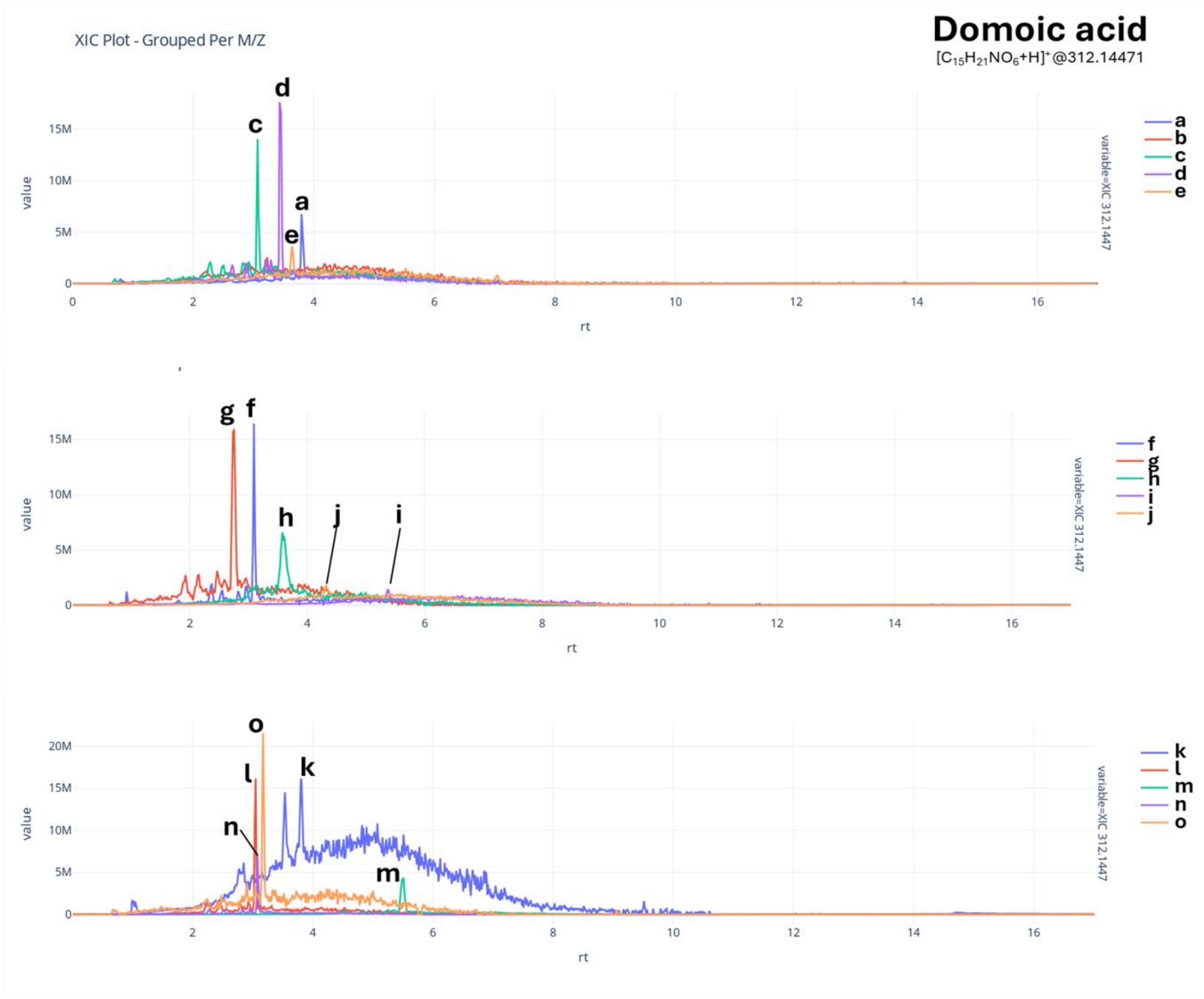


**Figure S2:** XICs of Domoic acid for the 15 selected datasets.

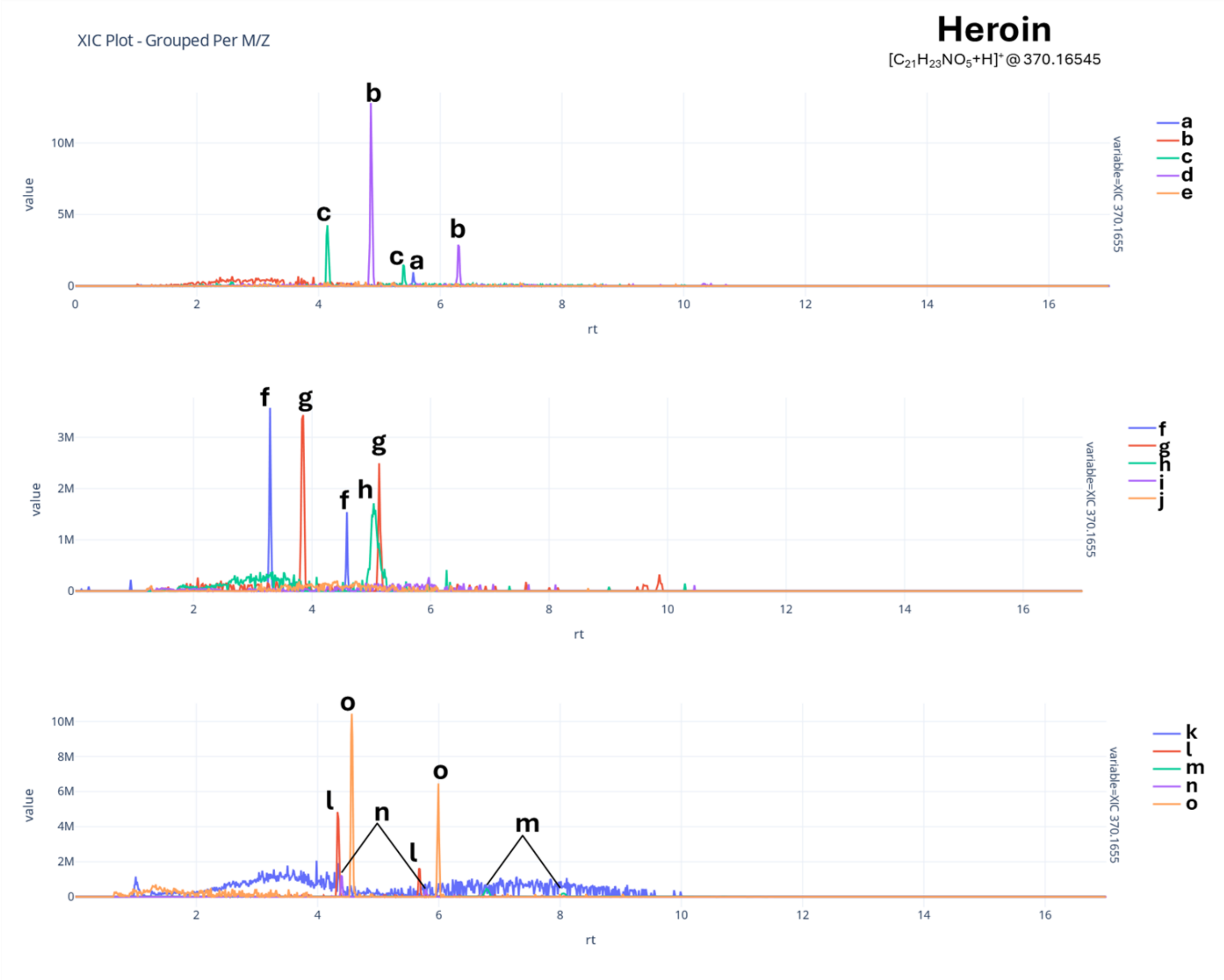


**Figure S3:** XICs of Heroin for the 15 selected datasets.

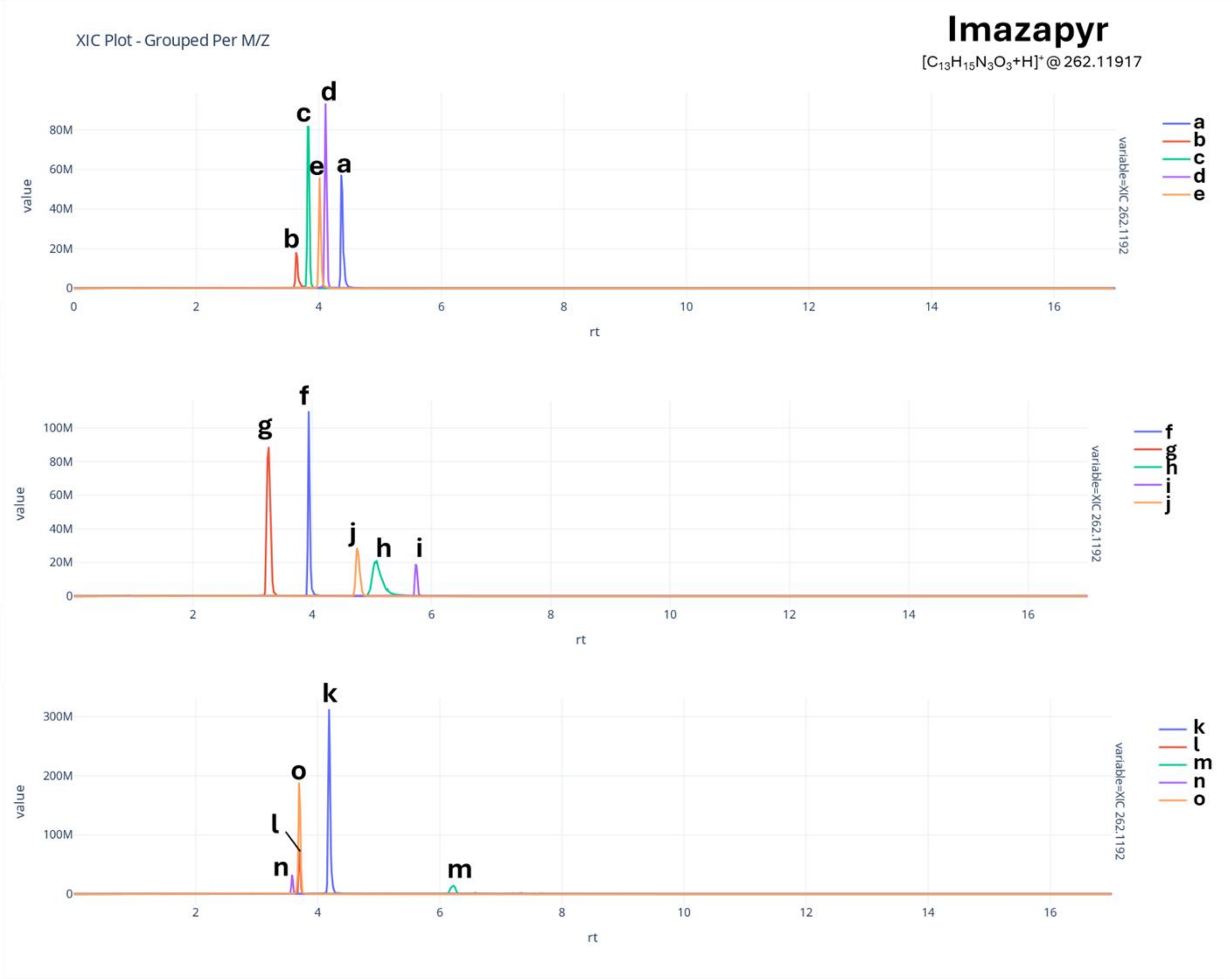


**Figure S4:** XICs of Imazapyr for the 15 selected datasets.

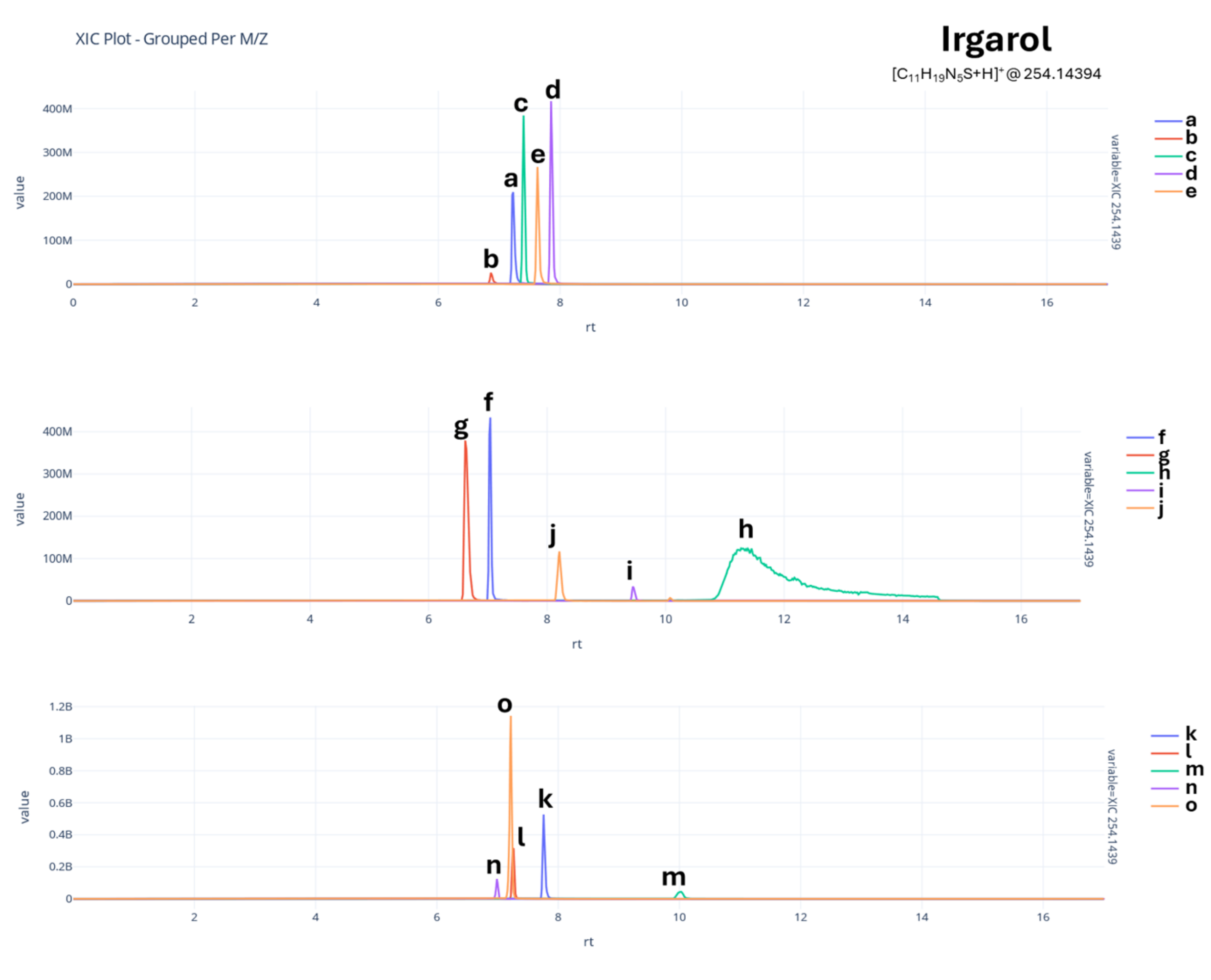


**Figure S5:** XICs of Irgarol for the 15 selected datasets.

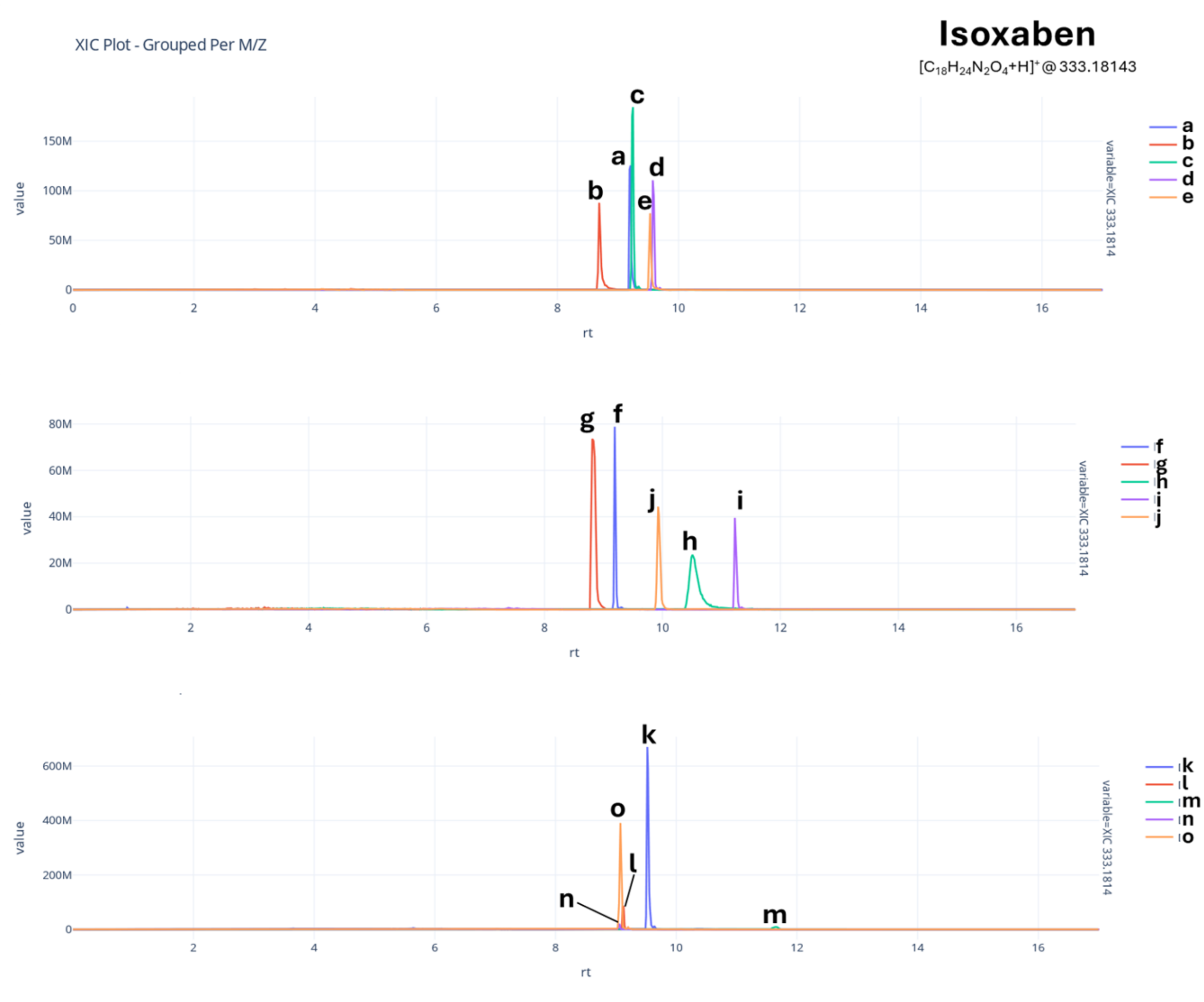


**Figure S6:** XICs of Isoxaben for the 15 selected datasets.

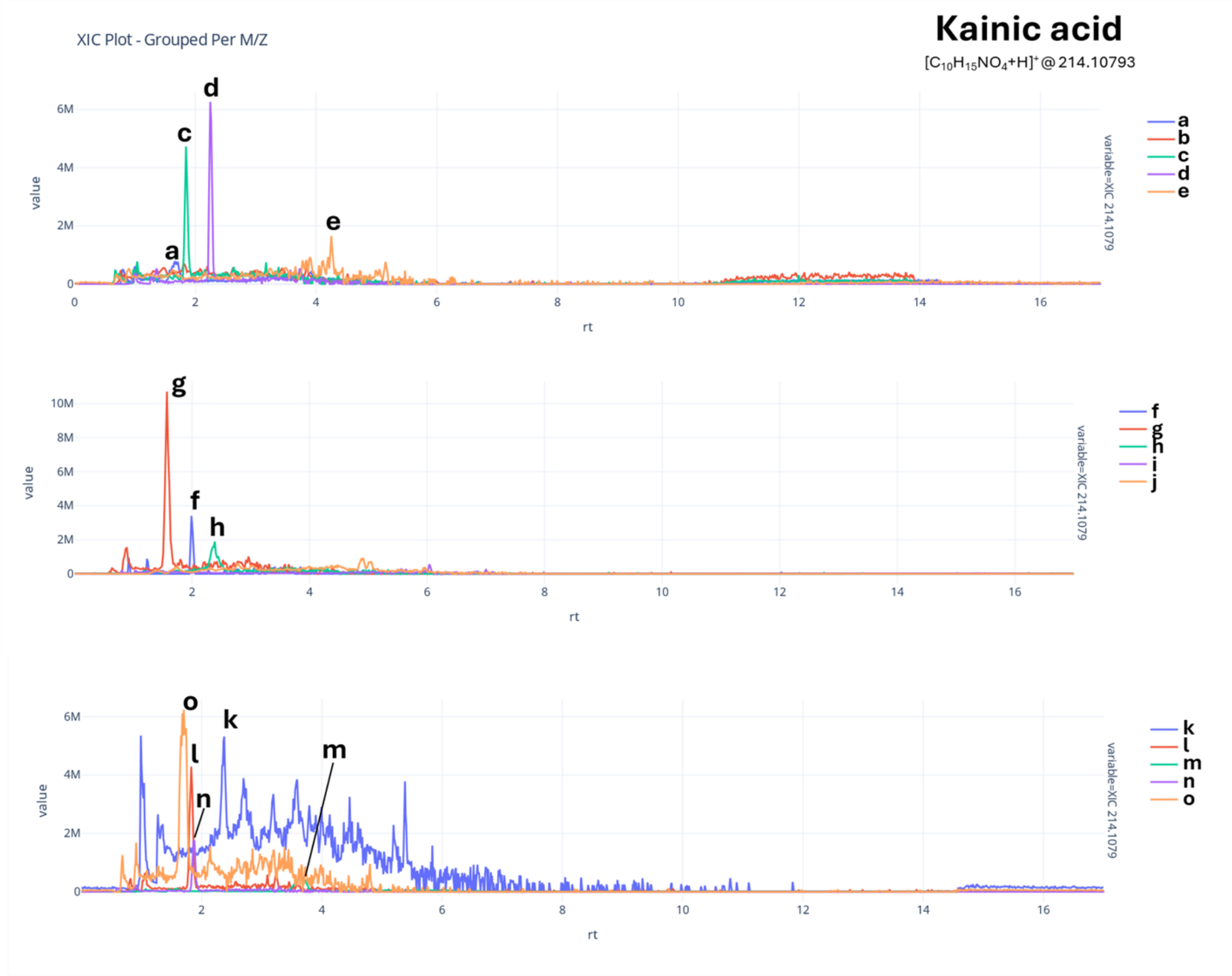


**Figure S7:** XICs of Kainic acid for the 15 selected datasets.

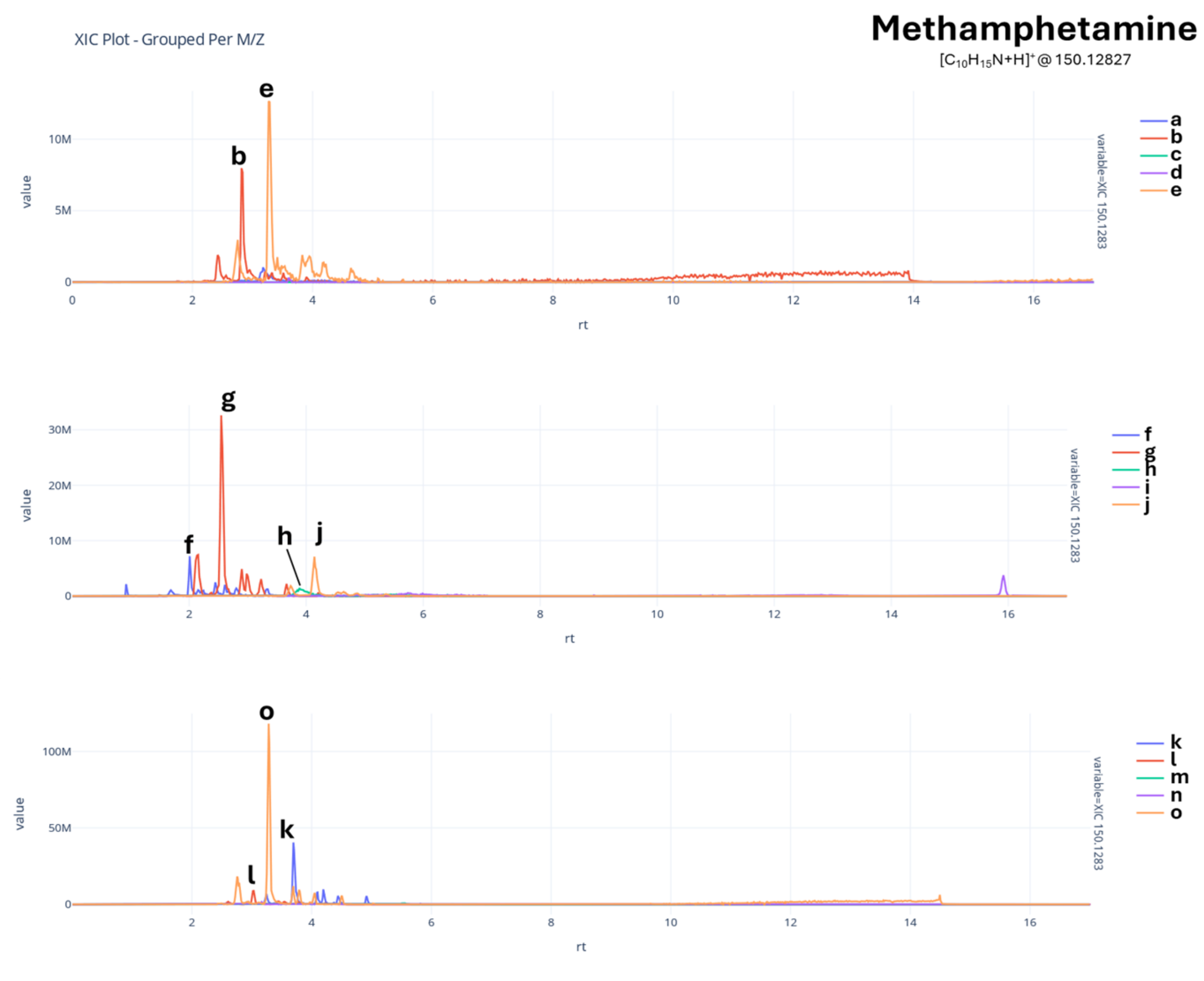


**Figure S8:** XICs of Methamphetamine for the 15 selected datasets.

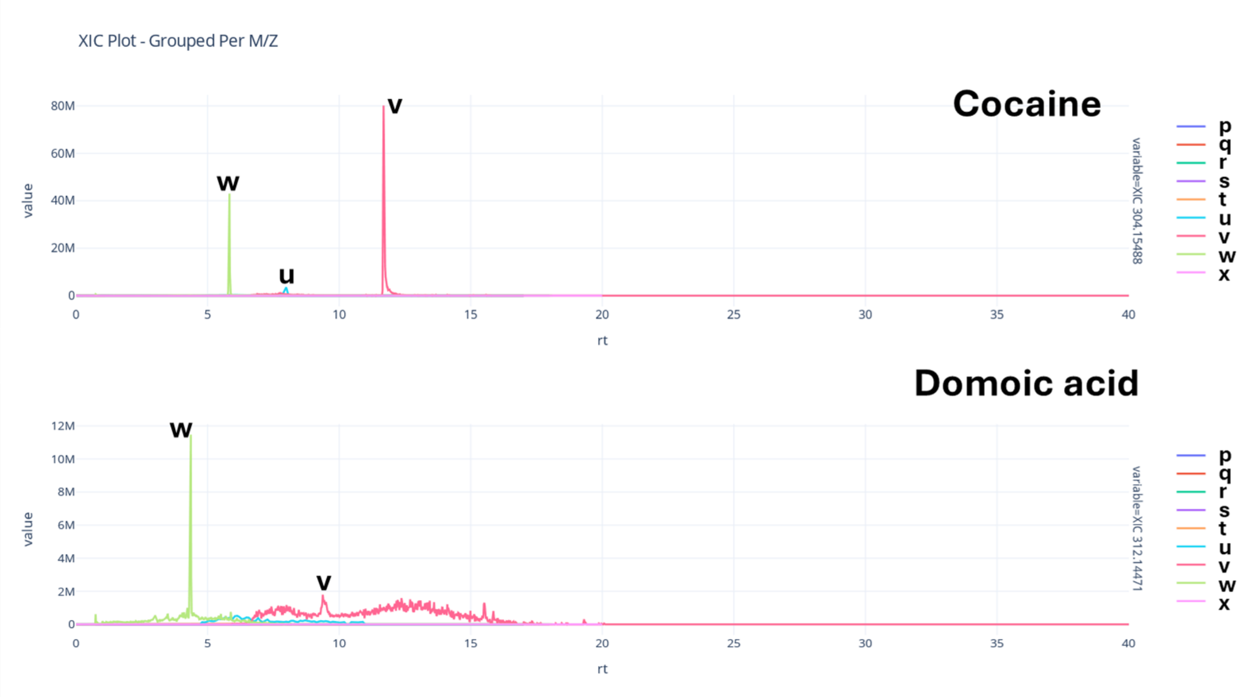


**Figure S9:** XICs of Cocaine and Domoic acid for the excluded datasets.

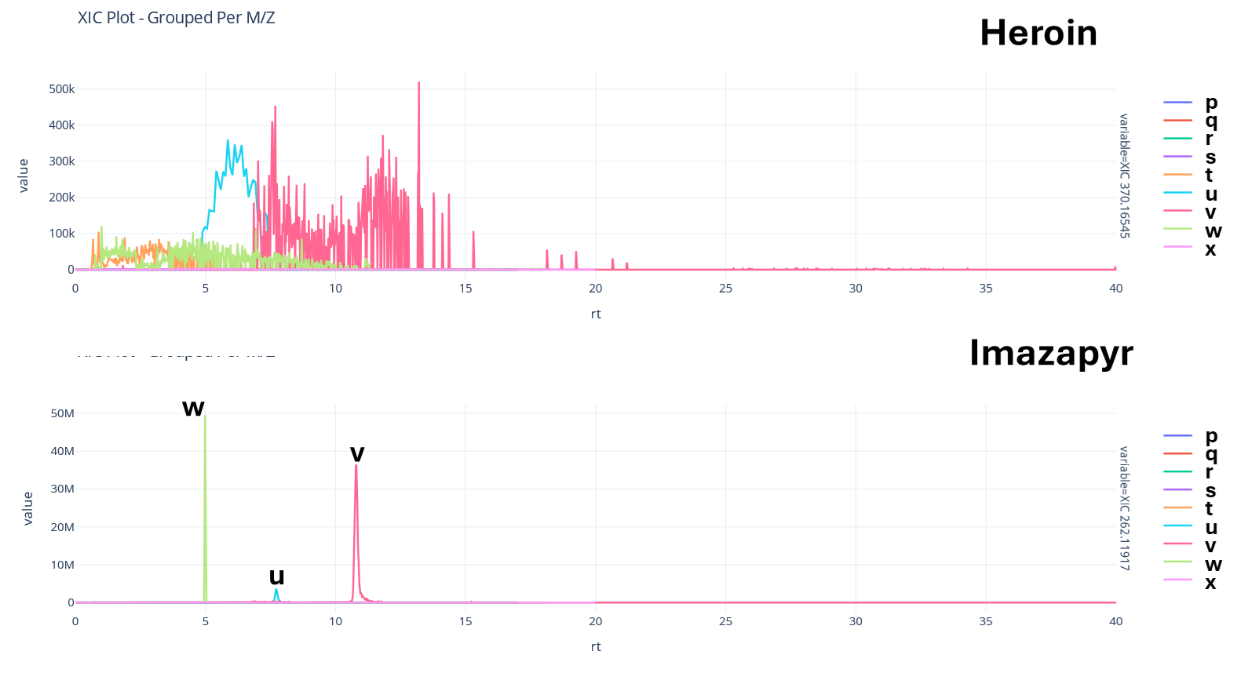


**Figure S10:** XICs of Heroin and Imazapyr for the excluded datasets.

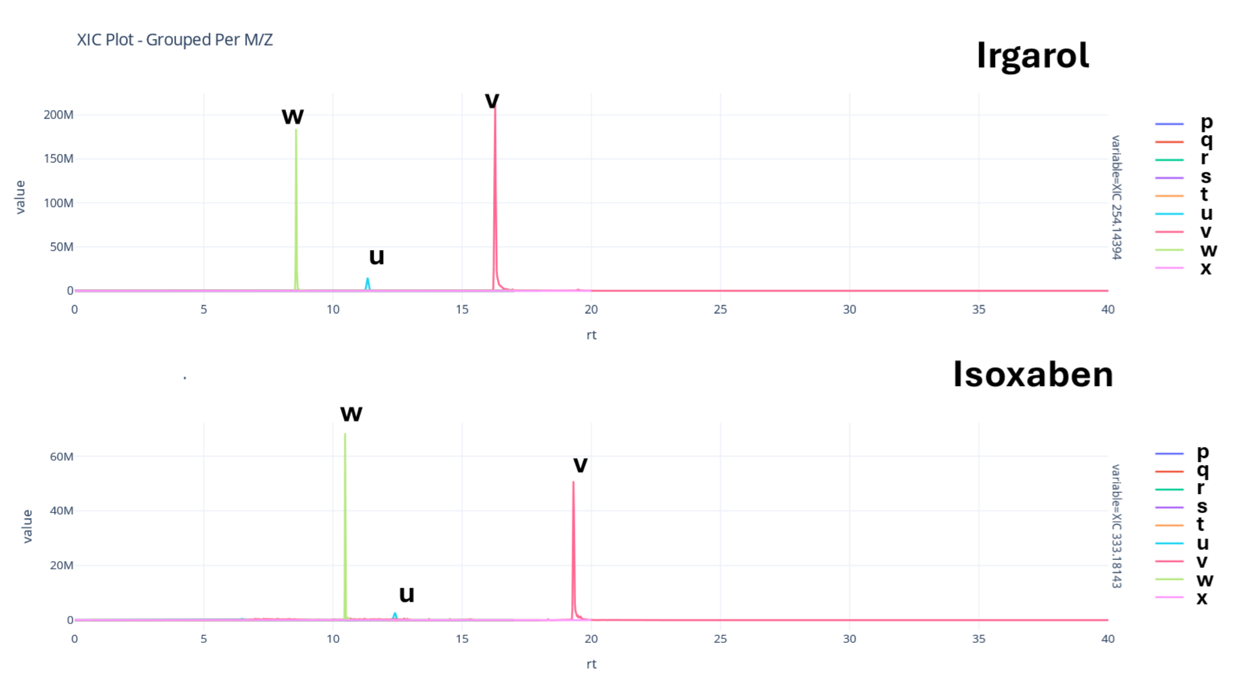


**Figure S11:** XICs of Irgarol and Isoxaben for the excluded datasets.

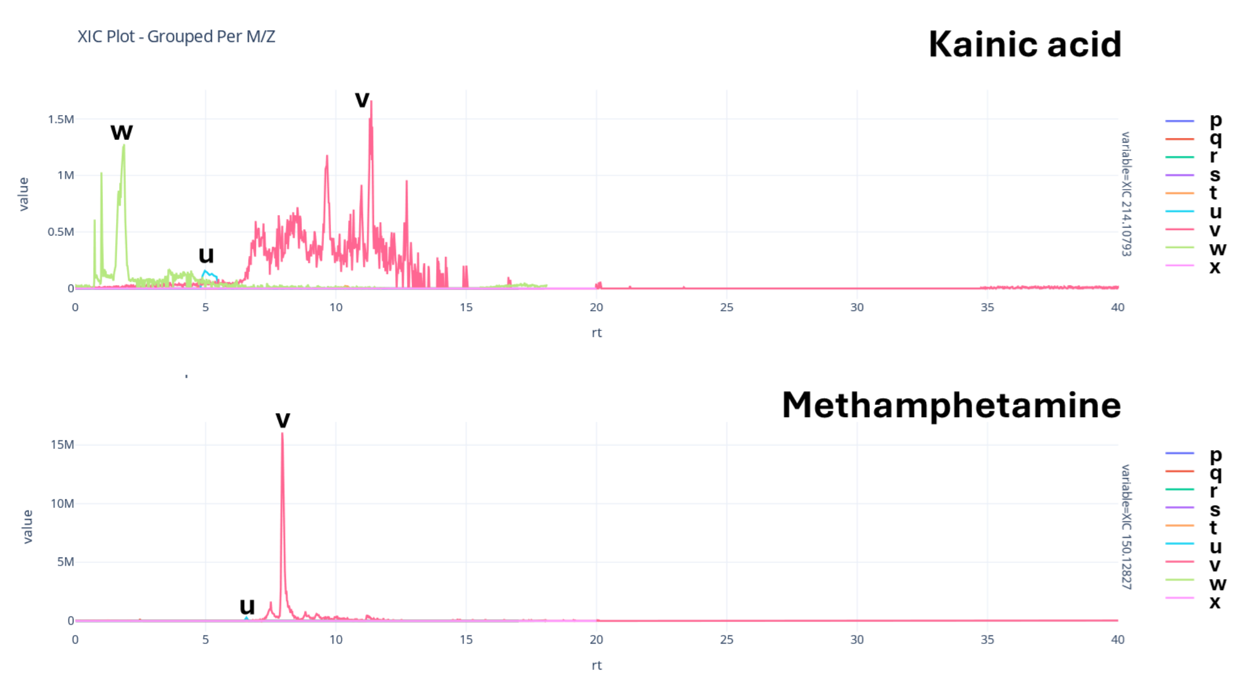


**Figure S12:** XICs of Kainic acid and Methamphetamine for the excluded datasets.

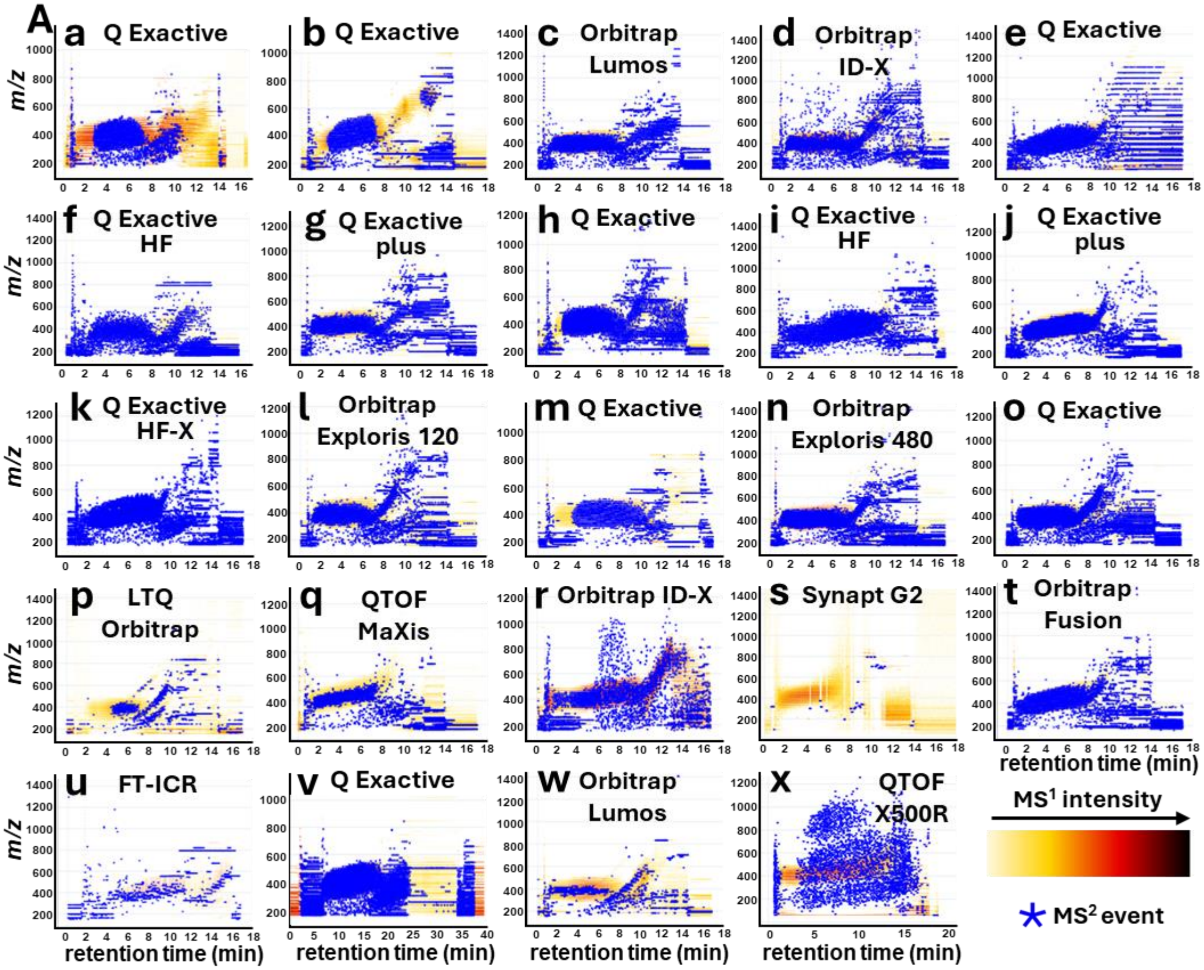


**Figure S13:** LC-MS/MS (ESI-) heatmaps and MS/MS placement for a representative sample (A45M) for each analyzing laboratory. Datasets from laboratories a–o were selected for subsequent unified analysis.

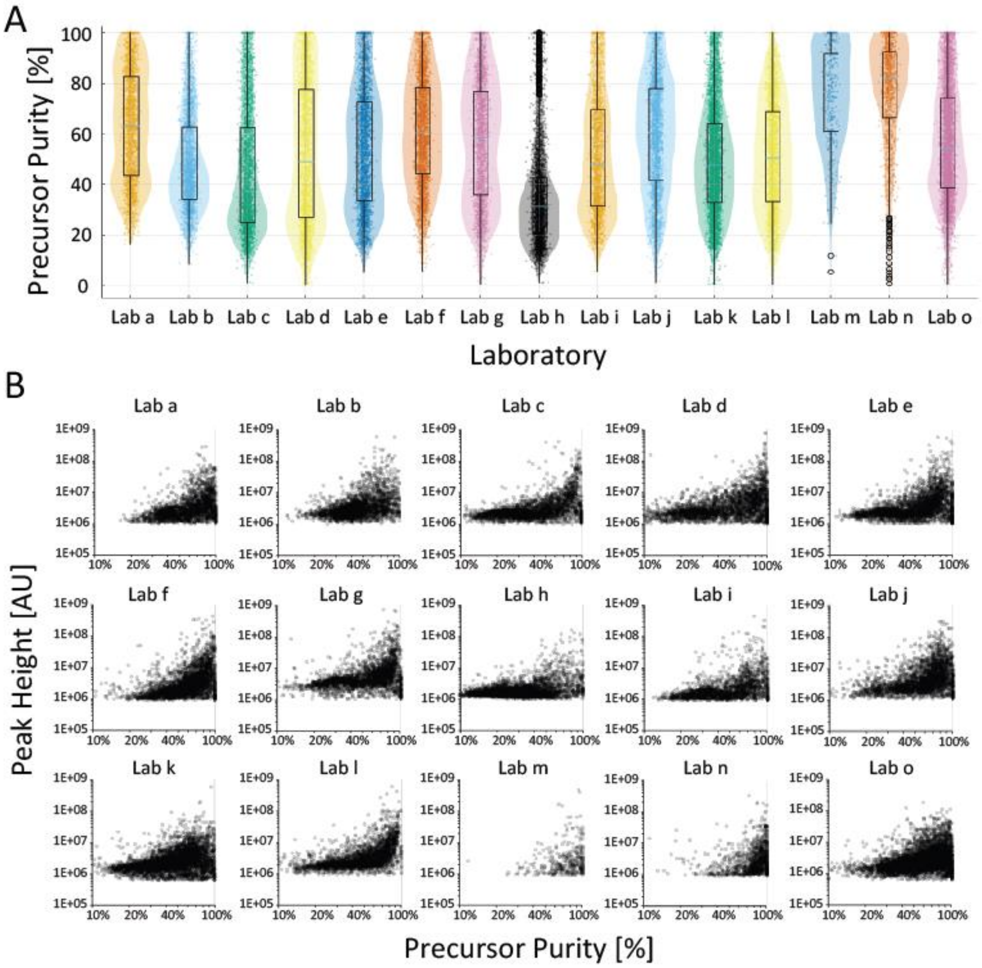


**Figure S14: MS/MS precursor purity.** (A) shows the raincloud plots of the MS/MS precursor purity between the different data sets. (weighted percentage of the desired precursor in the MS/MS isolation window). The violin shapes depict the full data distribution. The box-and-whisker represents the interquartile range, the horizontal line inside the box marks the median purity, and the whiskers indicate the range of non-outlier values. (B) shows scatter plots that display the relationship between precursor purity and peak height in a double logarithmic scale, between the different datasets.

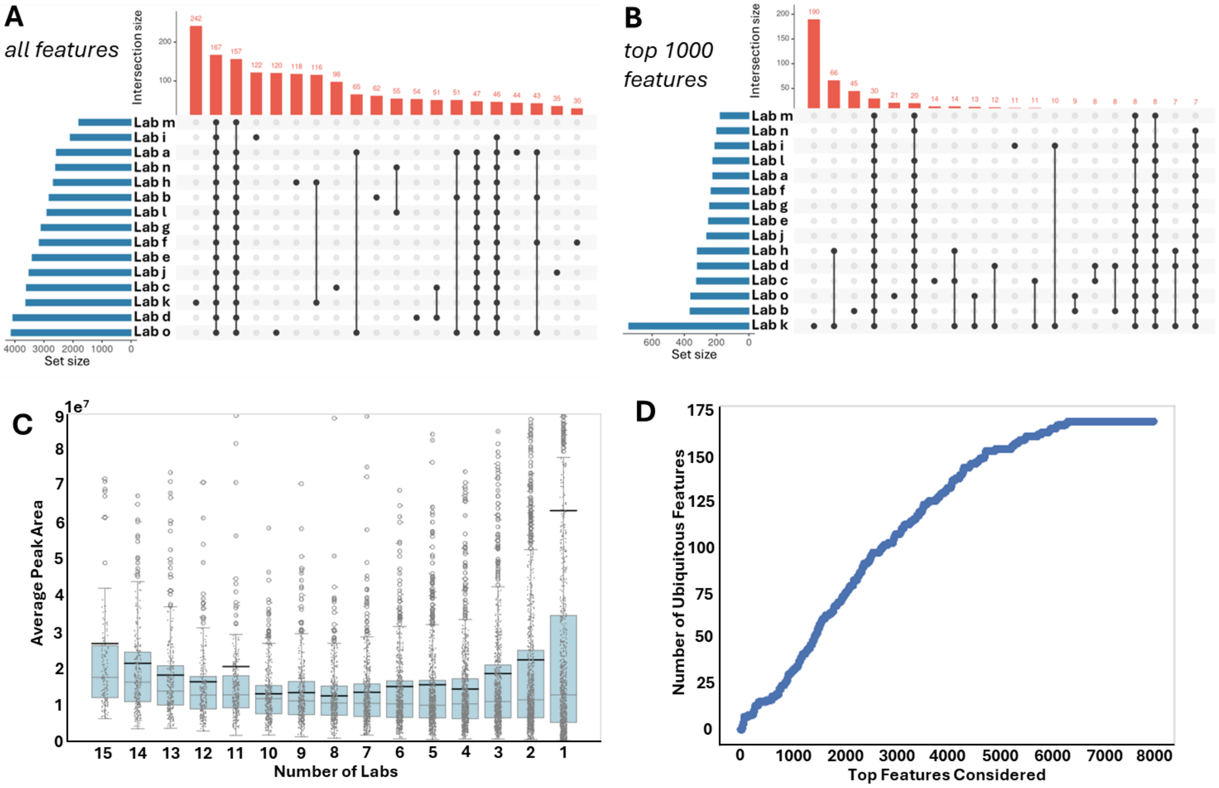


**Figure S15:** Overview of shared LC-MS/MS features and ubiquity across laboratories. LC-MS/MS (ESI+) data was analyzed with Classical Molecular Networking (CMN). (A) UpSet plot illustrating the distribution of shared features across laboratories when considering all detected features. (B) UpSet plot showing shared feature distributions across laboratories, restricted to the top 1000 most intense features. (C) Boxplot showing average peak area as a function of the number of laboratories in which a feature is observed. (D) Cumulative count of features detected in all laboratories, plotted against feature intensity rank.

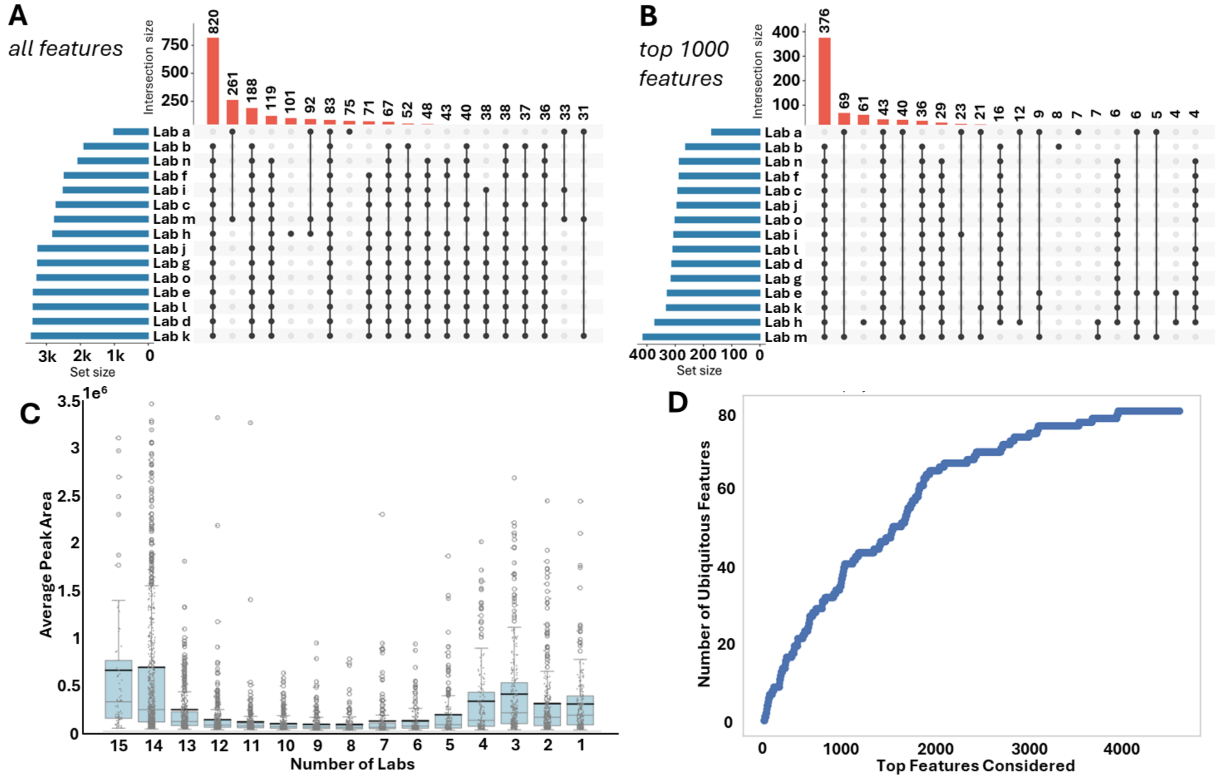


**Figure S16:** Overview of shared LC-MS/MS features and ubiquity across laboratories. LC-MS/MS (ESI-) data was analyzed with Feature Based Molecular Networking (FBMN). (A) UpSet plot illustrating the distribution of shared features across laboratories when considering all detected features. (B) UpSet plot showing shared feature distributions across laboratories, restricted to the top 1000 most intense features. (C) Boxplot showing average peak area as a function of the number of laboratories in which a feature is observed. (D) Cumulative count of features detected in all laboratories, plotted against feature intensity rank.

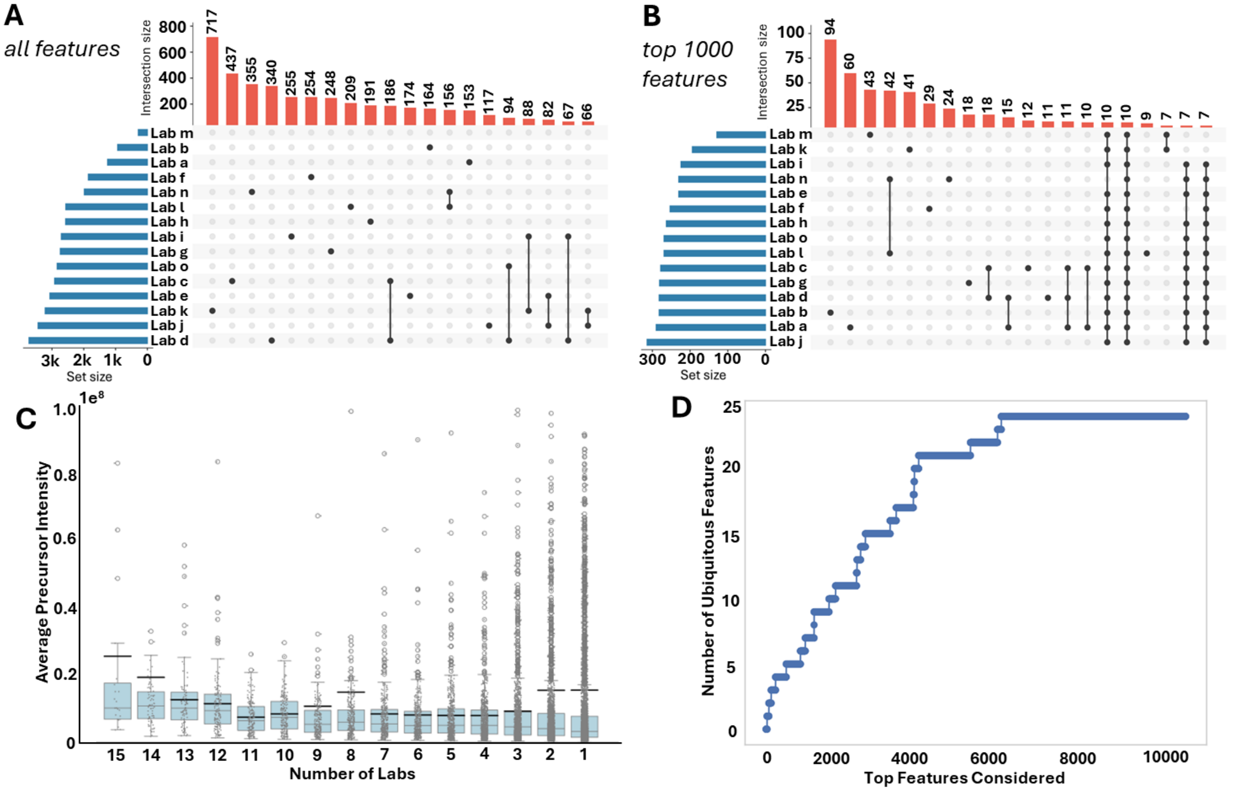


**Figure S17:** Overview of shared LC-MS/MS features and ubiquity across laboratories. LC-MS/MS (ESI-) data was analyzed with Classical Molecular Networking (CMN). (A) UpSet plot illustrating the distribution of shared features across laboratories when considering all detected features. (B) UpSet plot showing shared feature distributions across laboratories, restricted to the top 1000 most intense features. (C) Boxplot showing average peak area as a function of the number of laboratories in which a feature is observed. (D) Cumulative count of features detected in all laboratories, plotted against feature intensity rank.

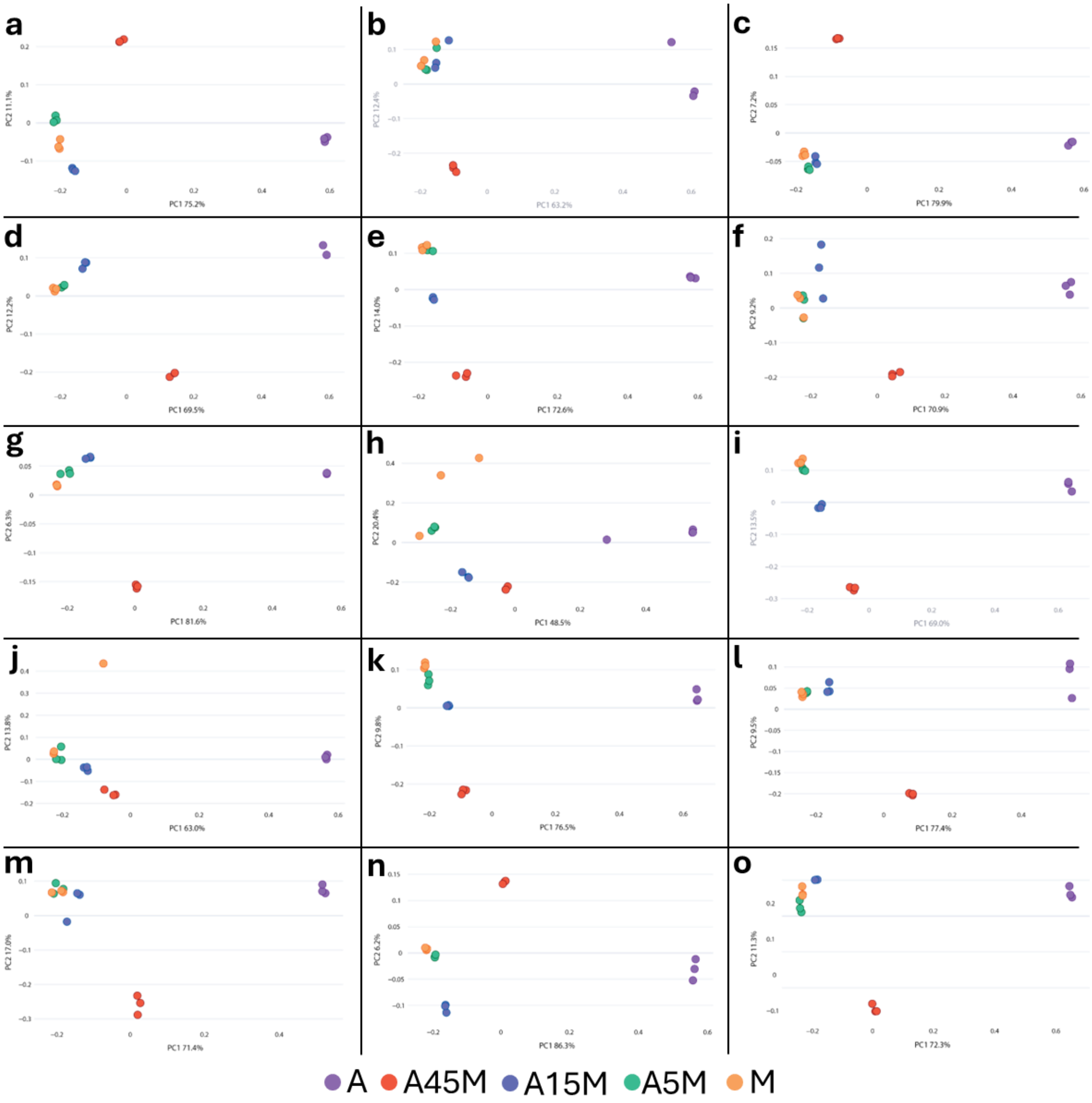


**Figure S18**: FBMN–PCoAs (ESI+) of individual laboratories, all sample types.

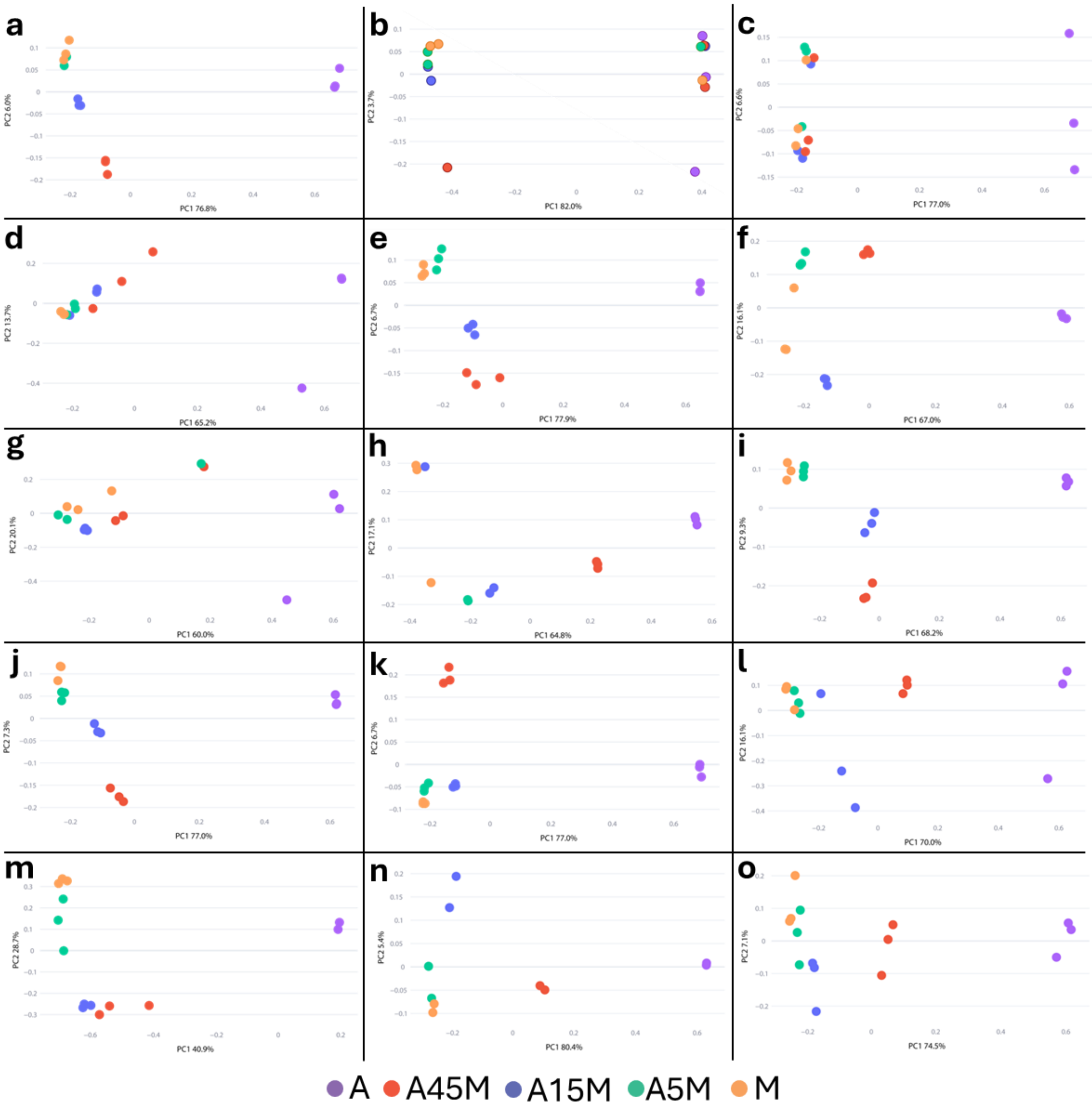


**Figure S19**: FBMN–PCoAs (ESI-) of individual laboratories, all sample types.

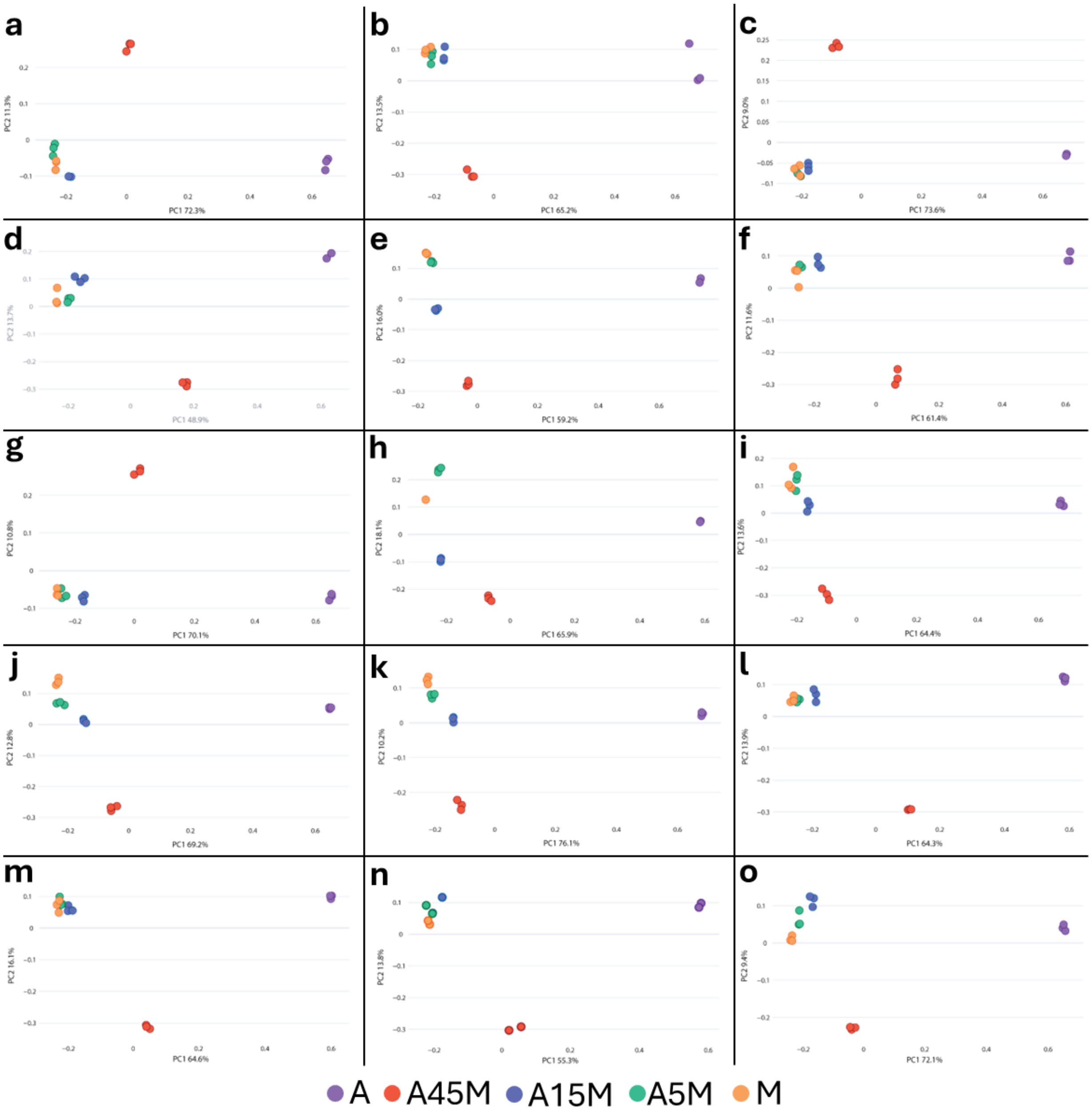


**Figure S20:** CMN–PCoAs (ESI+) of individual laboratories, all sample types.

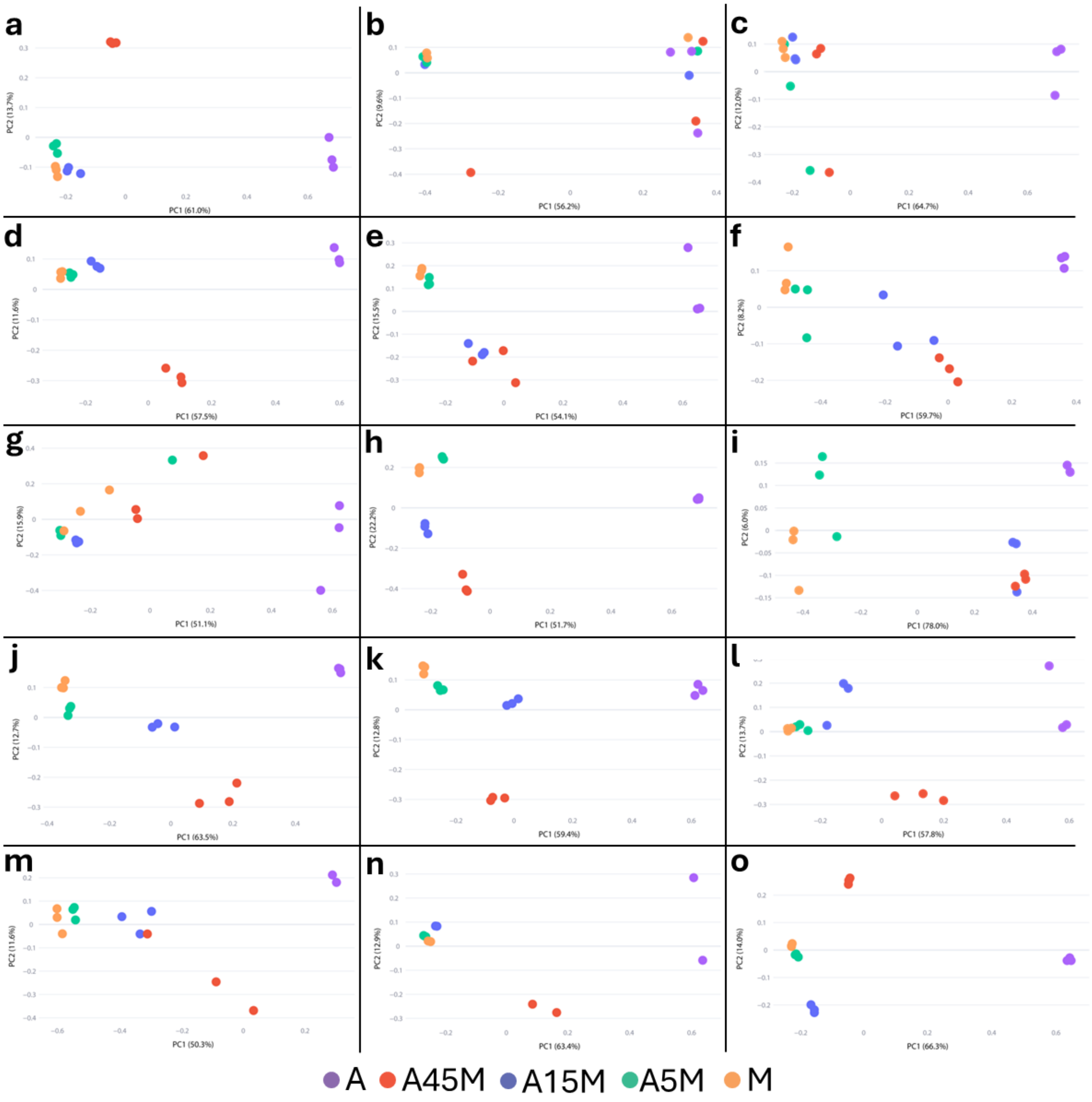


**Figure S21:** CMN–PCoAs (ESI-) of individual laboratories, all sample types.

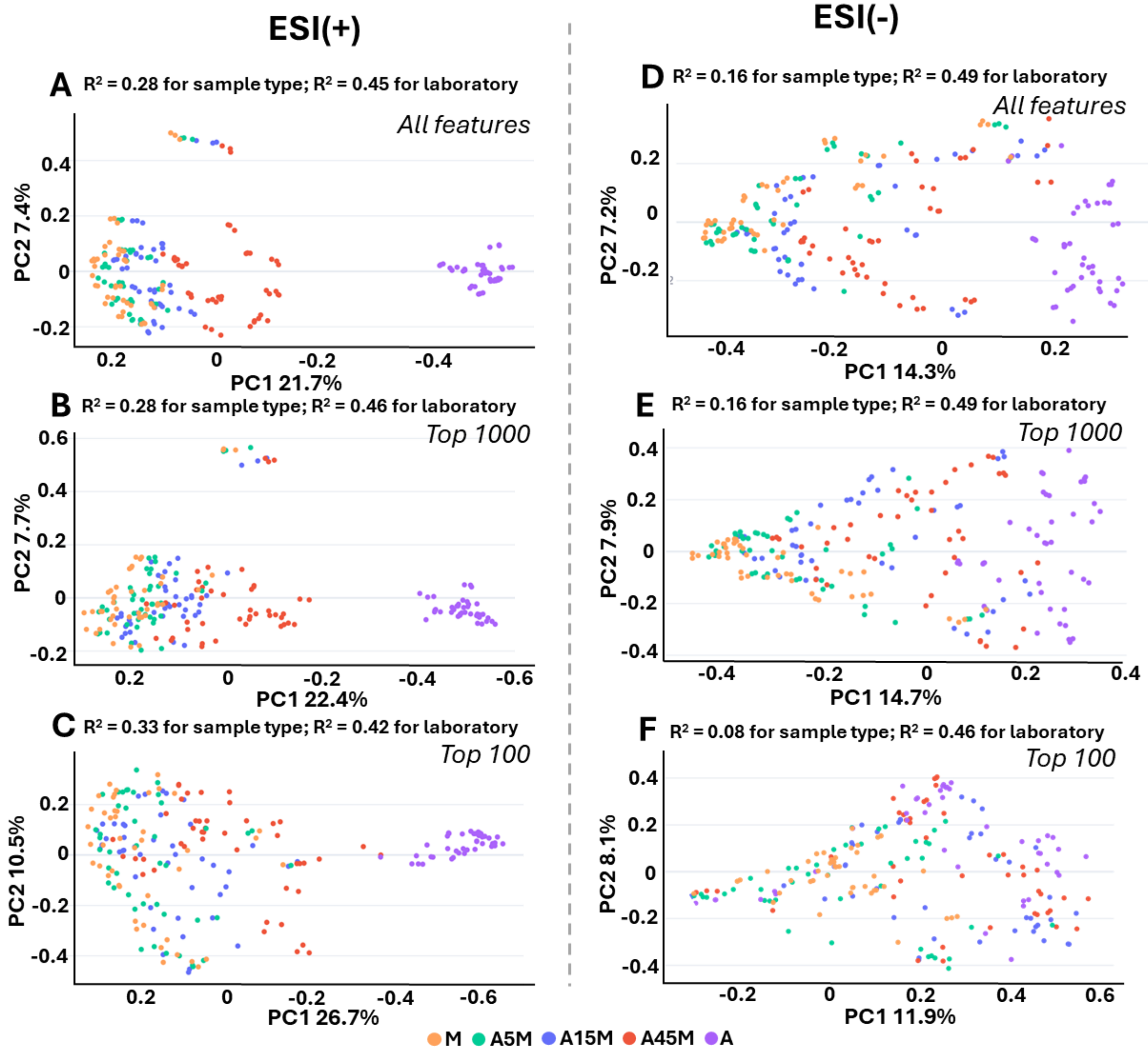


**Figure S22:** A) PCoA and PERMANOVA using Bray-Curtis dissimilarity for the Classical Molecular Networking analysis (CMN, ESI+). B) for the CMN (ESI+) analysis considering only the top 1000 most intense features (by average feature sum); C) for the CMN (ESI+) analysis considering the top 100 features. D) PCoA and PERMANOVA using Bray-Curtis dissimilarity for the Classical Molecular Networking analysis (CMN, ESI-). E) for the CMN (ESI-) analysis considering only the top 1000 most intense features (by average feature sum); F) for the CMN (ESI-) analysis considering the top 100 features. The colors indicate the different extracts. The colors indicate the different extracts.

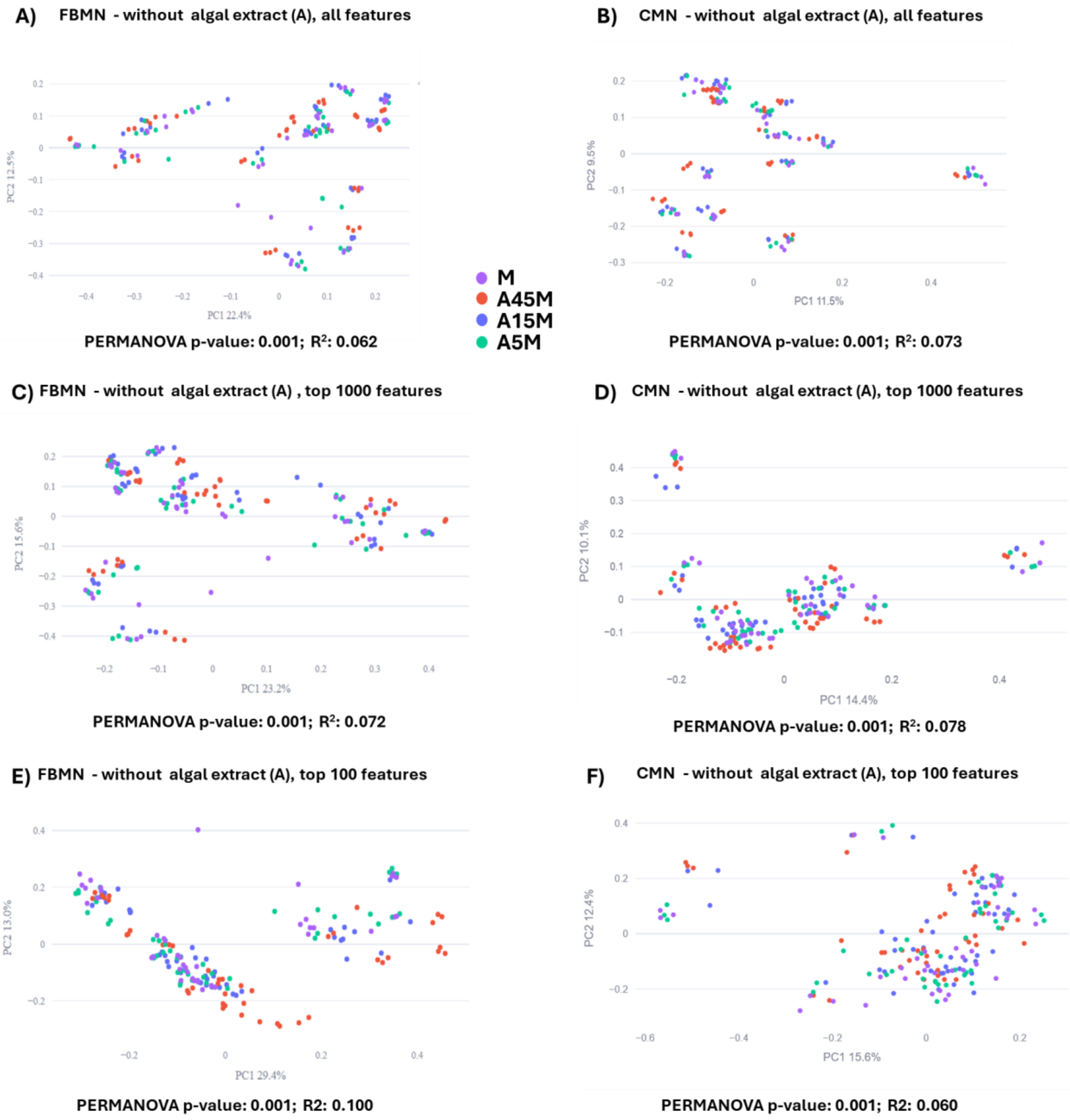


**Figure S23:** A) PCoA and PERMANOVA for the ESI+ Feature-Based Molecular Networking analysis (FBMN) without samples A. A) all features ; B) PCoA and PERMANOVA for Classical Molecular Networking (CMN) without samples A, all features; C) PCoA and PERMANOVA for the FBMN analysis considering the top 1000 features, without samples A; D) PCoA and PERMANOVA for the CMN analysis considering the top 1000 features without samples A; E) PCoA and PERMANOVA for the FBMN analysis considering the top 100 features, without samples A; F) PCoA and PERMANOVA for the CMN analysis considering the top 100 features, without samples A.

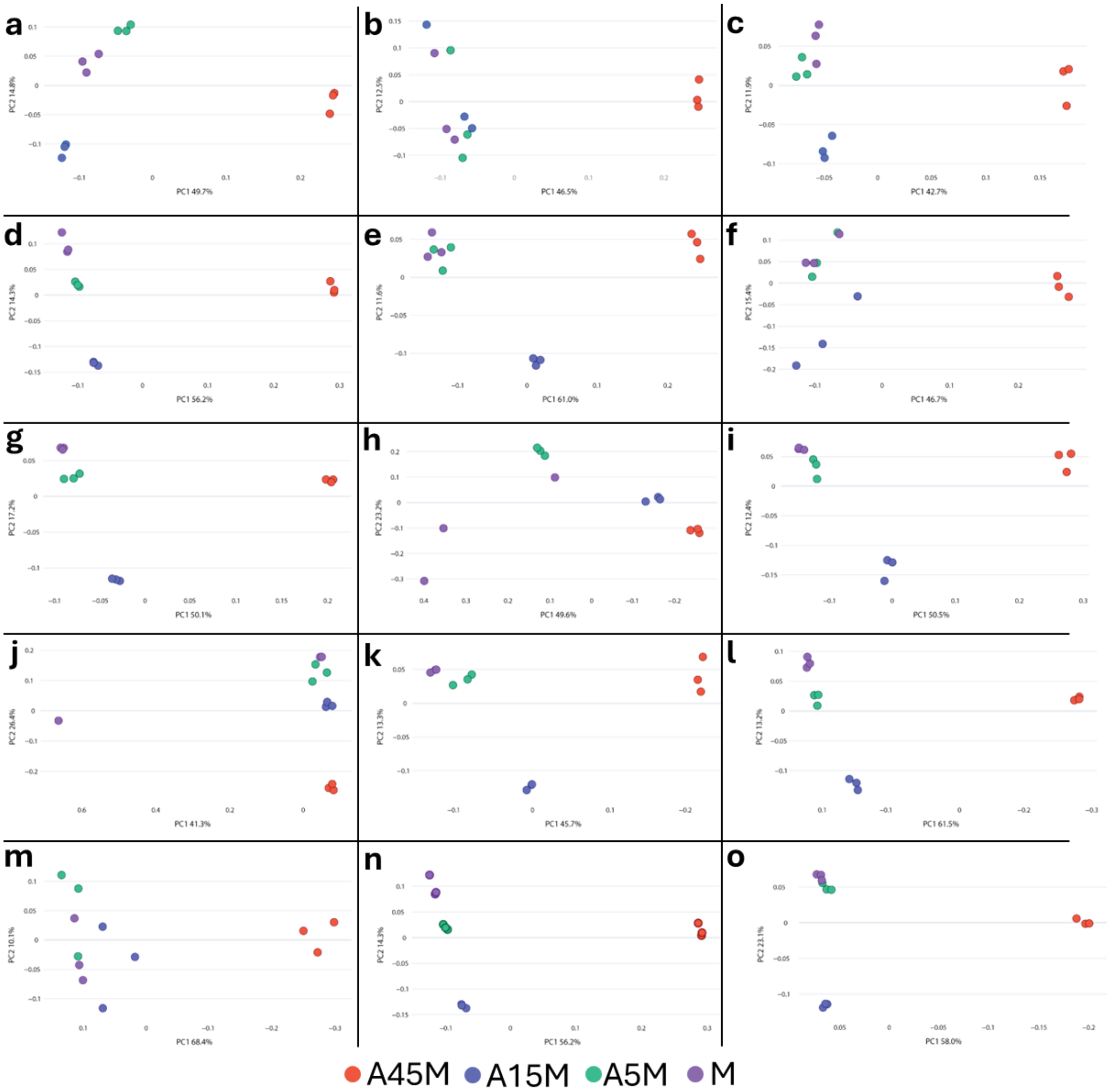


**Figure S24:** FBMN–PCoAs of individual laboratories, without samples A (ESI+).

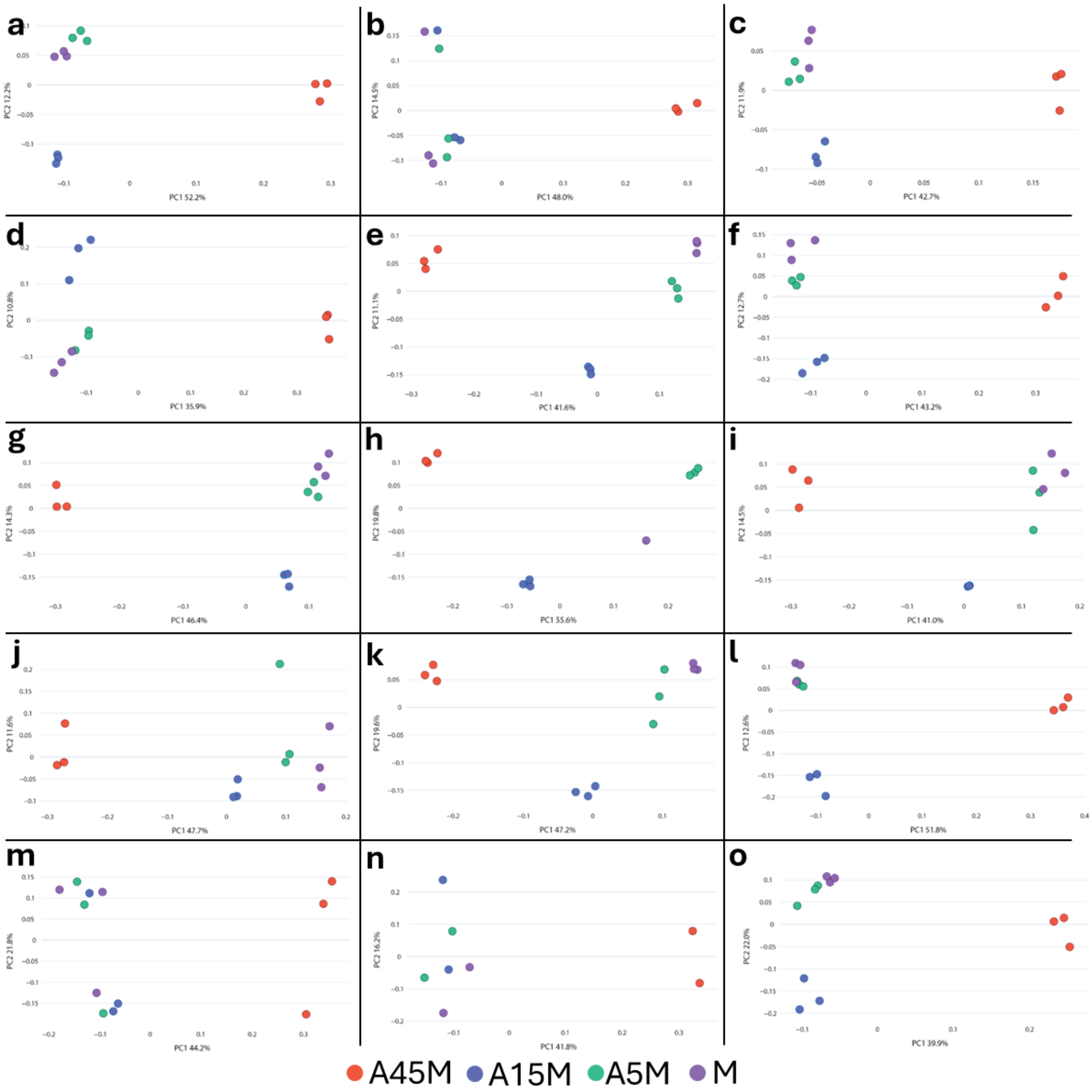


**Figure S25:** CMN–PCoAs of individual laboratories, without samples A (ESI+).

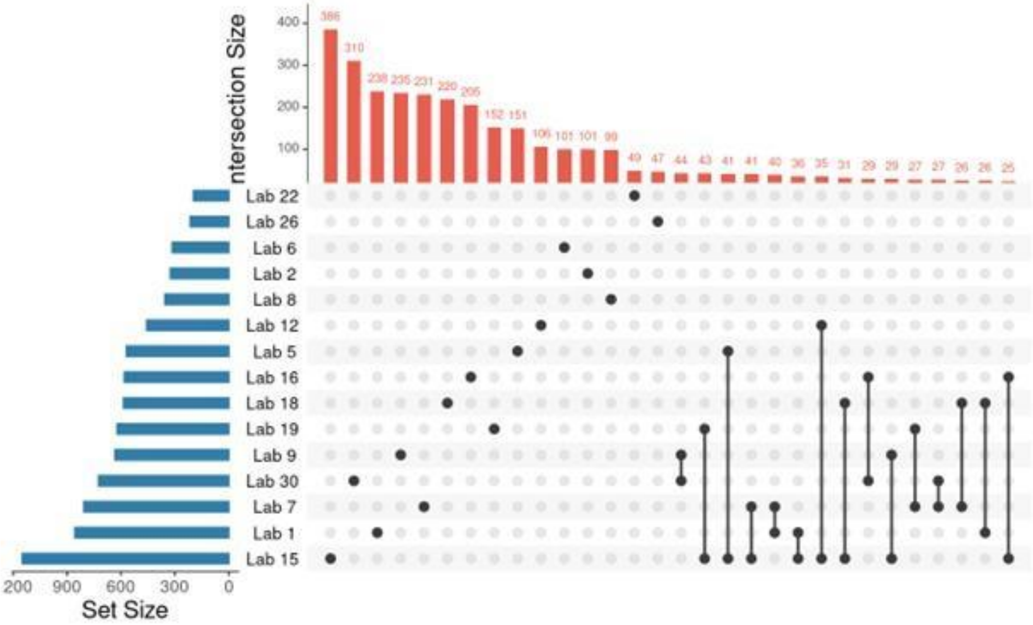


**Figure S26:** Random Forest (RF) analysis results of ESI+ features driving sample type clustering depicted as an UpSet plot showing the overlap of significant features identified by RF analysis across laboratories using feature-based molecular networking (FBMN) without merging of features based on identical spectral library annotation.

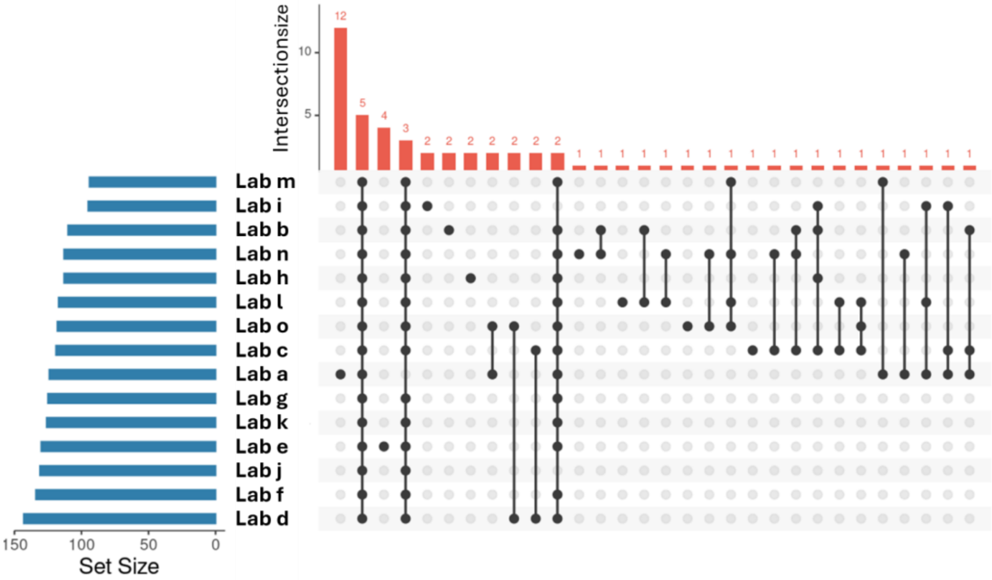


**Figure S27:** Random Forest (RF) analysis of ESI+ features driving sample type clustering. UpSet plot showing the overlap of significant features identified by RF analysis across laboratories using classical molecular networking (CMN) followed by merging of features based on identical spectral library annotation.
